# Naive-like ESRRB^+^ iPSCs with the Capacity for Rapid Neural Differentiation

**DOI:** 10.1016/j.stemcr.2017.10.008

**Published:** 2017-11-09

**Authors:** Fumihiko Kisa, Seiji Shiozawa, Keisuke Oda, Sho Yoshimatsu, Mari Nakamura, Ikuko Koya, Kenji Kawai, Sadafumi Suzuki, Hideyuki Okano

**Affiliations:** 1 Department of Physiology, School of Medicine, Keio University, 35 Shinanomachi, Shinjuku-ku, Tokyo 160-8582, Japan; 2 Discovery Research Laboratories I, Minase Research Institute, Ono Pharmaceutical Co., Ltd., 3-1-1 Sakurai, Shimamoto, Mishima, Osaka 618-8585, Japan; 3 Department of Biomedical Chemistry, Graduate School of Medicine, The University of Tokyo, 7-3-1 Hongo, Bunkyo-ku, Tokyo 113-0033, Japan; 4 Pathological Analysis Center, Central Institute for Experimental Animals, 3-25-12 Tonomachi, Kawasaki, Kanagawa 210-0821, Japan

**Keywords:** naive pluripotency, human iPSC, reprogramming, neural differentiation

## Abstract

Several groups have reported the existence of a form of pluripotency that resembles that of mouse embryonic stem cells (mESCs), i.e., a naive state, in human pluripotent stem cells; however, the characteristics vary between reports. The nuclear receptor ESRRB is expressed in mESCs and plays a significant role in their self-renewal, but its expression has not been observed in most naive-like human induced pluripotent stem cells (hiPSCs). In this study, we modified several methods for converting hiPSCs into a naive state through the transgenic expression of several reprogramming factors. The resulting cells express the components of the core transcriptional network of mESCs, including ESRRB, at high levels, which suggests the existence of naive-state hiPSCs that are similar to mESCs. We also demonstrate that these cells differentiate more readily into neural cells than do conventional hiPSCs. These features may be beneficial for their use in disease modeling and regenerative medicine.

## Highlights

• Induction methods for human naive pluripotent stem cells were optimized
• hiPSCs were reprogrammed to a naive-like state with six transcription factors
• The resulting naive-like hiPSCs expressed naive state-related genes, including *ESRRB*
• The naive-like hiPSCs differentiated readily into neural and glial cells

## Graphical Abstract

**Figure:**
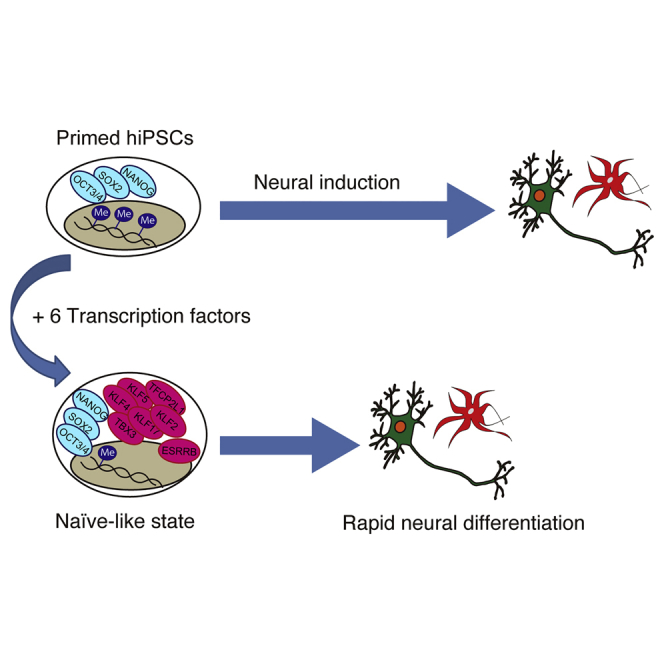

## Teaser

Kisa et al. modified several methods for converting human induced pluripotent stem cells (hiPSCs) into a naive state, a form of pluripotency that exists in mouse embryonic stem cells (ESCs). Converted cells express components of the core transcriptional network upregulated in mouse ESCs, including ESRRB. They also show that these cells differentiate more readily into neural cells than do conventional hiPSCs.

## Introduction

Human pluripotent stem cells (hPSCs), including embryonic stem cells (hESCs) and induced pluripotent stem cells (hiPSCs), exhibit characteristics that are distinct from those of mouse ESCs (mESCs), which are derived from the inner cell mass (ICM) of blastocyst-stage embryos (Thomson et al., 1998, Takahashi et al., 2007, Martin, 1981, Evans and Kaufman, 1981). These different characteristics may reflect differences in developmental stage because hESCs/hiPSCs share many characteristics with mouse epiblast stem cells (mEpiSCs), which are derived from post-implantation embryos (Brons et al., 2007, Tesar et al., 2007). The pluripotent state of mEpiSCs has been called the “primed” state to distinguish it from the “naive” pluripotency of mESCs. Conventional hPSCs have also been characterized as being in the primed state (Nichols and Smith, 2009). Based on the hypothesis that pluripotency state changes as development proceeds, it has been suggested that human cells may exhibit a naive form of pluripotency that corresponds to that of mESCs. Recently, several groups have reported the conversion of hPSCs from a primed to a naive state (Hanna et al., 2010, Gafni et al., 2013, Chan et al., 2013, Takashima et al., 2014, Theunissen et al., 2014, Valamehr et al., 2014, Wang et al., 2014, Ware et al., 2014, Chen et al., 2015, Duggal et al., 2015, Hayashi et al., 2015, Qin et al., 2016). However, due to differences in the methods used and the characteristics of the resulting naive-like cells, the existence of a bona fide naive pluripotent state of human iPSCs remains controversial. For example, human naive-like cells that are converted without the use of transgenes remain dependent on fibroblast growth factor (FGF) or transforming growth factor β (TGF-β) (Chan et al., 2013, Gafni et al., 2013, Valamehr et al., 2014, Ware et al., 2014, Duggal et al., 2015, Qin et al., 2016), which is a major property of the primed state (Vallier et al., 2005). Other groups have reported the conversion of cells from the primed to the naive state using transgenes (Hanna et al., 2010, Takashima et al., 2014, Qin et al., 2016), and the resulting cells appear to be more similar to naive mESCs. Takashima et al. reported the conversion of primed hESCs to a naive state via the transgenic expression of KLF2 and NANOG, both of which have been reported to efficiently reprogram mouse primed PSCs to the naive state (Hall et al., 2009, Silva et al., 2009). However, the resulting cells do not exhibit stable upregulation of ESRRB, which is a member of the core naive pluripotency transcription network in mESCs, in the absence of the continuous expression of the transgenes. Whether the difference between mouse and human ESRRB expression in “naive” pluripotent cells is attributable to interspecies differences or insufficient reprogramming remains controversial. Furthermore, the practical advantages of reprogrammed naive human pluripotent cells also remain unclear because the differentiation potential of human naive cells into specific lineages is not well known.

In the present study, we modified several conversion methods for the reprogramming of hiPSCs to a naive-like state using a combination of transcription factors and culture conditions. The reprogrammed cells exhibit elevated expression of the full set of naive pluripotency-related transcription factors that is known in mESCs, including *ESRRB*, which suggests the existence of a state of naive pluripotency in human cells that more closely resembles that of mESCs compared with the cells in previous reports. We also demonstrate that these naive-like hiPSCs exhibit enhanced abilities to differentiate into neural cells.

## Results

### Robust Reprogramming of hiPSCs to a Naive-like State via the Expression of Six Transcription Factors

**Figure 1:**
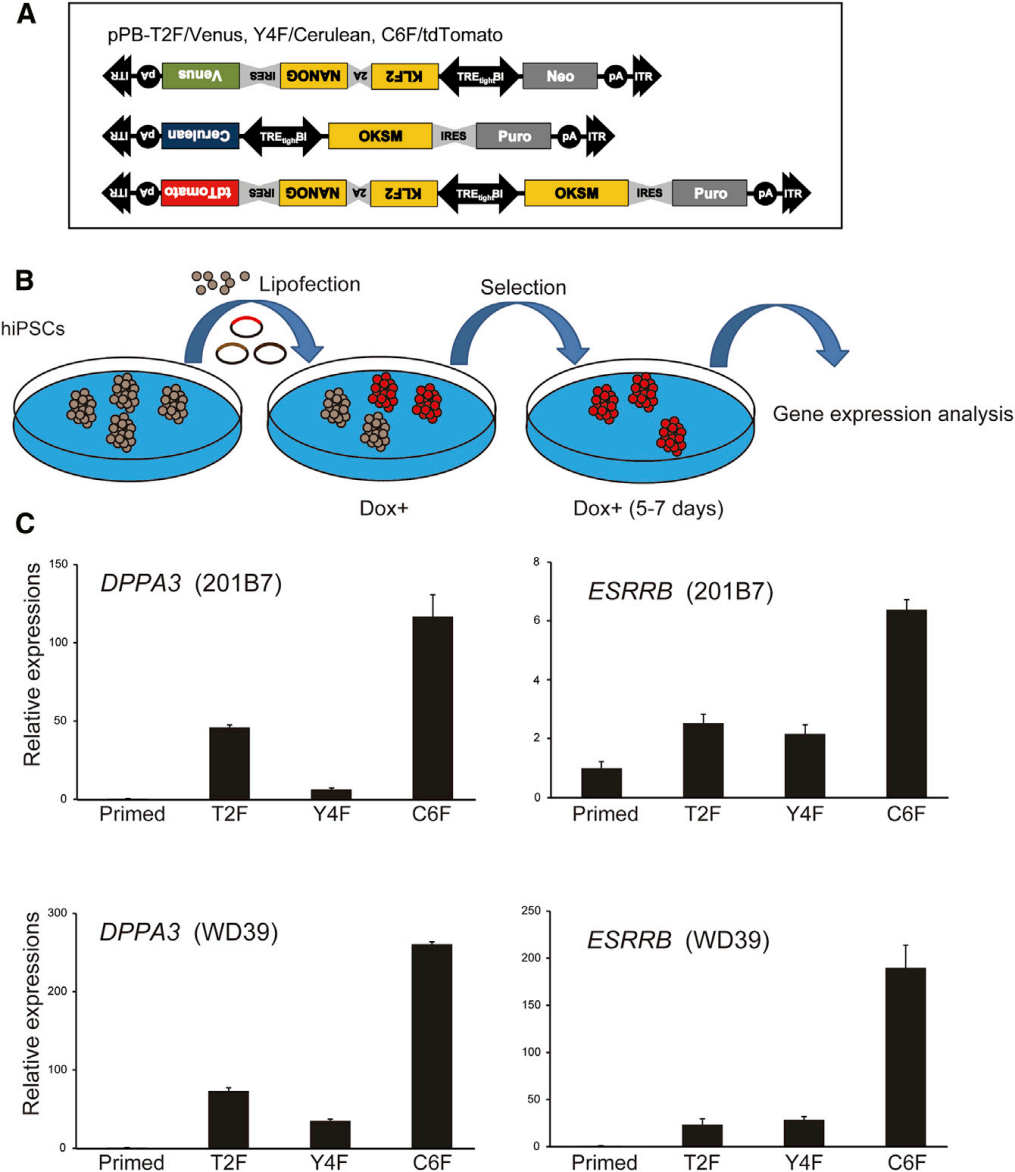
A Set of Reprogramming Factors for Robust Conversion to Naive State Pluripotency (A) Constructs used to express the reprogramming factors. ITR, inverted terminal repeat; pA, polyadenylation signal; TRE, tetracycline response element; Neo, neomycin-resistance gene; Puro, puromycin-resistance gene; IRES, internal ribosome entry sites; OKSM, the set of *OCT3/4*, *KLF4*, *SOX2*, and *c-MYC*. (B) Schematic presentation of the method used to evaluate the reprogramming factors. The red colonies represent the expression of Tomato, suggesting the exogenous expression of C6F. (C) qPCR analyses of the expressions of the naive marker genes (*DPPA3* and *ESRRB*) in the reprogrammed 201B7 and WD39 cells suggesting a robust reprogramming with C6F compared with T2F and Y4F (n = 3, mean ± SEM; independent experiments). See also Figure S1.

The co-expressions of *KLF2* and *KLF4*, *OCT3/4* and *KLF4*, and *KLF2* and *NANOG* have all been used to reprogram human pluripotent cells to a naive-like state (Hanna et al., 2010, Takashima et al., 2014). The evidence for conversion to a naive state via the combination of KLF2 and NANOG is particularly compelling (Hall et al., 2009, Silva et al., 2009, Theunissen et al., 2014, Takashima et al., 2014). In the present study, we assessed the efficiency of reprogramming methods involving three sets of transcription factors, i.e., the so-called Takashima two-factor (T2F: *KLF2* and *NANOG*), the Yamanaka four-factor (Y4F: *OCT3/4*, *KLF4*, *SOX2*, and *c-MYC*), and the combinatorial six-factor (C6F: *OCT3/4*, *KLF4*, *SOX2*, *c-MYC*, *KLF2*, and *NANOG*) methods, in hiPSCs (Figure 1A). We introduced a doxycycline (Dox)-inducible piggyBac transposon vector carrying T2F/Venus, Y4F/Cerulean, or C6F/TdTomato along with reverse tetracycline transactivator (rtTA) and piggyBac transposase expression vectors (Yusa et al., 2011) into two hiPSC lines, i.e., 201B7 and WD39 (Figure 1B). After adding Dox, we selected transgene-expressing cells grown in conventional hESC culture medium, and measured the expressions of *ESRRB* and *DPPA3*, which are highly expressed in naive mESCs but not in primed mEpiSCs, as naive marker genes. The expressions of *ESRRB* and *DPPA3* were most highly elevated in the C6F-expressing cells, although the expressions of these genes were also elevated relative to the primed-state counterparts via the ectopic expression of T2F alone (Figure 1C). These findings suggest a cooperative effect of T2F and Y4F on naive conversion. We also compared our C6F transgene method with the transgene-free culture methods described by Gafni et al. (2013) (NHSM) and Theunissen et al. (2014) (6i/L/A). Similar to the reports of these authors, the expression of *DPPA3* was elevated in the 6i/L/A condition but not in the NHSM condition, and the expression of *ESRRB* was not elevated in either condition (Figure S1A). These results suggest that reprogramming to a bona fide naive state requires transgene expression and that C6F is the most powerful set of transcription factors for this purpose, especially in terms of the upregulation of naive marker gene expression.

### mESC-like Growth Properties of Naive-like hiPSCs

To further evaluate the characteristics of the naive-like hiPSCs that were converted by the C6F method, we established two transgenic lines harboring C6F/TdTomato and rtTA transgenes. In brief, hiPSC lines 201B7 and WD39 were transfected with a Dox-inducible piggyBac transposon vector carrying C6F/TdTomato and rtTA along with a transposase expression vector. These cells were selected with hygromycin under culture conditions conventionally used for primed pluripotent cells. After transient treatment with Dox, suitable colonies were selected based on Dox-inducible TdTomato expression and expanded by subculturing. The cells were maintained in the primed condition without Dox (P-hiPSCs).

**Figure 2:**
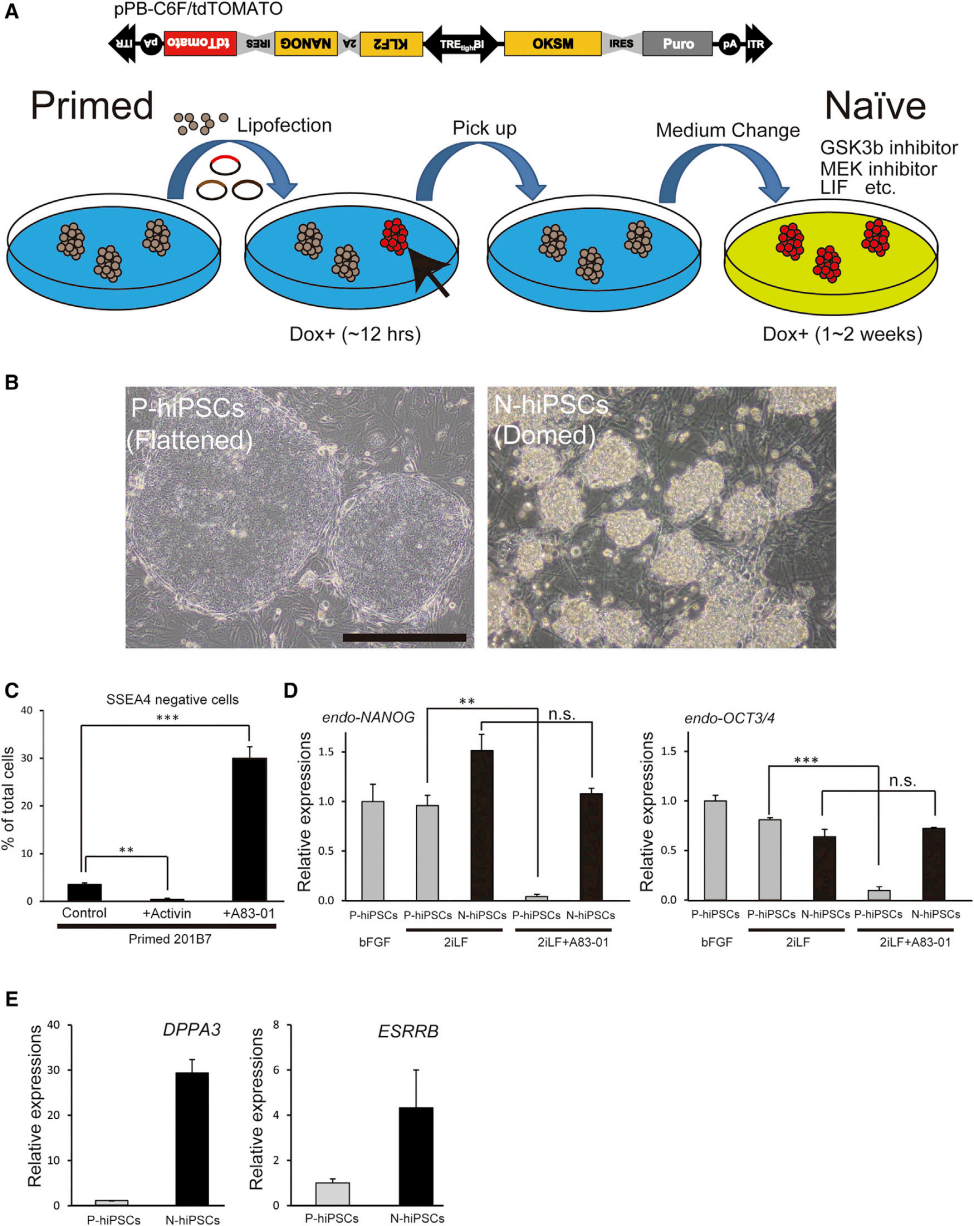
Generation of the Naive-like hiPSC Lines with the piggyBac Transposon (A) Schematic presentation of the protocol for the generation of naive-like hiPSC lines via the piggyBac transposon. The black arrow indicates a transgene-expressing colony. (B) Phase images of a clonal line of transgenic 201B7 hiPSCs cultured in a conventional primed condition without Dox (P-hiPSCs) and in 2iLF with Dox (N-hiPSCs). The N-hiPSCs formed dome-shaped colonies. Scale bar, 500 μm. (C) TGF-β/Activin signal responsivity of the conventional hiPSCs. Control iPSC clones (201B7) cultured with activin or A83-01, which is a TGF-β/Activin signal inhibitor, were subjected to FACS analysis for the expression of SSEA-4, which is a marker of pluripotency (n = 3, mean ± SEM; independent experiments; ^∗∗^p < 0.01; ^∗∗∗^p < 0.001; t test). Activin supports the pluripotency of primed cells, whereas A83-01 deteriorates it. (D) TGF-β/Activin signal independency of the reprogrammed cells. P-hiPSCs (without Dox) and N-hiPSCs (with Dox) were cultured in 2iLF, which is a medium that contains LIF, PD0325901, CHIR99021, and forskolin, with or without A83-01. The endogenous expressions of the pluripotency marker genes (*OCT3/4* and *NANOG*) were analyzed by qPCR (n = 3, mean ± SEM; independent experiments; ^∗∗^p < 0. 01; ^∗∗∗^p < 0.001; n.s., not significant; t test). (E) qPCR analyses of naive marker gene (*DPPA3* and *ESRRB*) expressions in the 201B7 P-hiPSCs and N-hiPSCs (n = 3, mean ± SEM; independent experiments).

For the conversion to a naive pluripotent state, the cells were cultured in a medium containing the cytokine leukemia inhibitory factor (LIF), Dox, and cocktail of small molecules (N-hiPSCs) (Figure 2A). Most of the naive-like human ESCs/iPSCs that have been reported to date have been maintained in medium containing 2iL, a combination of MEK (mitogen-activated protein kinase kinase) and GSK3 (glycogen synthase kinase 3) inhibitors with LIF (Ying et al., 2008, Marks et al., 2012). Initially, we also regarded 2iL as essential to the maintenance of naive-like pluripotent cells and thus added it to the KSR (KnockOut serum replacement)-based medium. We also added forskolin to the 2iL (2iLF) based on previously reported methods (Hanna et al., 2010, Duggal et al., 2015, Qin et al., 2016). The N-hiPSCs cultured in 2iLF exhibited a tightly packed domed morphology (Figure 2B). Additionally, similar to mESCs, the N-hiPSCs could be passaged as single cells using trypsin/EDTA without the addition of a ROCK inhibitor (Figure S2) (Watanabe et al., 2007).

### TGF-β/Activin Signal Independence of C6F-Expressing hiPSCs

The promotion of self-renewal by TGF-β/Activin signaling has been regarded as a characteristic of primed pluripotent stem cells. To explore the differences in the growth factor requirements of P-hiPSCs and N-hiPSCs, we applied activin or A83-01, which is a pharmacological inhibitor of TGF-β/Activin signals, to P-hiPSCs for 5 days and analyzed the expression of SSEA4, which is a cell-surface marker of pluripotent stem cells, via flow cytometry. As expected, the number of SSEA4-positive cells increased following the addition of activin and decreased following treatment with an activin inhibitor (Figure 2C), which indicates that the P-hiPSCs exhibited characteristics consistent with primed-state pluripotency. Next, we examined the responsivity of N-hiPSCs to TGF-β/Activin signaling. We cultured N-hiPSCs in 2iLF in the presence of A83-01 (2iLFA) for 5 days and performed qPCR analysis. The expressions of endogenous pluripotency markers were sustained at considerable levels in the N-hiPSCs with 2iLFA, although the levels in the P-hiPSCs were dramatically decreased (Figure 2D). Additionally, the N-hiPSCs cultured in 2iLFA maintained high expression levels of the naive marker genes *ESRRB* and *DPPA3* (Figures 2E and S3A–S3C). These data indicate that N-hiPSCs but not P-hiPSCs can be maintained without TGF-β/Activin signaling.

### Three-Germ-Layer Differentiation Potential of Naive-like hiPSCs

To confirm the differentiation potential of the N-hiPSCs, we performed embryoid body formation assays and observed that both the P-hiPSCs and N-hiPSCs were able to differentiate into all three germ layers *in vitro* (Figure S4A). Furthermore, the differences of the differentiation potentials between P-hiPSCs and N-hiPSCs were evaluated with the TaqMan hPSC Scorecard assay (Tsankov et al., 2015). Results from the assay suggested that N-hiPSCs had a higher three-germ-layer differentiation potential (Figures S4B–S4D).

These results indicate that N-hiPSCs exhibit the capacity for differentiation with high levels of naive marker gene expression.

### Optimization of the Culture Conditions

**Figure 3:**
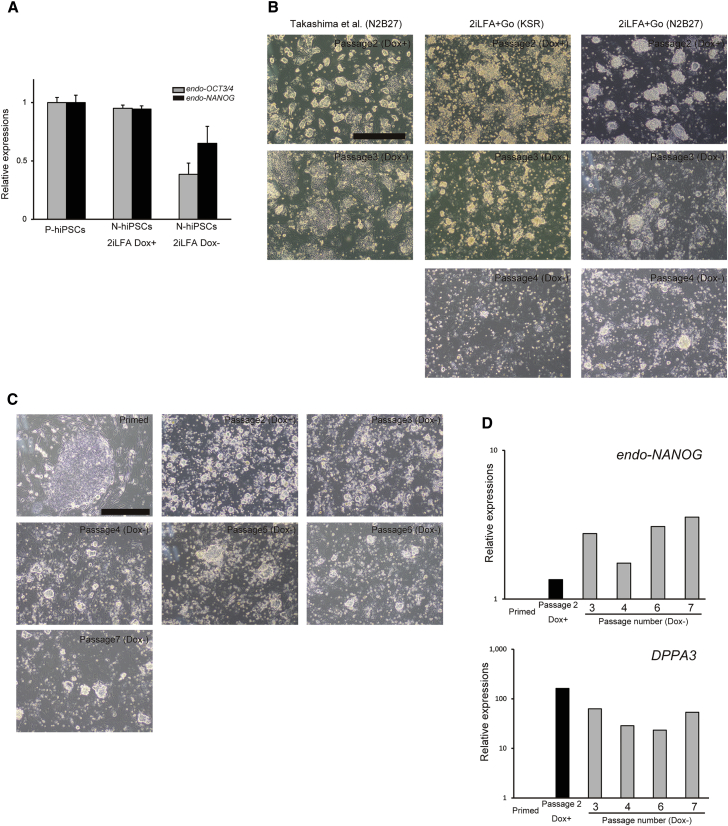
Culture Conditions for the Naive-like hiPSCs (A) qPCR analyses of pluripotency marker gene (*OCT3/4* and *NANOG*) expressions in naive-like cells (201B7 N-hiPSCs) cultured in 2iLFA before and after the withdrawal of Dox, and in primed cells (201B7 P-hiPSCs) (n = 3, mean ± SEM; independent experiments). (B) Phase images of naive-like cells (201B7 N-hiPSCs) cultured in three different media before and after the withdrawal of Dox. 2iLFA + Go (KSR), KSR-based medium supplemented with 2iL, forskolin, TGF-β inhibitor, and PKC inhibitor; 2iLFA + Go (N2B27), N2B27-based medium supplemented with 2iL, forskolin, TGF-β inhibitor, and PKC inhibitor; Takashima et al. (N2B27), N2B27-based medium supplemented with 2iL and PKC inhibitor. Scale bar, 500 μm. (C) Phase images of 201B7 N-hiPSCs maintained in 2iLFA + Go (N2B27) through passages. Dox was withdrawn at passage 3. Scale bar, 500 μm. (D) qPCR analysis of *DPPA3* and *NANOG* expressions in the 201B7 N-hiPSCs through the passages.

Whereas naive pluripotency was stably maintained in the N-hiPSCs in the presence of Dox, it became unstable after withdrawal of Dox (Figure 3A). Hence, we tested to identify the optimal medium conditions that were able to stably maintain the pluripotency of the N-hiPSCs after the removal of Dox (Figure 3B). First, we used an N2B27-based medium supplemented with 2iL and a protein kinase C (PKC) inhibitor (Gö6983) as previously reported (Dutta et al., 2011, Takashima et al., 2014). However, the N-hiPSC colonies reverted to a flattened morphology upon the withdrawal of Dox. Next, we examined the combination of our previous medium conditions and Gö6983. Although domed-colony morphologies could be maintained in the absence of Dox in these conditions, the cells could not be repeatedly propagated due to low viability. Therefore, we further tested a number of modifications of our culture with the aim of improving viability. Alteration of the basal medium to an N2B27-based medium from a KSR-based medium dramatically improved cell viability. The N-hiPSCs could be passaged while maintaining the domed-colony morphology after the withdrawal of Dox (Figure 3C). Furthermore, qPCR analysis revealed that the expressions of pluripotency markers and naive markers were also maintained after the withdrawal of Dox (Figure 3D). The precise control of transgene expression by Dox was confirmed by qPCR (Figure S1D), as well as fluorescence-activated cell sorting (FACS) analysis of TdTomato expression (Figures S1B and S1C). Moreover, we confirmed that the N-hiPSCs that were cultured in N2B27-based 2iLFA medium supplemented with Gö6983 were also capable of differentiating into all three germ layers (Figure S4A).

### LIF/Stat3 Signal Dependence of N-hiPSCs

**Figure 4:**
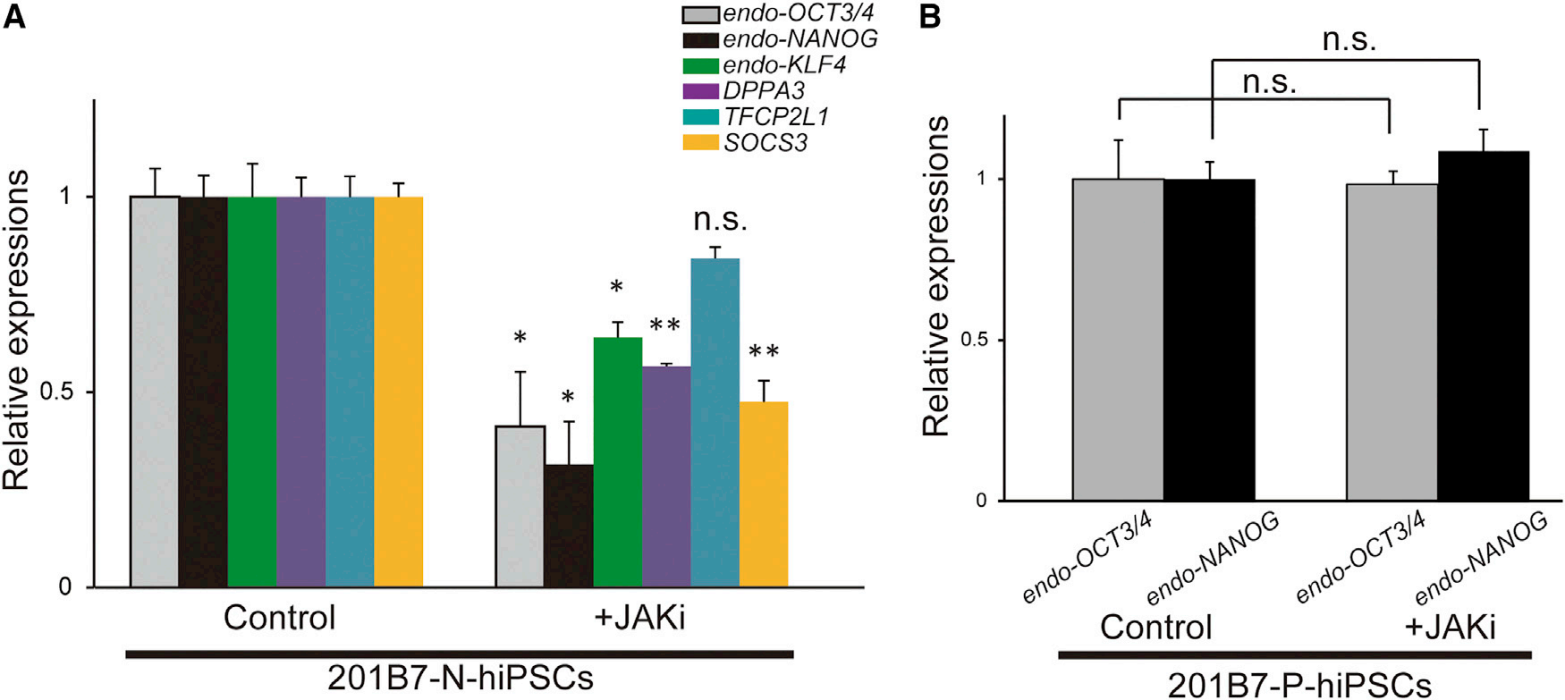
Jak/Stat3 Signal Responsivity qPCR analysis of the 201B7 N-hiPSCs (A) and P-hiPSCs (B) with and without a Jak inhibitor (Jaki) for the endogenous expressions of Jak/Stat3 signaling-related genes and pluripotency-related genes (n = 3, mean ± SEM; independent experiments; ^∗^p < 0.05; ^∗∗^p < 0.01; n.s., not significant; t test).

It is well known that naive mESCs require LIF/JAK/STAT signaling for sustained self-renewal (Smith et al., 1988, Williams et al., 1988, Niwa et al., 2009). To identify the differences in the role of the LIF signal in human naive and primed states, we treated N-hiPSCs and P-hiPSCs with a JAK inhibitor (JAK Inhibitor I) and evaluated the effects via qPCR analysis. In the N-hiPSCs, the expression levels of pluripotency markers were significantly decreased upon inhibition of JAK1 (Figure 4A), whereas these levels were not altered in the P-hiPSCs (Figure 4B). Moreover, the expressions of naive marker genes (*DPPA3*) and a downstream target gene of the LIF signal (*SOCS3* and *KLF4*) were also downregulated by JAK1 inhibition in the N-hiPSCs, whereas the expression of *TFCP2L1* was unaffected. As previously reported in mESCs, CHIR99021 treatment may have compensated for the TFCP2L1 expression (Ye et al., 2013, Martello et al., 2013).

These results suggested the existence of an mESC-like JAK/STAT signal circuit in the N-hiPSCs. Taken together with the TGF-β/Activin signal independence, we concluded that the N-hiPSCs exhibited growth factor requirements that were highly similar to those of mESCs.

### Alteration of TFE3 Subcellular Localization

TFE3 is distributed in both the nucleus and cytoplasm in naive mESCs, while nuclear TFE3 translocates into the cytoplasm at the onset of mESC differentiation (Betschinger et al., 2013). Similar to mouse cells, TFE3 has been demonstrated to be enriched in the nucleus when human primed cells are converted to the naive state (Gafni et al., 2013, Takashima et al., 2014). We confirmed that our N-hiPSCs also exhibited a nuclear localization of TFE3 that contrasted with the cytoplasmic localization observed in the P-hiPSCs according to the immunocytochemical analysis (Figure S3D).

### Gene Expression

**Figure 5:**
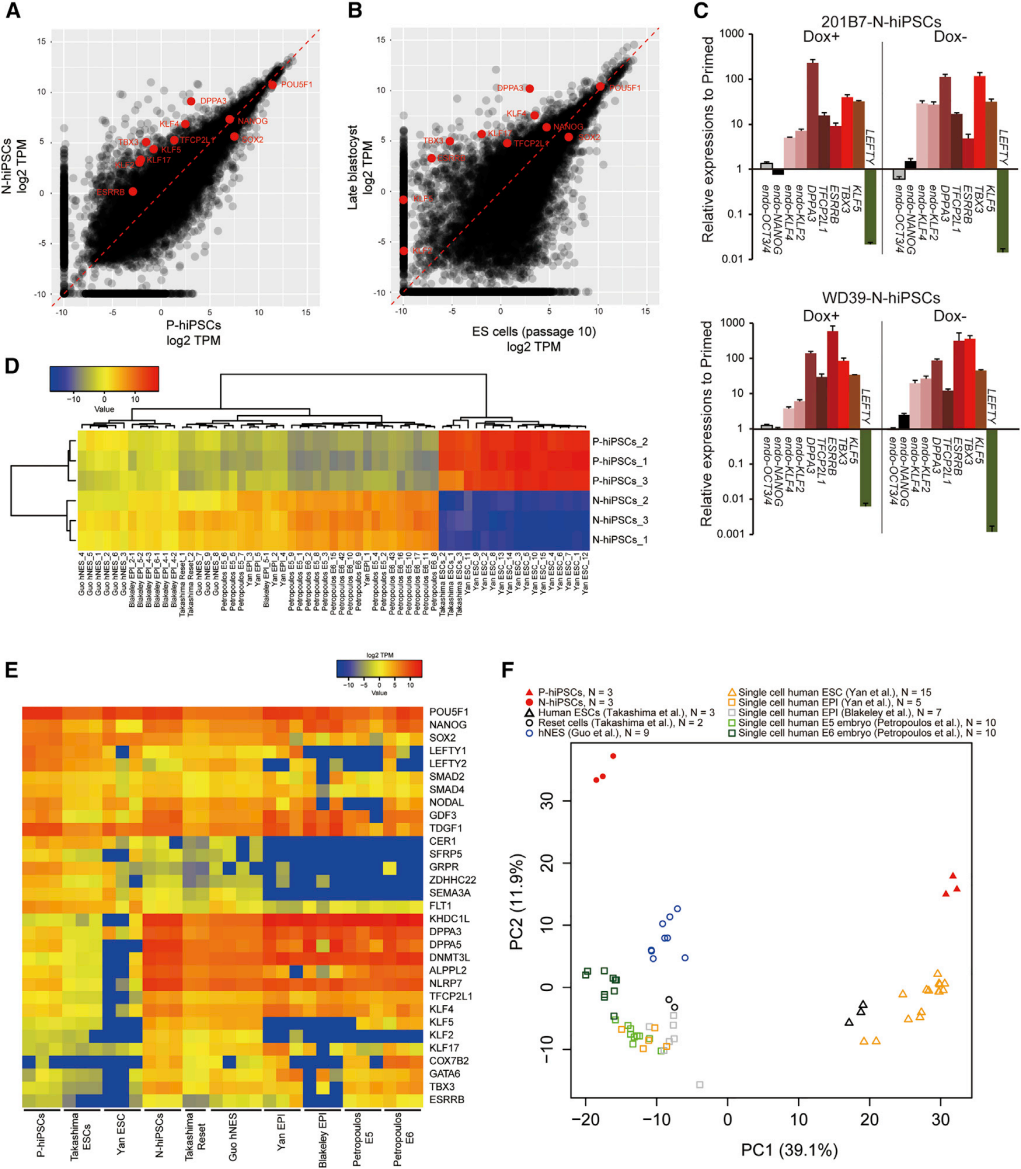
Gene Expression Analysis (A) Scatterplot of the P-hiPSCs versus the N-hiPSCs for the TPM values of each gene from RNA-seq data from this study. (B) Scatterplot of the hESCs versus the late blastocysts for the TPM values of each gene from the single-cell RNA-seq data from the study of Yan et al. (2013). (C) qPCR analyses of the gene expressions in the N-hiPSCs cultured in 2iLFA + Go (N2B27) before and after the withdrawal of Dox and in the primed counterparts. The reddish bars represent naive marker genes and the green bar represents a primed marker (n = 3; mean ± SEM; independent experiments). (D) Correlation matrix of the TPM values from the RNA-seq data from this study and the studies of Yan et al., 2013, Takashima et al., 2014, Blakeley et al., 2015, Petropoulos et al., 2016, and Guo et al. (2016). (E) Heatmap of the TPM values for selected genes from the RNA-seq data from this study with each dataset described above. (F) Principal component (PC) analysis of RNA-seq data from this study with each dataset described above.

We performed RNA sequencing (RNA-seq) analysis to characterize the global gene expression profile of the N-hiPSCs under the optimized culture conditions. For comparison, three independent cultures of N-hiPSCs in the absence of Dox and P-hiPSCs were analyzed, and significant differences were observed between these cells. Most of the core transcription factors in the ground-state self-renewal, including *KLF2*, *KLF4*, *TFCP2L1*, and *TBX3*, were upregulated in the N-hiPSCs (Figure 5A). In contrast, *SOX2* was slightly downregulated in the N-hiPSCs, although the expression levels of all of these genes were relatively high compared with those of other genes. The expression of *ESRRB*, which is an important factor in ground-state self-renewal, was upregulated in the N-hiPSCs that were generated using the method reported here, which contrasts with most previous reports of naive-like human pluripotent cells (Figures S3A–S3C). Moreover, *KLF17*, which was recently reported to be a reliable naive marker (Boroviak et al., 2015, Blakeley et al., 2015, Guo et al., 2016), was also upregulated in our N-hiPSCs. To verify our RNA-seq data, we performed qPCR analysis (Figure 5C) and found that the expressions of naive marker genes, including *ESRRB*, *DPPA3*, *KLF2*, *KLF4*, *KLF5*, *TFCP2L1*, and *TBX3*, were increased in the N-hiPSCs even after the withdrawal of Dox. Additionally we confirmed that the expressions of pluripotency markers were maintained at high levels and that the expression of a primed marker gene (*LEFTY*) was downregulated in the N-hiPSCs. Similar results were obtained from another hiPSC line, i.e., WD39-derived N-hiPSCs (Figure 5C). Finally, the whole-transcriptome profiles of these cells were compared with those of human blastocyst ICMs, reset cells, and human naive ESCs derived directly from ICMs (HNES) using published RNA-seq datasets (Yan et al., 2013, Takashima et al., 2014, Blakeley et al., 2015, Guo et al., 2016, Petropoulos et al., 2016). Our N-hiPSCs exhibited a transcriptome that was similar to those of the human blastocyst ICMs, reset cells, and HNES (Figures 5D and 5E). The expression pattern of the N-hiPSCs was quite similar to that of the human blastocyst ICMs with respect to the known core transcription factors in ground-state self-renewal (Figures 5A and 5B). Clustering by principal component analysis using the differently expressed genes with higher-fold changes (a log_2_ fold change of FPKM [fragments per kilobase of transcript per million mapped reads] > 3) revealed that the principal component 1 clearly discriminated the primed cells and naive cells (Figure 5F). Interestingly, focusing on each HNES sample separately revealed that some of the HNES exhibited moderate expression of *ESRRB* (Figure 5E), which supports the existence of a mESC-like naive pluripotent state in human in terms of *ESRRB* expression.

### Rapid and Efficient Neural Differentiation of Naive-like hiPSCs

**Figure 6:**
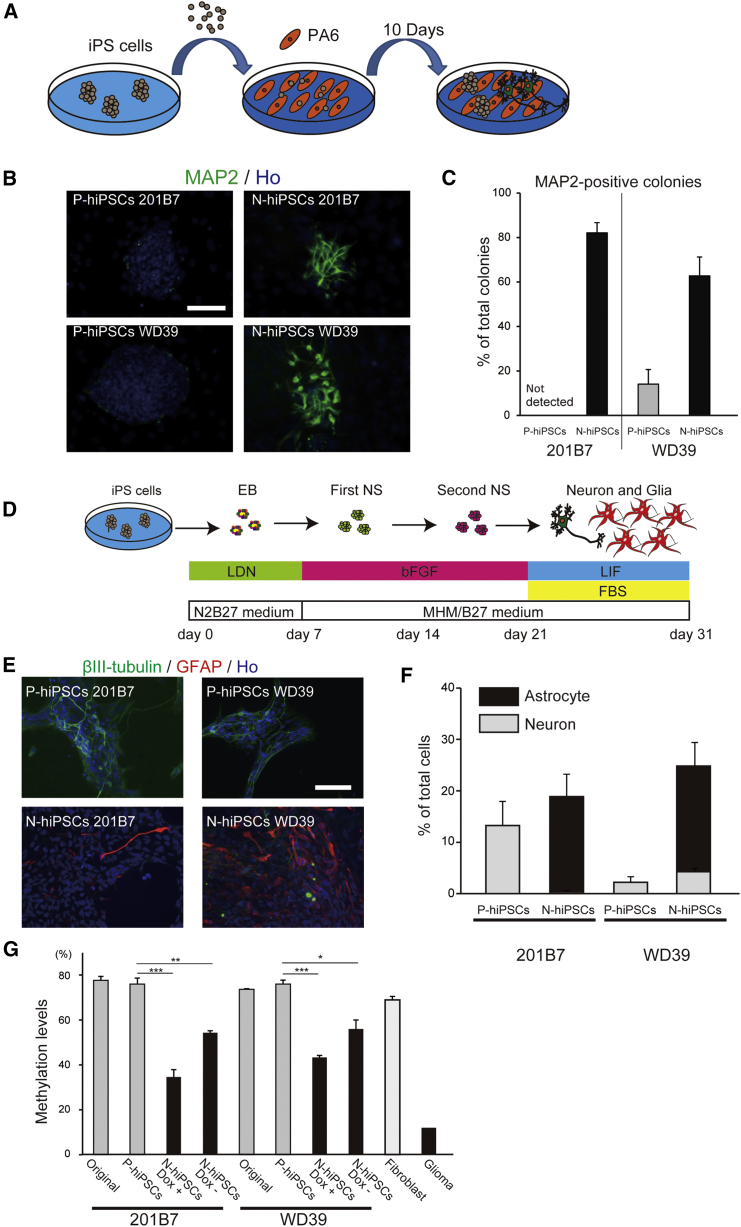
Neural Differentiation Capacities (A) Schematic presentation of the SDIA method. (B) Representative images of the immunocytochemistries of the differentiated iPSC colonies (neurons: MAP2). Scale bar, 100 μm. (C) Quantitative analysis of the percentages of colonies containing neurons (n = 3–5; mean ± SEM; independent experiments). (D) Schematic presentation of the protocols for the differentiation of astrocytes from hiPSCs. (E) Representative images of the immunocytochemistries of two groups of neural lineage cells (neurons: β-III tubulin; astrocytes: GFAP). Scale bar, 50 μm. (F) Quantitative analysis of the percentages of neurons and astrocytes in the differentiated neurospheres from the P-hiPSCs and N-hiPSCs (n = 3; mean ± SEM; independent experiments). (G) Frequency of STAT3 binding site methylation in the GFAP promoter as analyzed by bisulfite pyrosequencing (n = 3; mean ± SEM; independent experiments; ^∗^p < 0.05; ^∗∗^p < 0.01; ^∗∗∗^p < 0.001; t test). See also Figures S5 and S6.

To investigate the potential practical advantages of the naive-like hiPSCs in terms of differentiation, we compared the capacities for neural differentiation of the naive and primed hiPSCs using two differentiation methods. First, we applied the stromal cell-derived inducing activity (SDIA) method (Kawasaki et al., 2000) (Figure 6A). Because the SDIA method is a simple protocol whereby PSCs are simply co-cultured with PA6 stromal cells, it is well suited for assessing differentiation potential. We co-cultured P-hiPSCs and N-hiPSCs with PA6 cells, subjected the cells to immunocytochemical analysis for the neuronal marker MAP2 (Figure 6B) after 10 days in culture, and quantified the percentage of colonies that contained differentiated neurons. Surprisingly, the N-hiPSCs efficiently differentiated into neurons, whereas the P-hiPSCs rarely differentiated into neurons at this time point (Figure 6C). The P-hiPSCs required further cultivation for 10 days to give rise to neurons under the same conditions (Figure S5).

Next, we used a neurosphere-based method (Okada et al., 2008, Imaizumi et al., 2012) (Figure 6D) whereby primed and naive hiPSCs were differentiated into neural stem/progenitor cells as neurospheres through embryoid body formation. Secondary neurospheres were plated and allowed to differentiate for 10 days. The differentiated neurospheres were subjected to immunocytochemical analysis for βIII-tubulin (neurons) and glial fibrillary acidic protein (GFAP) (astrocytes) (Figure 6E). Unexpectedly, while the P-hiPSCs differentiated into βIII-tubulin-positive neurons, the N-hiPSCs predominantly differentiated into GFAP-positive astrocytes. These GFAP-positive astrocytes express other astrocyte markers (Figures S6A and S6B), and their activity was confirmed by calcium imaging (Figure S6C and Movie S1). As illustrated in Figure 6F, the percentages of GFAP-positive astrocytes were 18.5% and 20.5% in the cultures from the 201B7 N-hiPSCs and the WD39 N-hiPSCs, respectively, whereas no GFAP-positive astrocytes were differentiated from their primed counterparts at this stage (Figure S6A).

To explore the reason for this greater gliogenic competency of the N-hiPSCs, we checked the methylation status of the CpG dinucleotide at the STAT3 binding site in the GFAP promoter because the epigenetic gene regulation of GFAP at this site is important in the switch from neurogenesis to gliogenesis (Takizawa et al., 2001, Fan et al., 2005, Andoh-Noda et al., 2015). Both pyrosequencing and bisulfite sequencing analysis revealed a state of lower methylation in the N-hiPSCs than in the P-hiPSCs (Figures 6G and S6D).

In conclusion, the naive-like hiPSCs exhibit high neural differentiation potential compared with the conventional primed hiPSCs.

## Discussion

The existence of bona fide human naive pluripotent stem cells remains controversial, although various methods have been reported for maintaining hPSCs in states similar to those of mESCs (Hanna et al., 2010, Gafni et al., 2013, Chan et al., 2013, Takashima et al., 2014, Theunissen et al., 2014, Valamehr et al., 2014, Wang et al., 2014, Ware et al., 2014, Chen et al., 2015, Duggal et al., 2015, Hayashi et al., 2015, Qin et al., 2016). Naive iPSCs generated directly from somatic cells have also been reported (Yang et al., 2016).

The results of the present study indicate that the ectopic expression of a set of six transcription factors (i.e., OCT3/4, SOX2, KLF4, C-MYC, KLF2, and NANOG) strongly reprograms hiPSCs to a naive-like state that more resembles mESCs than naive-like cells that are converted using only KLF2 and NANOG. This finding indicates that the four Yamanaka factors synergistically act with KLF2 and NANOG to enable conversion from a primed to a naive state.

The reprogrammed cells exhibited characteristics that were highly similar to those of mESCs with respect to morphology, growth factor requirements, and global gene expression. In contrast to most previous reports, our naive-like hiPSCs highly expressed all of the known components of the core transcription factor network of naive mESCs, including *ESRRB*, and differentiated into both neurons and astrocytes more efficiently than their primed counterparts.

Previously reported naive-state hPSCs can be propagated in an LIF-dependent state in a manner akin to that of mESCs using a cocktail of small molecules (Gafni et al., 2013, Chan et al., 2013, Theunissen et al., 2014, Valamehr et al., 2014, Ware et al., 2014, Duggal et al., 2015). However, similar to mEpiSCs and conventional hiPSCs/hESCs, these cells still depend on TGF-β, FGF-2, or both. These findings are suggestive of insufficient reprogramming, resulting in converted cells that are in an intermediate state between naive and primed. In contrast, hPSCs that are reprogrammed with C6F can be maintained without TGF-β or FGF-2. Our results are in agreement with the report by Takashima's group in which the short-term expression of NANOG and KLF2 transgenes (T2F) was used to reprogram human cells to be independent of TGF-β and FGF-2 (Takashima et al., 2014). These results led us to conjecture that the expression of an appropriate set of transcription factors would be able to more robustly convert human cells to a naive state than a change in medium conditions. Meanwhile, when applying this method in the field of regenerative medicine, the genomic integration of the transgenes will be problematic. Developing a method that is integration-free or not having to rely on transgenes will be essential in the future.

Culture conditions can also be important in the conversion and maintenance of altered pluripotent states. In this study, we used 2iL medium plus a PKC inhibitor, forskolin, and a TGF-β inhibitor for conversion and maintenance because some reports have suggested that a PKC inhibitor and forskolin facilitate conversion from the primed to the naive state (Hanna et al., 2010, Dutta et al., 2011, Takashima et al., 2014, Duggal et al., 2015, Qin et al., 2016). Additionally, TGF-β inhibitors may facilitate the selection of naive-like cells because primed cells cannot be propagated in medium containing a TGF-β inhibitor. Whether the TGF-β/Activin signaling is necessary for naive hPSCs is still a matter of discussion. In experiments using human blastocysts, inhibiting the TGF-β/Activin signal has led to contradictory results. In one report, the signal inhibition promoted epiblast formation (Van der Jeught et al., 2014) while in another, epiblast formation was inhibited (Blakeley et al., 2015). Furthermore, in experiments using non-human primate embryos, epiblast formation was neither promoted nor inhibited when TGF-β/Activin signaling was inhibited (Boroviak et al., 2015). The present results from our experiments suggest that the signal inhibition is not essential once the cells are converted into a naive-like state.

The transcription factor circuitries that govern pluripotency differ between naive and primed cells. For example, *Esrrb*, *Nanog*, *Klf2*, *Klf4*, and *Tfcp2l1* are highly expressed and play important roles in mESCs, whereas these genes are expressed at low levels in mEpiSCs and hPSCs. Although some of these genes can be elevated in human cells by naive-like conversion as described in previous reports, in most cases the expression of *ESRRB* is unaffected (Hanna et al., 2010, Gafni et al., 2013, Chan et al., 2013, Theunissen et al., 2014, Valamehr et al., 2014, Ware et al., 2014). In contrast, the naive-like hiPSCs in the present study maintained ESRRB expression at high levels even after transgene expression was suppressed. However, it remains controversial whether ESRRB is highly expressed in the bona fide human naive pluripotent cells. Blakeley et al. (2015) recently reported that some naive markers upregulated in rodents, including *KLF2* and *ESRRB*, are rarely expressed in the human epiblast. However, in our transcriptome analysis including datasets from previous reports, the epiblast datasets from Petropoulos et al. (2016) showed *ESRRB* expression. Since the blastocyst development is a dynamic process, differences in the blastocyst stage used in the analysis may have caused this discrepancy. On the other hand there is no *KLF2* expression in any epiblast dataset, whereas its expression is observed in naive-type hPSCs expanded *in vitro*, including reset cells and NHES. Our results suggest that *ESRRB* and *KLF2* expression may not be critical for epiblast development but may play a role in the self-renewal of PSCs *in vitro*. Indeed, the *KLF2* knockout mouse survived until embryonic day 11.5–13.5 without any early developmental abnormality (Wani et al., 1998). Meanwhile, the *ESRRB* knockout mouse could not survive after embryonic day 10.5, but its death was caused by abnormalities in the placenta, which is not derived from the epiblast (Luo et al., 1997). Yet in *in vitro* culture, both genes play an important role for the self-renewal of naive-state mESCs (Festuccia et al., 2012, Martello et al., 2012, Yeo et al., 2014, Qiu et al., 2015). Analysis of our naive-like cells suggests that these genes may have some specific function in the *in vitro* expansion of human naive-state PSCs as well. Further studies are needed to explore the role of these genes in the self-renewal of naive human PSCs.

Little information is available regarding the differentiation potential of human naive cells. We obtained results suggesting that N-hiPSCs have a higher differentiation potential into the three germ layers through embryoid body formation. Furthermore, we performed neural differentiation assays and found that human naive-like cells differentiate more rapidly into neurons and astrocytes than do their primed counterparts. These results support the perspective that naive conversion is conducive to neural differentiation (Honda et al., 2013).

In addition, the human naive-like cells were also able to give rise rapidly to astrocytes. The length of developmental period of the animal species, from which the PSCs were derived, may be one factor that can affect the differentiation speed of the PSCs. This could possibly be the reason why mPSCs can differentiate into glial cells faster than human PSCs. Our data suggest that the starting pluripotent state can also affect the speed of glial differentiation *in vitro*. Although the detailed mechanism of the rapid glial differentiation of naive-like hiPSCs remains unclear, we found that the GFAP promoter region, i.e., the important locus for glial differentiation, is hypomethylated in naive-like hiPSCs compared with their primed counterparts.

Some reports have demonstrated that human naive cells exhibit genome-wide DNA hypomethylation (Gafni et al., 2013, Takashima et al., 2014), which is a feature of mESCs in 2iL medium and early embryonic epiblasts (Leitch et al., 2013, Habibi et al., 2013, Ficz et al., 2013, Guo et al., 2014). This hypomethylated genomic state may cause an increase in the sensitivity to differentiation signals. Additionally, a hypomethylation status may be beneficial to the maturation of hiPSC-derived differentiated cells because the maturation capacity of hiPSC-derived hematopoietic precursors has been reported to be related to the amount and pattern of DNA methylation acquired during reprogramming in hiPSCs (Nishizawa et al., 2016). Further investigations are needed to clarify the relations of methylation status and differentiation propensities.

Generally, primed pluripotency has been described as a priming phase for differentiation into various cell lineages. Notably, the propensity for differentiation among clones is diverse (Osafune et al., 2008, Liang and Zhang, 2013). In contrast, because the naive pluripotent state has no biased differentiation propensity (Nichols and Smith, 2009), naive-like hiPSCs are thought to be more responsive to extrinsic differentiation cues. This may be one factor underlying the rapid neural differentiation exhibited by naive-like hiPSCs. Findings from studies of mouse primed cells support this possibility because different mEpiSC lines exhibit distinct gene expression patterns, and some mEpiSC lines exhibit resistance to differentiation into neural lineages (Bernemann et al., 2011). Consistent with these findings, Song et al. (2016) report that, even in the cell line, mEpiSCs exhibit heterogeneity and biased differentiation potential. Moreover, Jang et al. (2014) reported that the global gene expression pattern of brain-derived NSCs is more similar to that of mESC-derived NSCs than that of mEpiSC-derived NSCs. Naive-like hiPSCs may thus be advantageous for maximizing the quality of hiPSC-derived differentiated cells.

In conclusion, this study provides a modified method for reprogramming human primed cells to a naive state in which all known naive pluripotency-related transcription factors, including *ESRRB*, are expressed. These naive-like hiPSCs exhibit efficient neural differentiation and rapid glial differentiation compared with conventional primed hiPSCs. These features of naive-like hiPSCs may facilitate disease modeling and regenerative medicine research using hPSCs because some types of cells that are difficult and/or time-consuming to obtain from conventional hiPSCs can be easily differentiated from naive-like hiPSCs.

## Experimental Procedures

See also Supplemental Experimental Procedures.

### Cell Culture

The hiPSC lines 201B7 and WD39 (Imaizumi et al., 2012) were used in this study. For the conventional primed condition culture, the hiPSCs were grown on mitomycin-C-treated SNL murine fibroblast feeder cells or irradiated mouse embryonic fibroblasts in 0.1% gelatin-coated tissue culture dishes. The hiPSCs were maintained in standard hESC medium (DMEM/F12 [Wako] containing 20% KSR [Life Technologies], 2 mM L-glutamine [Nacalai Tesque], 1% non-essential amino acids [Sigma], 0.1 mM 2-mercaptoethanol [Sigma], and 4 ng/mL FGF-2 [PeproTech]) at 37°C in atmospheric oxygen (20% O_2_, 3% CO_2_). The hiPSCs were subcultured with a dissociation solution (0.25% trypsin, 100 μg/mL collagenase IV [Invitrogen], 1 mM CaCl_2_, and 20% KSR) every 5–7 days.

## Author Contributions

F.K., S. Shiozawa, and H.O. conceived and designed the experiments. F.K., K.O., S.Y., I.K., K.K., and S. Suzuki performed the experiments and analyzed data. F.K., S. Shiozawa, K.O., M.N., and H.O. wrote and edited the manuscript. All authors read and approved the final manuscript.

## Supplemental Information

Document S1. Supplemental Experimental Procedures, Figures S1–S6, and Table S1

Movie S1. Calcium Imaging in the N-hiPSC-Derived Astrocytes

Document S2. Article plus Supplemental Information
