# Supplementary material for: Naive-like ESRRB+ iPSCs with the Capacity for Rapid Neural Differentiation
Source: Stem Cell Reports. 2017 Nov 9;9(6):1825–38. doi: 10.1016/j.stemcr.2017.10.008 (PMC5785673; doi:10.1016/j.stemcr.2017.10.008)
Supplement: Document S1. Supplemental Experimental Procedures, Figures S1–S6, and Table S1 [file mmc1.pdf]

Stem Cell Reports, Volume *9*

Supplemental Information

Naive-like ESRRB$^+$ iPSCs with the Capacity for Rapid Neural Differentiation

Fumihiko Kisa, Seiji Shiozawa, Keisuke Oda, Sho Yoshimatsu, Mari Nakamura, Ikuko Koya, Kenji Kawai, Sadafumi Suzuki, and Hideyuki Okano

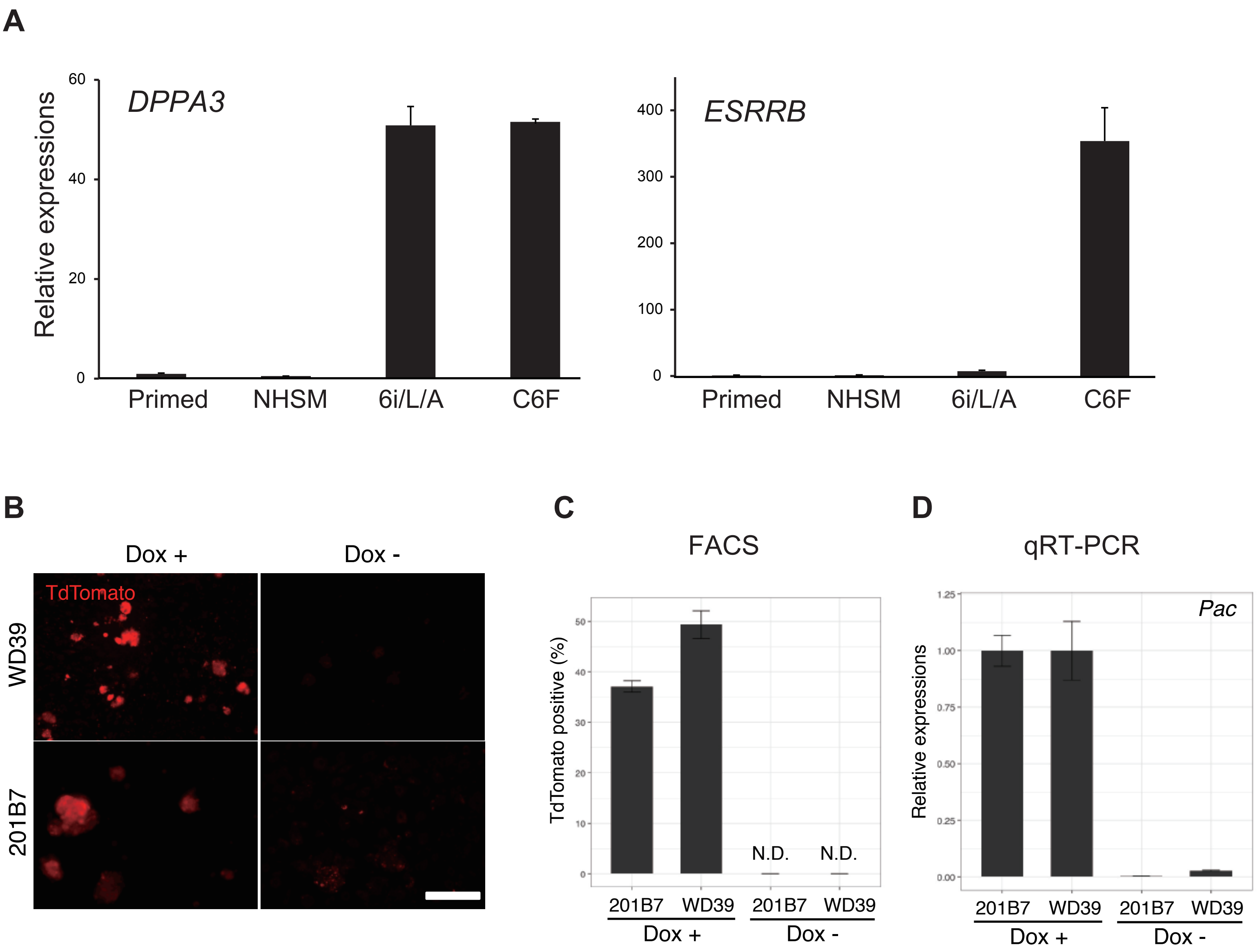

Figure S1. Comparison of C6F method with transgene-free naïve-conversion method and regulation of the C6F transgene expression by Doxycycline. (related to Figure 1 and Figure 3)

(A) qPCR analysis of the expressions of naïve marker genes (*DPPA3* and *ESRRB*) in WD39 cells expressing C6F or maintained in naïve-conversion medium as previously reported (n = 3; mean ± SEM; independent experiments). (B) Transgene expression indicator TdTomato disappeared after removal of doxycycline(Dox). The scale bar represents 100 µm. (C) Flowcytometric analysis of the TdTomato expression (n = 3; mean ± SEM; independent experiments). (D) Quantitative RT-PCR of *puromycin N-acetyl-transferase* (*Pac*) gene before and after Dox removal in N-hiPSCs (n = 3; mean ± SEM; independent experiments).

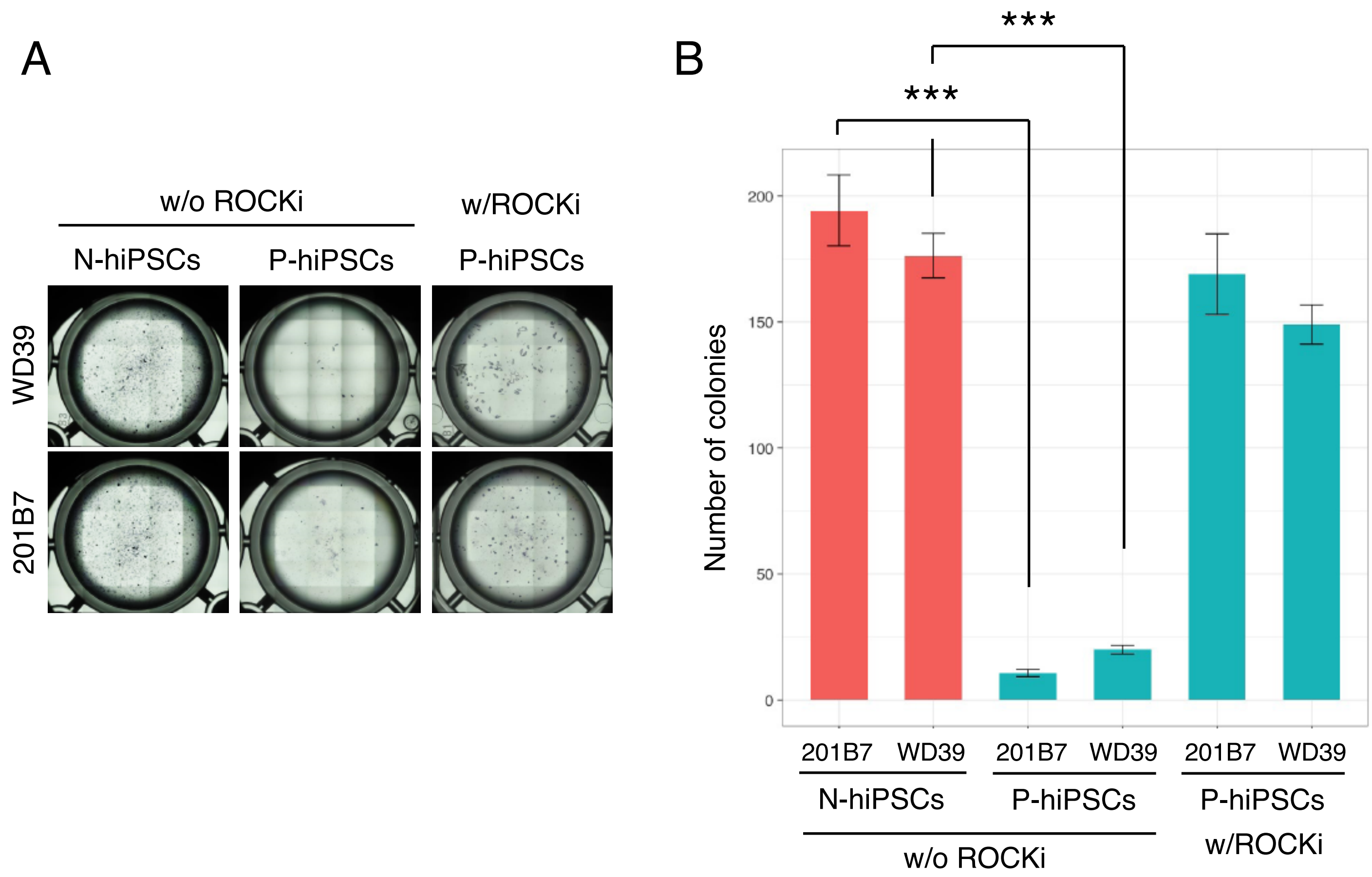

Figure S2. Colony formation assay after single cell dissociation (related to Figure 2)

(A) The number of alkaline phosphatase-positive colonies after single cell dissociation. N-hiPSCs can form colonies without ROCK inhibitor treatment. (B) Quantification of the number of alkaline phosphatase-positive colonies. ($n = 3$; mean ± SEM; independent experiments; ***$P < 0.001$; t test)

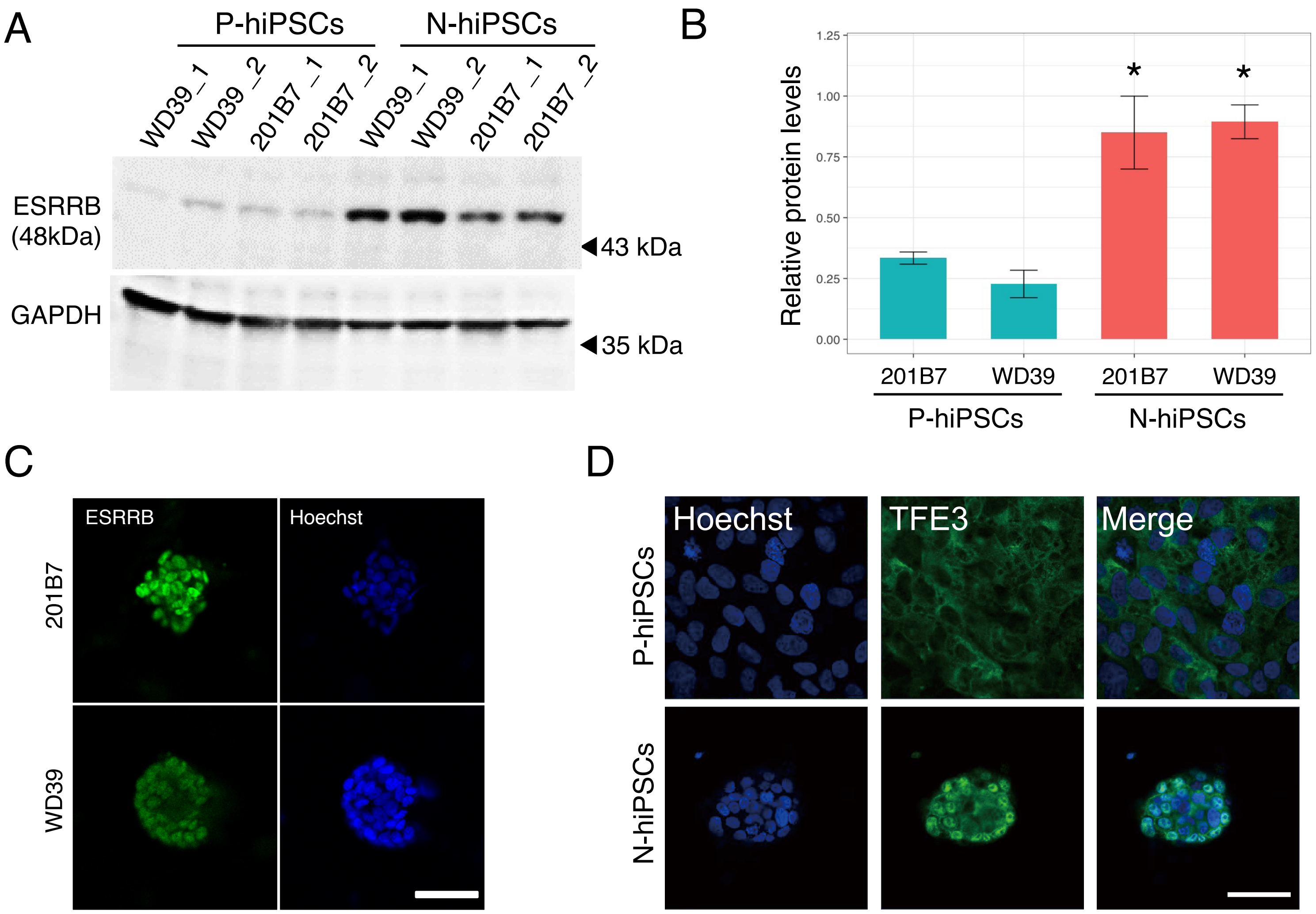

Figure S3. ESRRB protein expression, TFE3 subcellular localization in the N-hiPSCs (related to Figures 2 and 5)

(A) Western blot analysis of ESRRB in the P- and N-hiPSCs. The result of 2 independent samples from each line is presented. (B) Quantification of the GAPDH-normalized ESRRB protein levels from the western blots. Bars indicate the average of the 3 independent samples and error bars indicate SEM. *P < 0.05 (C) ESRRB immunocytochemistry in the N-hiPSCs. The scale bar represents 50 μm. (D) Immunocytochemical analyses of the 201B7-derived P-hiPSCs and N-hiPSCs for TFE3. The scale bar represents 50 μm.

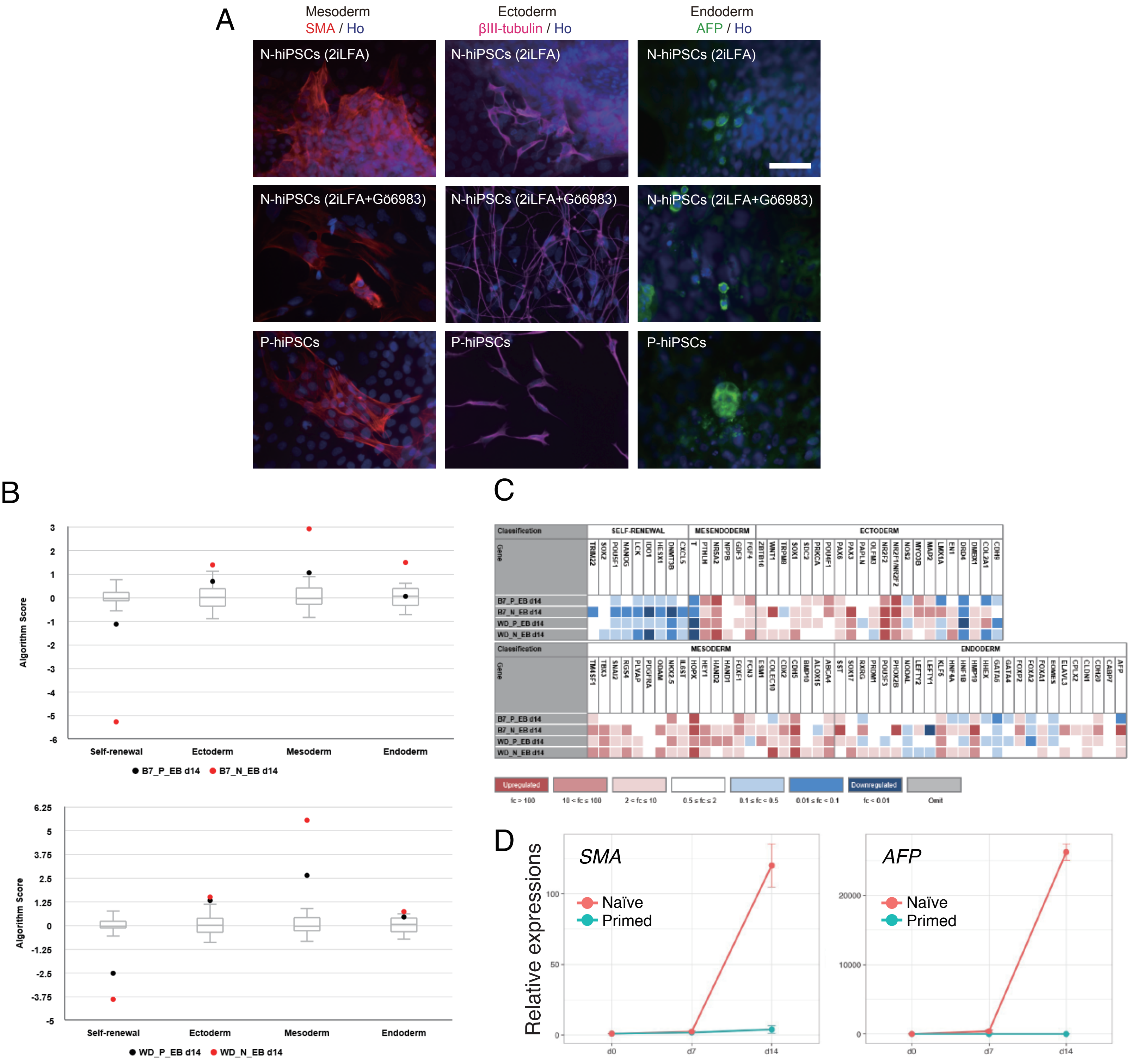

Figure S4. Three germ layer differentiation potential (related to Figures 2 and 3)

(A) Immunocytochemistries of differentiated EBs from 201B7 N-hiPSCs cultured in 2iLFA (upper) or 2iLFA+Go (N2B27) (middle), and their primed counterparts (lower) for smooth muscle actin (SMA), β-III-tubulin and α-fetoprotein (AFP). The scale bar represents 50 µm. (B, C) Quantitative analysis of the trilineage differentiation potential using the TaqMan hPSC scorecard kit. (B) Comparison of the differentiation potentials between N- (red) and P- (black) hiPSCs derived from 201B7 (top) and WD39 (bottom), respectively. The grey box plot indicates the reference data set provided by the manufacturer. The error bars represent the maximum and minimum values of the reference data set. (C) Heat map of each gene expression level relative to the reference. (D) qPCR analysis of gene expression time course in EBs derived from P- and N-hiPSCs (days 0, 7 and 14). (n = 3; mean ± SEM; independent experiments)

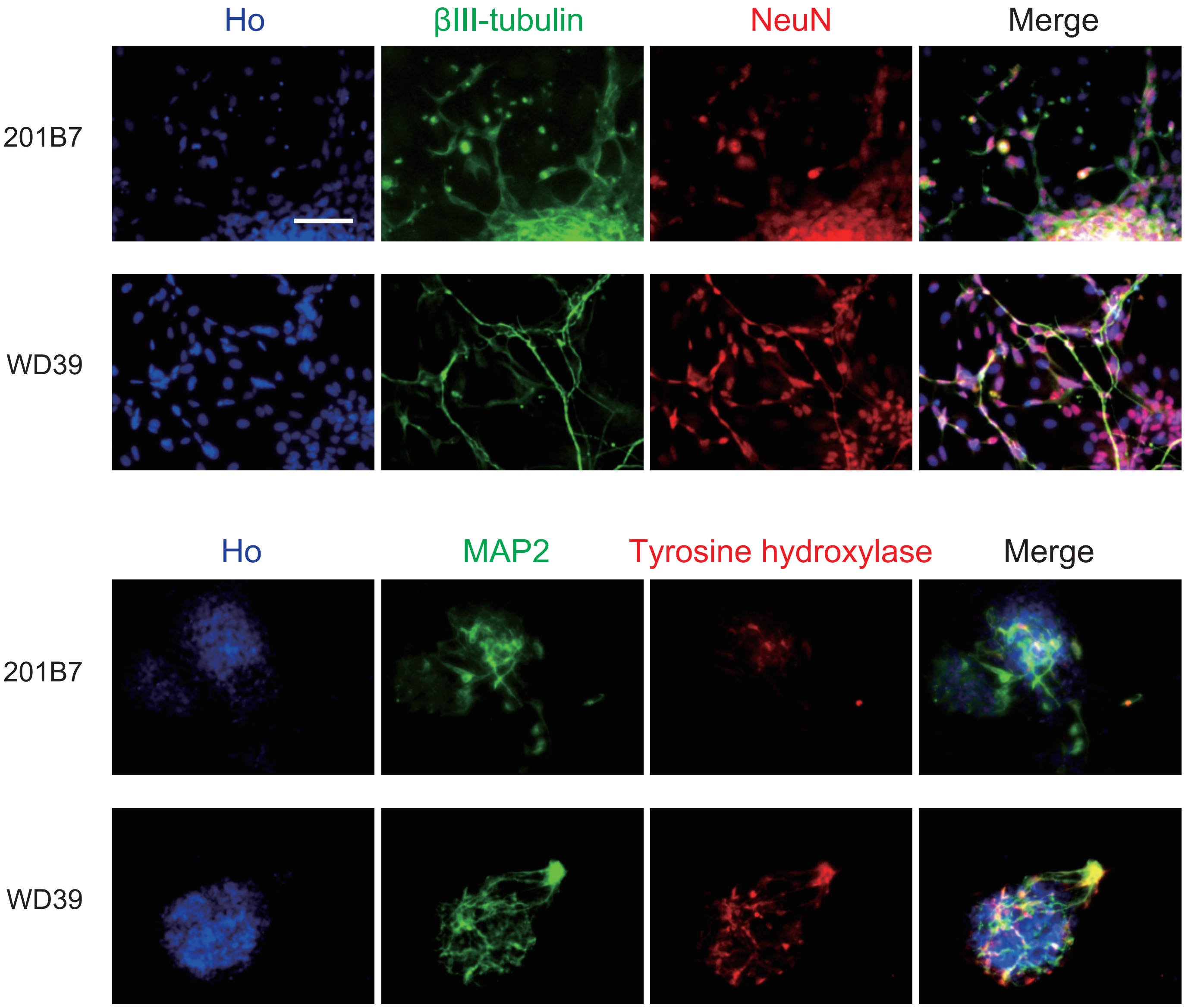

Figure S5. Neuronal differentiation of the primed hiPSCs by the SDIA method over 20 days (related to Figure 6)
Representative immunocytochemical images of SDIA-differentiated colonies using pan-neuronal markers (β-III-tubulin, MAP2, NeuN) and a marker for dopaminergic neurons (tyrosine hydroxylase). The scale bar represents 50 μm.

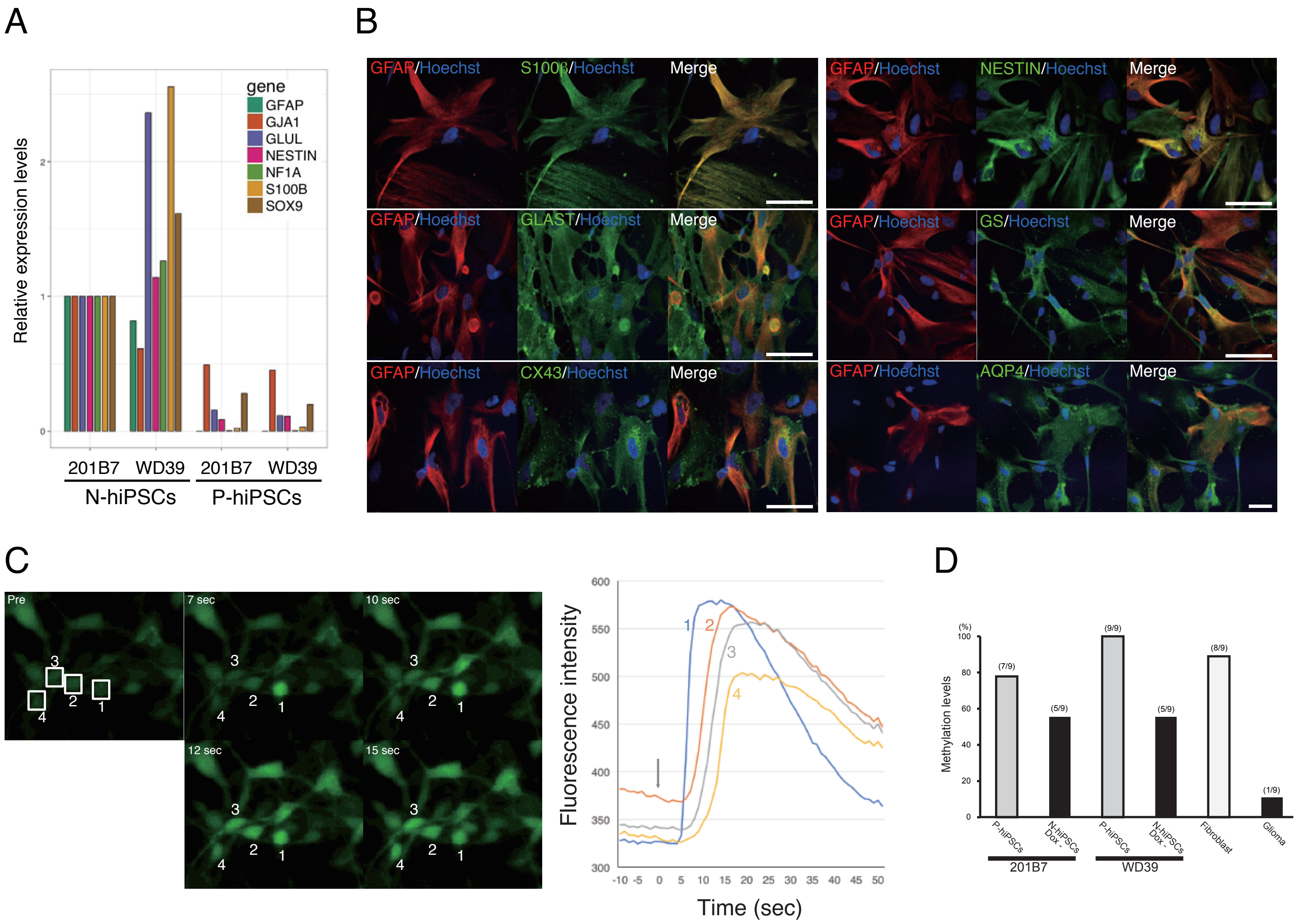

Figure S6. Glial differentiation (related to Figure 6)

(A) qPCR analysis of astrocytic marker expression in the differentiated cells derived from N- and P-hiPSCs using a neurosphere-based method. Bars indicate the average of the technical tripricate. (B) Immunocytochemistry of N-hiPSCs-derived astrocytes for astrocytic markers. The scale bar represents 50 µm. (C) Calcium imaging in the N-hiPSCs-derived astrocytes using a calcium indicator Fluo-4. The left panel shows representative time course images. The right graph indicates changes of fluorescence intensity after ATP administration. The white boxes in the picture from the left panel indicate the quantified cells, whose numbers correspond to the numbers in the right graph. See also Movie S1. Similar results were obtained from 3 independent experiments. (D) Hypomethylation of the STAT3 binding site in the GFAP promoter of the N-hiPSCs. Frequencies of STAT3 binding site methylation in the GFAP promoter as analyzed with cloning approaches.

Table S1. Primer sequences

Gene	Forward	Reverse
endo-NANOG	gacactggctgaatccttcctct	accctccatgagattgactggat
endo-OCT3/4	agtttgtgccagggtttttg	acttcaccttccctccaacc
endo-KLF4	gccagaaagcactacaatcatgg	ttggcattttgtaagtccaggaa
endo-KLF2	gtgggaaaagaccacgatcctcc	tctcacaaggcatcacaagcctc
LEFTY	agctgcacaccctggacctt	gtcattggtgcttcagggtca
DPPA3	HA216940 [Takara perfect real time]	HA216940 [Takara perfect real time]
ESRRB	HA095808 [Takara perfect real time]	HA095808 [Takara perfect real time]
TFCP2L1	HA193431 [Takara perfect real time]	HA193431 [Takara perfect real time]
KLF5	HA224420 [Takara perfect real time]	HA224420 [Takara perfect real time]
TBX3	HA142445 [Takara perfect real time]	HA142445 [Takara perfect real time]
SOCS3	gtgcgccatggtcacccaca	gcttgcgcactgcgttcacc
ACTB	tgaagtgtgacgtggacatc	ggaggagcaatgatcttgat
SMA	gacaatggctctgggctctgtaa	tgtgttcgtcacccacgta
AFP	gtagcgctgcaaacgatgaa	tccaacaggcctgagaaatc
Pac	gaccgagtacaagcccacgg	acgcgcgtgaggaagagttc
GFAP	acatcgagatcgccacctac	cggagcaactatcctgcttc
NESTIN	tggaggcaaagagggttcag	tcggagaactctgtcccca
NF1A	agctcatggagcggcaatag	attcatcctgggtgagacagag
GJA1	HA151076 [Takara perfect real time]	HA151076 [Takara perfect real time]
GLUL	HA279385 [Takara perfect real time]	HA279385 [Takara perfect real time]
S100B	HA168460 [Takara perfect real time]	HA168460 [Takara perfect real time]
SOX9	HA148317 [Takara perfect real time]	HA148317 [Takara perfect real time]

Supplemental Experimental Procedures

Introduction of transgenes

To evaluate the effects of transcription factors on naïve conversion, pPB-T2F/Venus (Dox-inducible transgene carrying NANOG, KLF2 and Venus), pPB-Y4F/Cerulean (Dox-inducible transgene carrying the four Yamanaka factors and Cerulean), pPB-C6F/TdTomato (Dox-inducible transgene carrying the four Yamanaka factors, NANOG, KLF2 and TdTomato) and pPB-Venus (control mock vector) were introduced into hiPSCs with reverse tetracycline transactivator (rtTA) and piggyBac transposase expression vectors using GeneJuice Transfection Reagent [Novagen]. The transfected cells were cultured in primed condition medium containing 1 µg/mL Dox; furthermore, 100 µg/mL G418 (pPB-T2F/Venus) or 1 µg/mL puromycin (pPB-Venus, pPB-Y4F/Cerulean and pPB-C6F/TdTomato) were added for selection. The selected cells were subjected to quantitative PCR (qPCR) analysis after seven days.

For cloning, the transfected cells were selected by adding 100 µg/mL hygromycin for five to seven days. The Dox-inducible expression systems in the cells were verified based on the Dox-inducible TdTomato expression after a short-term addition of Dox. Next, suitable colonies were picked up with a pipette and transferred to a new culture. The cells were expanded in the primed condition.

Naïve conversion

For conversion to the naïve pluripotent state, the C6F transgenic hiPSC colonies were detached from the feeder layers using the dissociation solution and were dissociated into single cells using TrypLE Select [Life Technologies]. The cells were seeded on irradiated mouse embryonic fibroblasts in two different mediums (N2B27 medium: 1:1 mixture of Neurobasal [Life Technologies] and DMEM/F-12, 1% N2 supplement [Life Technologies], 2% B27 supplement [Life Technologies], 1 mM L-glutamine, 1% NEAA, 0.1 mM 2-ME, and 50 µg/mL AlbuMax I [Life Technologies]; KSR medium: KnockOut DMEM [Life Technologies], 20% KSR, 2 mM L-glutamine, 1% NEAA, and 0.2 mM 2-ME) both of which contained 1 µg/mL Dox, 10 ng/mL human LIF [Nacalai tesque], 1 µM CHIR99021 [Axon Medchem], 1 µM PD0325901 [Wako], 10 µM Forskolin [Sigma], and 5 µM A83-01 [Santacruz]. For the transgene independent culture, 5 µM Gö6983 [R&D Systems] was included to enhance conversion toward the naïve pluripotency state and to maintain naïve pluripotency without Dox. Ten micromolars of Y27632 [Sigma] was supplemented for the first 24 hrs of the primed-to-naïve conversion. The converted cells were grown at 37°C under hypoxic conditions (5% O_2, 5% CO_2). The cells were

subcultured using trypsin/EDTA [Invitrogen] every five to six days without Y27632. The Dox could be removed 10 days after the start of the naïve conversion. Additionally, 0.6 μM JAK1 inhibitor [Calbiochem] was used to assess the LIF/STAT3 signal dependency.

Naïve conversion via the medium conditions

hiPSCs were cultured in NHSM (Knockout DMEM containing 1% N2 supplement, 1 mM L-glutamine, 1% NEAA, 0.1 mM 2-ME, 10 mg/mL AlbuMax I, 12.5 μg/mL recombinant human insulin [Wako], 20 ng/mL human LIF, 1 ng/mL TGF-β, 8 ng/mL FGF-2, 3 μM CHIR99021, 1 μM PD0325901, 5 μM Y27632, 10 μM SB203580 [InvivoGen], 10 μM SP600125 [Enzo Life Sciences] and 10 μM Gö6983 [Tocris]) (Gafni et al., 2013) or 6i/L/A (N2B27 medium containing 20 ng/mL human LIF, 1 μM PD0325901, 1 μM IM-12 [Enzo Life Sciences], 0.5 μM SB590885 [R&D Systems], 1 μM WH-4-023 [A Chemtek], 10 μM Y27632, 20 ng/mL Activin A [PeproTech], 0.5% KSR and 8 ng/mL FGF-2) (Theunissen et al., 2014).

Reverse transcription and quantitative PCR analysis

Total RNA was isolated using an RNeasy Mini Kit [QIAGEN] and reverse transcribed using ReverTra Ace [TOYOBO]. The qPCR analysis was performed with the SYBR Green Master Mix [Life Technologies] on a ViiA7 real-time PCR platform [Applied Biosystems] according to the manufacturer' s instruction. The data are presented as the relative mRNA expression levels normalized by *ACTB*. The RT-PCR primers used herein are provided in Table S1.

RNA-seq

Total RNA was extracted using an RNeasy Mini Kit. The qualities and quantities of the RNA preparations were assessed using a 2100 Bioanalyzer with an RNA 6000 Nano LabChip Kit [Agilent Technologies]. Poly(A)+ RNA was selected and converted to a library of cDNA fragments (200–250 bp) with adaptors attached to both ends for sequencing using a TruSeq Stranded mRNA LT Sample Prep Kit Set v2 [Illumina] as per the manufacturer's instructions. The libraries were quantified using a Bioanalyzer DNA High Sensitivity Kit [Agilent Technologies] and a Kapa Library Quantification Kit [Kapa Biosystems] using an Applied Biosystems StepOne Real-Time PCR System according to the manufacturer's instructions. The libraries were then loaded into a flow cell for cluster generation using the TruSeq Rapid SR Cluster Kit [Illumina] and sequenced using an Illumina HiSeq2500 to obtain 51-nucleotide sequences (single-end). Sequencing reads from published RNA-seq experiments were obtained from the GEO

(GSE36552) or the ArrayExpress (E-MTAB-2857, E-MTAB-3929 and E-MTAB-4461) databases. For comparison with our RNA-seq data, raw counts of the published RNA-seq experiments and our experiment were processed as follows. Poor-quality reads (scores < 20) and adapter sequences were trimmed using the Trimmomatic [Bolger et al. 2014]. The remaining reads were cut to 43 bases and shorter reads (length < 30) were removed. The resulting reads were mapped to the UCSC human genome 19 using sailfish version 0.7.6 and the reads mapped in rRNA were omitted. The quantities of the transcripts were measured in templates per million (TPM). ExAtlas (https://lgsun.irp.nia.nih.gov/exatlas/) was used to analyze global correlations between gene expression data. The principal components were computed with the princomp function of the R package. R packages were used to construct the gene expression plots, perform the principal component analyses and create the heatmaps. RNA-seq data has been registered in the GEO:GSE104583.

Bisulfite sequencing

Genomic DNAs from BJ human fibroblasts [ATCC], the U-87 human glioma cell line [ATCC], P-hiPSCs, N-hiPSCs and the original hiPSC clones (201B7 and WD39) were prepared using DNeasy Blood and Tissue Kits [QIAGEN]. One to two micrograms of genomic DNA was modified by treatment with sodium bisulfite using an EpiTect Bisulfite kit [QIAGEN]. A region in the GFAP promoter containing the STAT3 binding site was then amplified by PCR from the bisulfite-treated genomic DNA. The PCR products were analyzed with pyrosequencing and cloning approaches. For the pyrosequencing approach, PCR was performed with the PyroMark PCR kit [QIAGEN] using the following forward and reverse primers, which were designed using PyroMark Assay Design Software [QIAGEN]: hGFAP promoter-forward (5’ -GGGTTTTTTTTTATGTTTAGTGAATGAT-3’) and biotin-conjugated hGFAP promoter-reverse (5’ -ATCCCAAAATACCAAACTATCAAAC-3’). Pyrosequencing was performed with hGFAP promoter-sequence primer (5’ -CGTATTTTAGTTTTT-3’) on the PyroMark Q24 platform [QIAGEN] according to the manufacturer’ s instructions. For the cloning approach, the PCR products from hGFAP promoter-forward primer 2 (5’ -TTGGGGAGGAGGTAGATAGTTAGGTTTT-3’) and hGFAP promoter- reverse primer 2

(5’ -CATCCCCTAATCCCCTTTCCTAAA-3’) were cloned into a pCR™-Blunt II-TOPO® vector [Invitrogen], and 9 clones from each sample that were randomly selected were sequenced.

Immunocytochemical analysis

The cells were fixed with 4% paraformaldehyde (PFA) for 15–30 min at room temperature. After incubation with blocking buffer (PBS containing 5% fetal bovine serum (FBS) and 0.3% Triton X-100) for 30–60 min at room temperature (RT), the cells were incubated with primary antibodies at 4°C overnight. For the ESRRB and TFE3 staining, we used commercially available blocking buffer [Thermo Scientific]. For the TFCP2L1 staining, we used PBS-based blocking buffer containing 10% goat serum and 0.05% Tween 20. After the incubation with the primary antibodies, the cells were washed with PBS (-) three times and were then incubated with Alexa 488-, Alexa 555-, or Alexa 647-conjugated secondary antibodies [Life Technologies] for 1 hr at RT. Additionally, the nuclei were stained with 10 mg/mL Hoechst 33258 [Sigma]. After three washes with PBS(-), the cells were mounted on slides and examined with a universal fluorescence microscope [Axiophoto; Carl Zeiss] or a confocal laser scanning microscope [LSM700; Carl Zeiss]. The primary antibodies used in these analyses were as follows: β-III-tubulin (1:1,000; T8660, Sigma), GFAP (1:500; 13-0300, Thermo Fisher Scientific), MAP2 (1:1,000; M4403,Sigma), S100B (1:500; S2532, Sigma), Nestin (1:500; described previously (Kanemura et al., 2002; Nakamura et al., 2003)), AQP4 (1:200; sc-20812, Santacruz), Glutamine synthase (1:500; 610518, BD Biosciences), Cx43 (1:500; ab11370, abcam), GLAST (1:10; 130-095-814, Miltenyi Biotec), ESRRB (1:2000; PP-H6705, Perseus Proteomics Inc.), TFE3 (1:100; HPA023881, Sigma), TFCP2L1 (1:400; ab123354, Abcam), α-fetoprotein (AFP; 1:250; MAB1368, R&D Systems), smooth muscle actin (SMA; 1:150; A2547, Sigma), NeuN (1:100; MAB377, Millipore), and tyrosine hydroxylase (1:500; AB152, Millipore).

Western Blot Analysis

Protein was extracted from P- and N-hiPSCs using RIPA buffer, supplemented with a protease inhibitor cocktail [Roche]. 10μg of total protein were separated by a 7.5-15% SDS-polyacrylamide (PAGE) gel and transferred to a PVDF membrane. After blocking, the membrane was incubated with an anti-ESRRB antibody. Immunoreactive bands were hybridized with an anti-mouse horseradish and visualized with the ECL™ Prime Western Blotting Detection Reagent. Images were acquired with LAS-4000.

Colony formation assays

P-iPSCs and N-iPSCs from the 201B7 and WD39 lines were dissociated into single cells with trypsin-EDTA. $1x10^5$ cells were seeded on triplicate wells with MEF feeder cells, and cultured either with or without ROCK inhibitor treatment. About 7 days after

plating, the cells were fixed and stained for Alkaline Phosphatase using the sigmaFAST BCIP/NBT kit [Sigma]. The number of colonies positive for the staining was counted manually.

Embryonic body (EB) formation

N-hiPSC and P-hiPSC colonies were detached from the feeder layers en bloc using the dissociation solution and by pipetting, respectively. For EB formation, the clusters of undifferentiated iPSCs were incubated for two weeks in DMEM containing 15% FBS, 2 mM L-glutamine, 1% NEAA, and 0.1 mM 2-ME by suspension culture. For further differentiation, the EBs were plated on poly-L-ornithine/fibronectin-coated glass coverslips for an additional week of culture in the same medium. The EBs were fixed with 4% PFA and were subjected to immunocytochemistry analyses with anti-α-fetoprotein, anti-ß-III-tubulin and anti-SMA antibodies. These experiments were performed under atmospheric oxygen conditions (20% O2, 5% CO2).
TaqMan hPSC Scorecard analysis [Thermo Fisher Scientific] was performed according to the manufacturer' s instruction. The data were analysed on hPSC Scorecard analysis software.

Flow cytometry analysis

For evaluating SSEA4 protein expressions, the hiPSCs were dissociated into single cells using TrypLE Select, pelleted and washed with PBS. The cells were then incubated with an antibody against SSEA4 [560796, BD Biosciences] on ice for 30 minutes. The cells that were positive for SSEA4 were isolated by fluorescence-activated cell sorting on a FACS Aria [Becton Dickinson] instrument. Unstained cells were used as a negative control. For evaluating TdTomato protein expressions, TdTomato fluorescence was directly analyzed by FACS Aria. P-hiPSCs were used as a negative control.

Neural differentiation assay

Transgene-independent cells were used for the neural differentiation assay of naïve hiPSCs by removing dox for at least five days before neural induction. The stromal cell-derived inducing activity (SDIA) methods were performed according to a modification of the method described in (Kawasaki et al., 2000). Briefly, the PA6 cells were maintained in α-MEM medium [Life Technologies] containing 10% FBS. PA6 cells plated on gelatin-coated plates were used as a feeder cell layer. To induce neural differentiation, primed and naïve hiPS cells that were pretreated with Y27632 were dissociated into single cells. The cells were grown at densities of 4×10^3–1.2×10^4

cells/well in 12-well plates in GMEM [Sigma] containing 10% KSR, 1 mM sodium pyruvate [Sigma], 1% NEAA and 0.1 mM 2-ME on a PA6 feeder layer. The medium was changed every two or three days. After 10 days, the cells were passaged using TrypLE Select or fixed with 4% PFA. The passaged cells were cultured under the same conditions. The fixed cells were subjected to immunocytochemistry analysis with an anti-MAP2 antibody. For the quantitative analysis, the numbers of colonies containing at least 10 MAP2-positive cells were counted, and the results are presented as the percentages of the total colonies. All (or up to 100) colonies were examined in each experiment. Moreover, the neurosphere-based neural differentiation assays were performed according to a modification of the method described in (Okada et al., 2008). Briefly, the primed and naïve hiPSC colonies were detached en bloc using the dissociation solution and by pipetting, respectively, (day 0) and cultured as suspensions in bacteriological dishes to form EBs. The EBs were cultured in basal medium for each hiPSC culture (hESC medium without FGF-2 for the primed iPSC colonies and N2B27 medium for the naïve iPSC colonies). One hundred nanomolar LDN193189 [StemRD] were added to enhance the neural induction (day 0-7). On day 7, the EBs were enzymatically dissociated into single cells using TrypLE Select, and the dissociated cells were cultured in suspension at a density of 2×10^5 cells/mL in media hormone mix (MHM) medium with 2% B27 supplement and 20 ng/ml FGF-2 to form the primary neurospheres. For the secondary neurospheres, the primary neurospheres were dissociated into single cells using TrypLE Select and cultured at 2×10^5 cells/mL in MHM with B27 supplement and FGF-2 (day 14). To assay the differentiation, the neurospheres were plated on poly-L-ornithine/fibronectin-coated cover glasses and allowed to differentiate in MHM medium containing 2% FBS and 20 ng/mL human LIF for 10 days (days 21-31). The differentiated neurospheres were fixed with 4% PFA and subjected to immunocytochemical analyses with anti-ß-III-tubulin and anti-GFAP antibodies. For the quantitative analysis, the numbers of β-III-tubulin-positive cells and GFAP-positive cells were counted at the peripheral parts of the attached neurospheres. Consequently, 473 cells from the 201B7-P-hiPSCs, 538 cells from the 201B7-N-hiPSCs, 1522 cells from the WD39-P-hiPSCs and 1080 cells from the WD39-N-hiPSCs were assessed.

Calcium imaging

Calcium imaging was performed in N-hiPSCs-derived astrocytes using a calcium indicator Fluo4 [Dojindo], according to the manufacturer' s instruction. Briefly, neural cells were differentiated from N-hiPSCs using the neurosphere-based method as

described above. The culture was trypsinized and replated onto laminin-coated 96well-plates. Subsequently, the cells were treated with Ara-C to ablate neurons. After stimulating the cells with 1mM ATP, time lapse imaging of the fluo-4 fluorescence was performed. Changes in the fluo-4 fluorescence intensity were quantified with the Metamorph software [Moleculardevices].

Supplemental References

Kanemura, Y., Mori, H., Kobayashi, S., Islam, O., Kodama, E., Yamamoto, A., Nakanishi, Y., Arita, N., Yamasaki, M., Okano, H., Hara, M., and Miyake, J. (2002). Evaluation of in vitro proliferative activity of human fetal neural stem/progenitor cells using indirect measurements of viable cells based on cellular metabolic activity. J. Neurosci. Res. 69, 869-879.

Nakamura, Y., Yamamoto, M., Oda, E., Yamamoto, A., Kanemura, Y., Hara, M., Suzuki, A., Yamasaki, M., and Okano, H. (2003). Expression of tubulin beta II in neural stem/progenitor cells and radial fibers during human fetal brain development. Lab. Invest. 83, 479-489.
