# Supplementary material for: Naive-like ESRRB+ iPSCs with the Capacity for Rapid Neural Differentiation
Source: Stem Cell Reports. 2017 Nov 9;9(6):1825–38. doi: 10.1016/j.stemcr.2017.10.008 (PMC5785673; doi:10.1016/j.stemcr.2017.10.008)
Supplement: Document S2. Article plus Supplemental Information [file mmc3.pdf]

Stem Cell Reports
Article

OPEN ACCESS

Naive-like ESRRB+ iPSCs with the Capacity for Rapid Neural Differentiation

Fumihiko Kisa,[1,2,5] Seiji Shiozawa,[1,5] Keisuke Oda,[1,2] Sho Yoshimatsu,[1] Mari Nakamura,[1,3] Ikuko Koya,[1] Kenji Kawai,[4] Sadafumi Suzuki,[1] and Hideyuki Okano[1,*]

[1]Department of Physiology, School of Medicine, Keio University, 35 Shinanomachi, Shinjuku-ku, Tokyo 160-8582, Japan
[2]Discovery Research Laboratories I, Minase Research Institute, Ono Pharmaceutical Co., Ltd., 3-1-1 Sakurai, Shimamoto, Mishima, Osaka 618-8585, Japan
[3]Department of Biomedical Chemistry, Graduate School of Medicine, The University of Tokyo, 7-3-1 Hongo, Bunkyo-ku, Tokyo 113-0033, Japan
[4]Pathological Analysis Center, Central Institute for Experimental Animals, 3-25-12 Tonomachi, Kawasaki, Kanagawa 210-0821, Japan
[5]Co-first author
*Correspondence: hidokano@a2.keio.jp
https://doi.org/10.1016/j.stemcr.2017.10.008

SUMMARY

Several groups have reported the existence of a form of pluripotency that resembles that of mouse embryonic stem cells (mESCs), i.e., a naive state, in human pluripotent stem cells; however, the characteristics vary between reports. The nuclear receptor ESRRB is expressed in mESCs and plays a significant role in their self-renewal, but its expression has not been observed in most naive-like human induced pluripotent stem cells (hiPSCs). In this study, we modified several methods for converting hiPSCs into a naive state through the transgenic expression of several reprogramming factors. The resulting cells express the components of the core transcriptional network of mESCs, including ESRRB, at high levels, which suggests the existence of naive-state hiPSCs that are similar to mESCs. We also demonstrate that these cells differentiate more readily into neural cells than do conventional hiPSCs. These features may be beneficial for their use in disease modeling and regenerative medicine.

INTRODUCTION

Human pluripotent stem cells (hPSCs), including embryonic stem cells (hESCs) and induced pluripotent stem cells (hiPSCs), exhibit characteristics that are distinct from those of mouse ESCs (mESCs), which are derived from the inner cell mass (ICM) of blastocyst-stage embryos (Thomson et al., 1998; Takahashi et al., 2007; Martin, 1981; Evans and Kaufman, 1981). These different characteristics may reflect differences in developmental stage because hESCs/hiPSCs share many characteristics with mouse epiblast stem cells (mEpiSCs), which are derived from post-implantation embryos (Brons et al., 2007; Tesar et al., 2007). The pluripotent state of mEpiSCs has been called the "primed" state to distinguish it from the "naive" pluripotency of mESCs. Conventional hPSCs have also been characterized as being in the primed state (Nichols and Smith, 2009). Based on the hypothesis that pluripotency state changes as development proceeds, it has been suggested that human cells may exhibit a naive form of pluripotency that corresponds to that of mESCs. Recently, several groups have reported the conversion of hPSCs from a primed to a naive state (Hanna et al., 2010; Gafni et al., 2013; Chan et al., 2013; Takashima et al., 2014; Theunissen et al., 2014; Valamehr et al., 2014; Wang et al., 2014; Ware et al., 2014; Chen et al., 2015; Duggal et al., 2015; Hayashi et al., 2015; Qin et al., 2016). However, due to differences in the methods used and the characteristics of the resulting naive-like cells, the existence of a bona fide naive pluripotent state of human iPSCs remains controversial. For example, human naive-like cells that are converted without the use of transgenes remain dependent on fibroblast growth factor (FGF) or transforming growth factor β (TGF-β) (Chan et al., 2013; Gafni et al., 2013; Valamehr et al., 2014; Ware et al., 2014; Duggal et al., 2015; Qin et al., 2016), which is a major property of the primed state (Vallier et al., 2005). Other groups have reported the conversion of cells from the primed to the naive state using transgenes (Hanna et al., 2010; Takashima et al., 2014; Qin et al., 2016), and the resulting cells appear to be more similar to naive mESCs. Takashima et al. reported the conversion of primed hESCs to a naive state via the transgenic expression of KLF2 and NANOG, both of which have been reported to efficiently reprogram mouse primed PSCs to the naive state (Hall et al., 2009; Silva et al., 2009). However, the resulting cells do not exhibit stable upregulation of ESRRB, which is a member of the core naive pluripotency transcription network in mESCs, in the absence of the continuous expression of the transgenes. Whether the difference between mouse and human ESRRB expression in "naive" pluripotent cells is attributable to interspecies differences or insufficient reprogramming remains controversial. Furthermore, the practical advantages of reprogrammed naive human pluripotent cells also remain unclear because the differentiation potential of human naive cells into specific lineages is not well known.

In the present study, we modified several conversion methods for the reprogramming of hiPSCs to a naive-like state using a combination of transcription factors and culture conditions. The reprogrammed cells exhibit elevated expression of the full set of naive pluripotency-related transcription factors that is known in mESCs, including *ESRRB*,

This is an open access article under the CC BY license (http://creativecommons.org/licenses/by/4.0/).

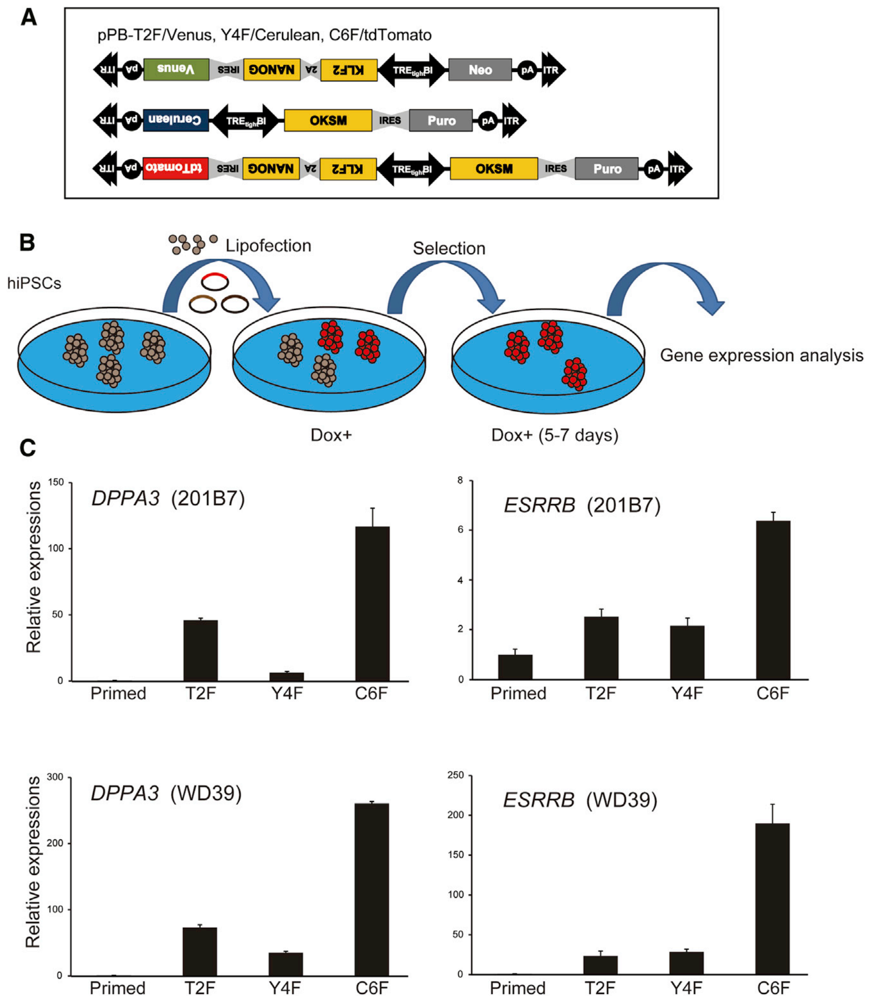

Figure 1. A Set of Reprogramming Factors for Robust Conversion to Naive State Pluripotency

(A) Constructs used to express the reprogramming factors. ITR, inverted terminal repeat; pA, polyadenylation signal; TRE, tetracycline response element; Neo, neomycin-resistance gene; Puro, puromycin-resistance gene; IRES, internal ribosome entry sites; OKSM, the set of *OCT3/4*, *KLF4*, *SOX2*, and *c-MYC*.

(B) Schematic presentation of the method used to evaluate the reprogramming factors. The red colonies represent the expression of Tomato, suggesting the exogenous expression of C6F.

(C) qPCR analyses of the expressions of the naive marker genes (*DPPA3* and *ESRRB*) in the reprogrammed 201B7 and WD39 cells suggesting a robust reprogramming with C6F compared with T2F and Y4F (n = 3, mean ± SEM; independent experiments). See also Figure S1.

which suggests the existence of a state of naive pluripotency in human cells that more closely resembles that of mESCs compared with the cells in previous reports. We also demonstrate that these naive-like hiPSCs exhibit enhanced abilities to differentiate into neural cells.

RESULTS

Robust Reprogramming of hiPSCs to a Naive-like State via the Expression of Six Transcription Factors

The co-expressions of *KLF2* and *KLF4*, *OCT3/4* and *KLF4*, and *KLF2* and *NANOG* have all been used to reprogram human pluripotent cells to a naive-like state (Hanna et al., 2010; Takashima et al., 2014). The evidence for conversion to a naive state via the combination of KLF2 and NANOG is particularly compelling (Hall et al., 2009; Silva et al., 2009; Theunissen et al., 2014; Takashima et al., 2014). In the present study, we assessed the efficiency of reprogramming methods involving three sets of transcription factors, i.e., the so-called Takashima two-factor (T2F: *KLF2* and *NANOG*), the Yamanaka four-factor (Y4F: *OCT3/4*, *KLF4*, *SOX2*, and *c-MYC*), and the combinatorial six-factor (C6F: *OCT3/4*, *KLF4*, *SOX2*, *c-MYC*, *KLF2*, and *NANOG*) methods, in hiPSCs (Figure 1A). We introduced a doxycycline (Dox)-inducible piggyBac transposon vector carrying T2F/Venus, Y4F/Cerulean, or C6F/TdTomato along with reverse tetracycline transactivator (rtTA) and piggyBac transposase expression vectors (Yusa et al., 2011) into two hiPSC lines, i.e., 201B7 and WD39 (Figure 1B). After adding Dox, we selected transgene-expressing cells grown in conventional hESC culture medium, and measured the expressions of *ESRRB* and *DPPA3*, which are highly expressed in naive mESCs but not in primed mEpiSCs, as naive marker genes. The expressions of *ESRRB* and *DPPA3* were most highly elevated in the C6F-expressing cells, although the expressions of these genes were also elevated relative to the primed-state counterparts via the ectopic expression of

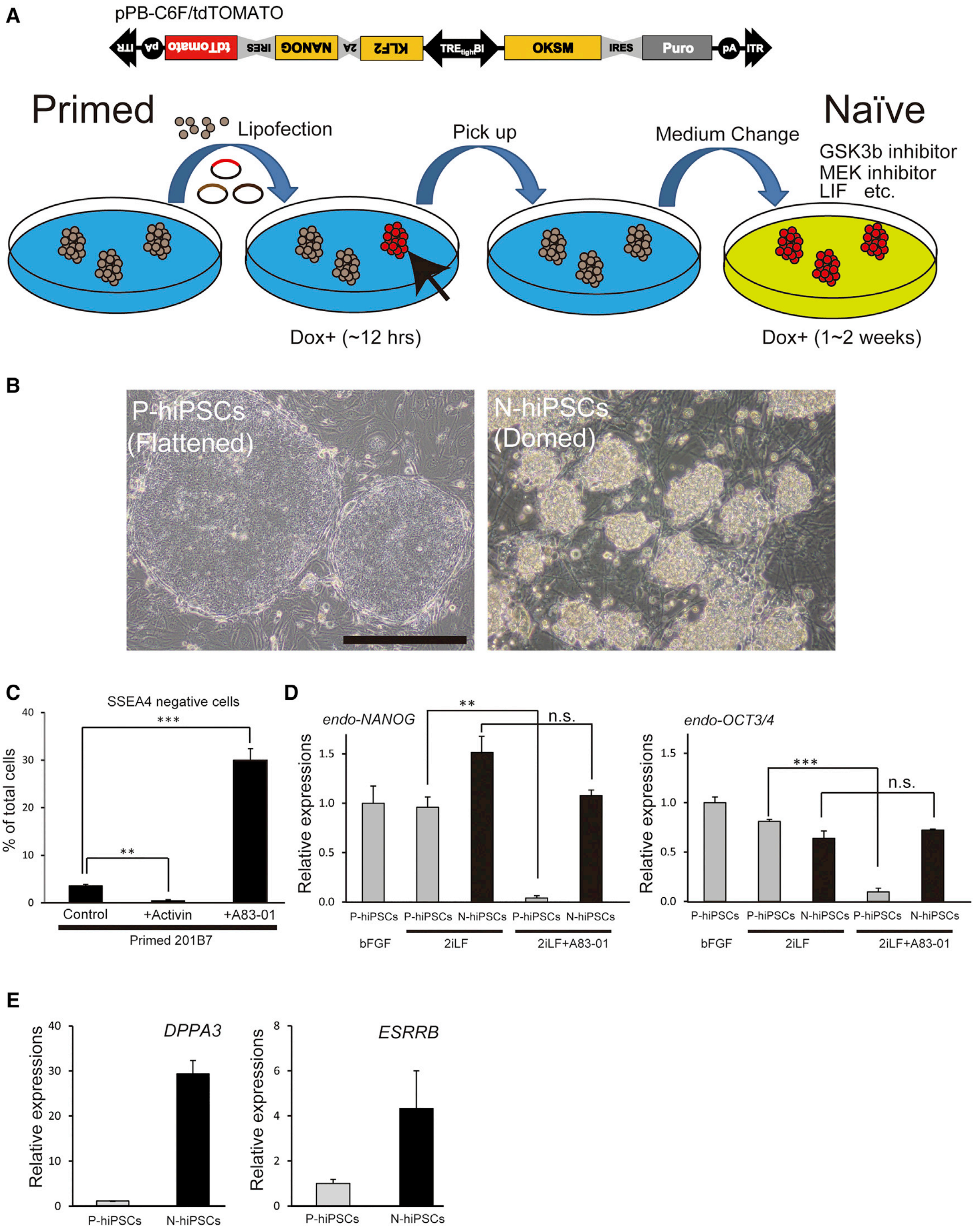

Figure 2. Generation of the Naive-like hiPSC Lines with the piggyBac Transposon

(A) Schematic presentation of the protocol for the generation of naive-like hiPSC lines via the piggyBac transposon. The black arrow indicates a transgene-expressing colony.

(B) Phase images of a clonal line of transgenic 201B7 hiPSCs cultured in a conventional primed condition without Dox (P-hiPSCs) and in 2iLF with Dox (N-hiPSCs). The N-hiPSCs formed dome-shaped colonies. Scale bar, 500 μm.

(C) TGF-β/Activin signal responsivity of the conventional hiPSCs. Control iPSC clones (201B7) cultured with activin or A83-01, which is a TGF-β/Activin signal inhibitor, were subjected to FACS analysis for the expression of SSEA-4, which is a marker of pluripotency (n = 3,

(legend continued on next page)

T2F alone (Figure 1C). These findings suggest a cooperative effect of T2F and Y4F on naive conversion. We also compared our C6F transgene method with the transgene-free culture methods described by Gafni et al. (2013) (NHSM) and Theunissen et al. (2014) (6i/L/A). Similar to the reports of these authors, the expression of *DPPA3* was elevated in the 6i/L/A condition but not in the NHSM condition, and the expression of *ESRRB* was not elevated in either condition (Figure S1A). These results suggest that reprogramming to a bona fide naive state requires transgene expression and that C6F is the most powerful set of transcription factors for this purpose, especially in terms of the upregulation of naive marker gene expression.

mESC-like Growth Properties of Naive-like hiPSCs

To further evaluate the characteristics of the naive-like hiPSCs that were converted by the C6F method, we established two transgenic lines harboring C6F/TdTomato and rtTA transgenes. In brief, hiPSC lines 201B7 and WD39 were transfected with a Dox-inducible piggyBac transposon vector carrying C6F/TdTomato and rtTA along with a transposase expression vector. These cells were selected with hygromycin under culture conditions conventionally used for primed pluripotent cells. After transient treatment with Dox, suitable colonies were selected based on Dox-inducible TdTomato expression and expanded by subculturing. The cells were maintained in the primed condition without Dox (P-hiPSCs).

For the conversion to a naive pluripotent state, the cells were cultured in a medium containing the cytokine leukemia inhibitory factor (LIF), Dox, and cocktail of small molecules (N-hiPSCs) (Figure 2A). Most of the naive-like human ESCs/iPSCs that have been reported to date have been maintained in medium containing 2iL, a combination of MEK (mitogen-activated protein kinase kinase) and GSK3 (glycogen synthase kinase 3) inhibitors with LIF (Ying et al., 2008; Marks et al., 2012). Initially, we also regarded 2iL as essential to the maintenance of naive-like pluripotent cells and thus added it to the KSR (KnockOut serum replacement)-based medium. We also added forskolin to the 2iL (2iLF) based on previously reported methods (Hanna et al., 2010; Duggal et al., 2015; Qin et al., 2016). The N-hiPSCs cultured in 2iLF exhibited a tightly packed domed morphology (Figure 2B). Additionally, similar to mESCs, the N-hiPSCs could be passaged as single cells using trypsin/EDTA without the addition of a ROCK inhibitor (Figure S2) (Watanabe et al., 2007).

TGF-β/Activin Signal Independence of C6F-Expressing hiPSCs

The promotion of self-renewal by TGF-β/Activin signaling has been regarded as a characteristic of primed pluripotent stem cells. To explore the differences in the growth factor requirements of P-hiPSCs and N-hiPSCs, we applied activin or A83-01, which is a pharmacological inhibitor of TGF-β/Activin signals, to P-hiPSCs for 5 days and analyzed the expression of SSEA4, which is a cell-surface marker of pluripotent stem cells, via flow cytometry. As expected, the number of SSEA4-positive cells increased following the addition of activin and decreased following treatment with an activin inhibitor (Figure 2C), which indicates that the P-hiPSCs exhibited characteristics consistent with primed-state pluripotency. Next, we examined the responsivity of N-hiPSCs to TGF-β/Activin signaling. We cultured N-hiPSCs in 2iLF in the presence of A83-01 (2iLFA) for 5 days and performed qPCR analysis. The expressions of endogenous pluripotency markers were sustained at considerable levels in the N-hiPSCs with 2iLFA, although the levels in the P-hiPSCs were dramatically decreased (Figure 2D). Additionally, the N-hiPSCs cultured in 2iLFA maintained high expression levels of the naive marker genes *ESRRB* and *DPPA3* (Figures 2E and S3A–S3C). These data indicate that N-hiPSCs but not P-hiPSCs can be maintained without TGF-β/Activin signaling.

Three-Germ-Layer Differentiation Potential of Naive-like hiPSCs

To confirm the differentiation potential of the N-hiPSCs, we performed embryoid body formation assays and observed that both the P-hiPSCs and N-hiPSCs were able to differentiate into all three germ layers *in vitro* (Figure S4A). Furthermore, the differences of the differentiation potentials between P-hiPSCs and N-hiPSCs were evaluated with the TaqMan hPSC Scorecard assay (Tsankov et al., 2015). Results from the assay suggested that N-hiPSCs had a higher three-germ-layer differentiation potential (Figures S4B–S4D).

mean ± SEM; independent experiments; $^{**}p < 0.01$; $^{***}p < 0.001$; t test). Activin supports the pluripotency of primed cells, whereas A83-01 deteriorates it.

(D) TGF-β/Activin signal independency of the reprogrammed cells. P-hiPSCs (without Dox) and N-hiPSCs (with Dox) were cultured in 2iLF, which is a medium that contains LIF, PD0325901, CHIR99021, and forskolin, with or without A83-01. The endogenous expressions of the pluripotency marker genes (*OCT3/4* and *NANOG*) were analyzed by qPCR (n = 3, mean ± SEM; independent experiments; $^{**}p < 0.\ 01$; $^{***}p < 0.001$; n.s., not significant; t test).

(E) qPCR analyses of naive marker gene (*DPPA3* and *ESRRB*) expressions in the 201B7 P-hiPSCs and N-hiPSCs (n = 3, mean ± SEM; independent experiments).

These results indicate that N-hiPSCs exhibit the capacity for differentiation with high levels of naive marker gene expression.

Optimization of the Culture Conditions

Whereas naive pluripotency was stably maintained in the N-hiPSCs in the presence of Dox, it became unstable after withdrawal of Dox (Figure 3A). Hence, we tested to identify the optimal medium conditions that were able to stably maintain the pluripotency of the N-hiPSCs after the removal of Dox (Figure 3B). First, we used an N2B27-based medium supplemented with 2iL and a protein kinase C (PKC) inhibitor (Gö6983) as previously reported (Dutta et al., 2011; Takashima et al., 2014). However, the N-hiPSC colonies reverted to a flattened morphology upon the withdrawal of Dox. Next, we examined the combination of our previous medium conditions and Gö6983. Although domed-colony morphologies could be maintained in the absence of Dox in these conditions, the cells could not be repeatedly propagated due to low viability. Therefore, we further tested a number of modifications of our culture with the aim of improving viability. Alteration of the basal medium to an N2B27-based medium from a KSR-based medium dramatically improved cell viability. The N-hiPSCs could be passaged while maintaining the domed-colony morphology after the withdrawal of Dox (Figure 3C). Furthermore, qPCR analysis revealed that the expressions of pluripotency markers and naive markers were also maintained after the withdrawal of Dox (Figure 3D). The precise control of transgene expression by Dox was confirmed by qPCR (Figure S1D), as well as fluorescence-activated cell sorting (FACS) analysis of TdTomato expression (Figures S1B and S1C). Moreover, we confirmed that the N-hiPSCs that were cultured in N2B27-based 2iLFA medium supplemented with Gö6983 were also capable of differentiating into all three germ layers (Figure S4A).

LIF/Stat3 Signal Dependence of N-hiPSCs

It is well known that naive mESCs require LIF/JAK/STAT signaling for sustained self-renewal (Smith et al., 1988; Williams et al., 1988; Niwa et al., 2009). To identify the differences in the role of the LIF signal in human naive and primed states, we treated N-hiPSCs and P-hiPSCs with a JAK inhibitor (JAK Inhibitor I) and evaluated the effects via qPCR analysis. In the N-hiPSCs, the expression levels of pluripotency markers were significantly decreased upon inhibition of JAK1 (Figure 4A), whereas these levels were not altered in the P-hiPSCs (Figure 4B). Moreover, the expressions of naive marker genes (*DPPA3*) and a downstream target gene of the LIF signal (*SOCS3* and *KLF4*) were also downregulated by JAK1 inhibition in the N-hiPSCs, whereas the expression of *TFCP2L1* was unaffected. As previously reported in mESCs, CHIR99021 treatment may have compensated for the TFCP2L1 expression (Ye et al., 2013; Martello et al., 2013).

These results suggested the existence of an mESC-like JAK/STAT signal circuit in the N-hiPSCs. Taken together with the TGF-β/Activin signal independence, we concluded that the N-hiPSCs exhibited growth factor requirements that were highly similar to those of mESCs.

Alteration of TFE3 Subcellular Localization

TFE3 is distributed in both the nucleus and cytoplasm in naive mESCs, while nuclear TFE3 translocates into the cytoplasm at the onset of mESC differentiation (Betschinger et al., 2013). Similar to mouse cells, TFE3 has been demonstrated to be enriched in the nucleus when human primed cells are converted to the naive state (Gafni et al., 2013; Takashima et al., 2014). We confirmed that our N-hiPSCs also exhibited a nuclear localization of TFE3 that contrasted with the cytoplasmic localization observed in the P-hiPSCs according to the immunocytochemical analysis (Figure S3D).

Gene Expression

We performed RNA sequencing (RNA-seq) analysis to characterize the global gene expression profile of the N-hiPSCs under the optimized culture conditions. For comparison, three independent cultures of N-hiPSCs in the absence of Dox and P-hiPSCs were analyzed, and significant differences were observed between these cells. Most of the core transcription factors in the ground-state self-renewal, including *KLF2*, *KLF4*, *TFCP2L1*, and *TBX3*, were upregulated in the N-hiPSCs (Figure 5A). In contrast, *SOX2* was slightly downregulated in the N-hiPSCs, although the expression levels of all of these genes were relatively high compared with those of other genes. The expression of *ESRRB*, which is an important factor in ground-state self-renewal, was upregulated in the N-hiPSCs that were generated using the method reported here, which contrasts with most previous reports of naive-like human pluripotent cells (Figures S3A–S3C). Moreover, *KLF17*, which was recently reported to be a reliable naive marker (Boroviak et al., 2015; Blakeley et al., 2015; Guo et al., 2016), was also upregulated in our N-hiPSCs. To verify our RNA-seq data, we performed qPCR analysis (Figure 5C) and found that the expressions of naive marker genes, including *ESRRB*, *DPPA3*, *KLF2*, *KLF4*, *KLF5*, *TFCP2L1*, and *TBX3*, were increased in the N-hiPSCs even after the withdrawal of Dox. Additionally we confirmed that the expressions of pluripotency markers were maintained at high levels and that the expression of a primed marker gene (*LEFTY*) was downregulated in the N-hiPSCs. Similar results were obtained from another hiPSC line, i.e., WD39-derived N-hiPSCs (Figure 5C).

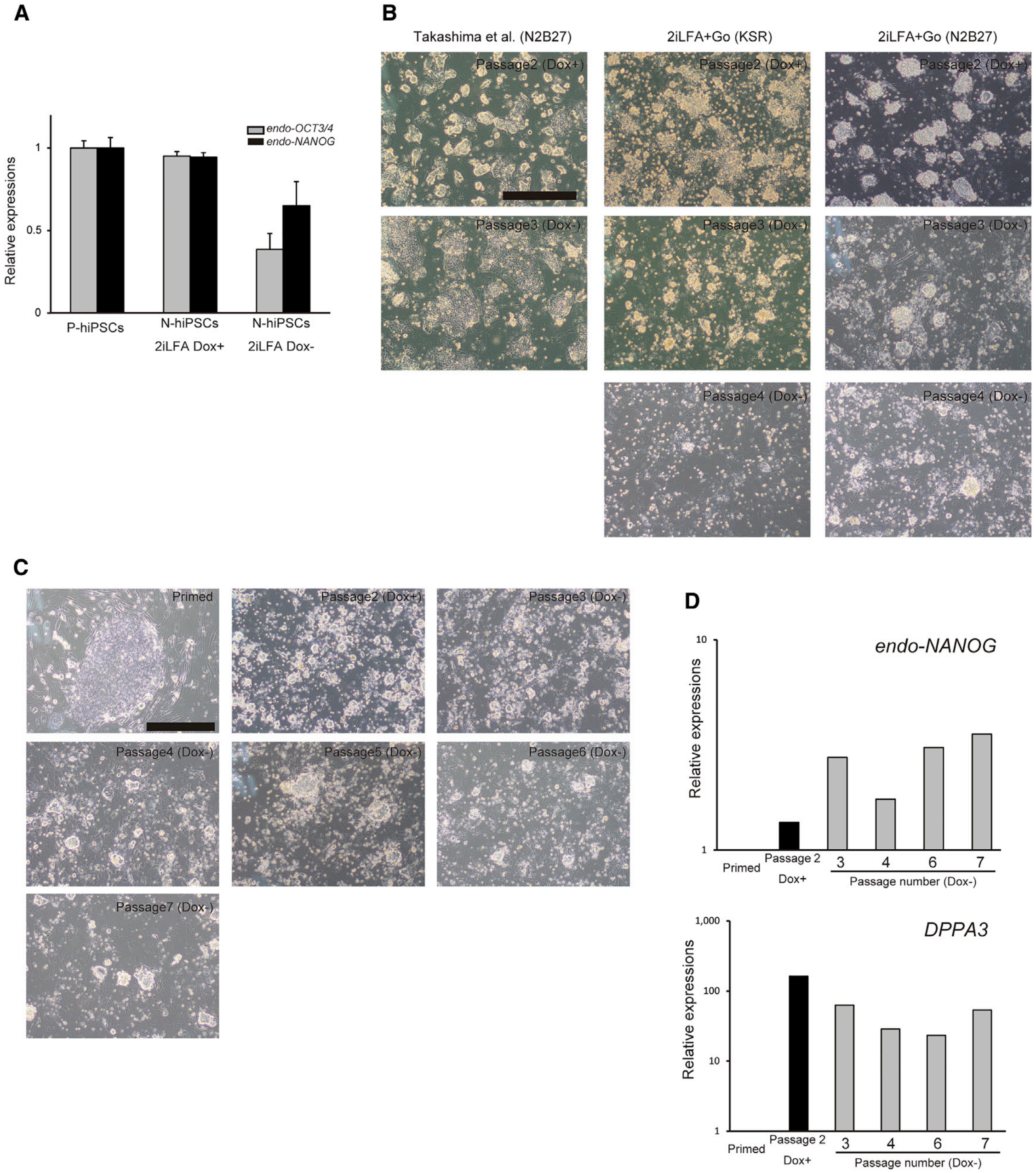

Figure 3. Culture Conditions for the Naive-like hiPSCs

(A) qPCR analyses of pluripotency marker gene (*OCT3/4* and *NANOG*) expressions in naive-like cells (201B7 N-hiPSCs) cultured in 2iLFA before and after the withdrawal of Dox, and in primed cells (201B7 P-hiPSCs) (n = 3, mean ± SEM; independent experiments).

(B) Phase images of naive-like cells (201B7 N-hiPSCs) cultured in three different media before and after the withdrawal of Dox. 2iLFA + Go (KSR), KSR-based medium supplemented with 2iL, forskolin, TGF-β inhibitor, and PKC inhibitor; 2iLFA + Go (N2B27), N2B27-based medium supplemented with 2iL, forskolin, TGF-β inhibitor, and PKC inhibitor; Takashima et al. (N2B27), N2B27-based medium supplemented with 2iL and PKC inhibitor. Scale bar, 500 μm.

(C) Phase images of 201B7 N-hiPSCs maintained in 2iLFA + Go (N2B27) through passages. Dox was withdrawn at passage 3. Scale bar, 500 μm.

(D) qPCR analysis of *DPPA3* and *NANOG* expressions in the 201B7 N-hiPSCs through the passages.

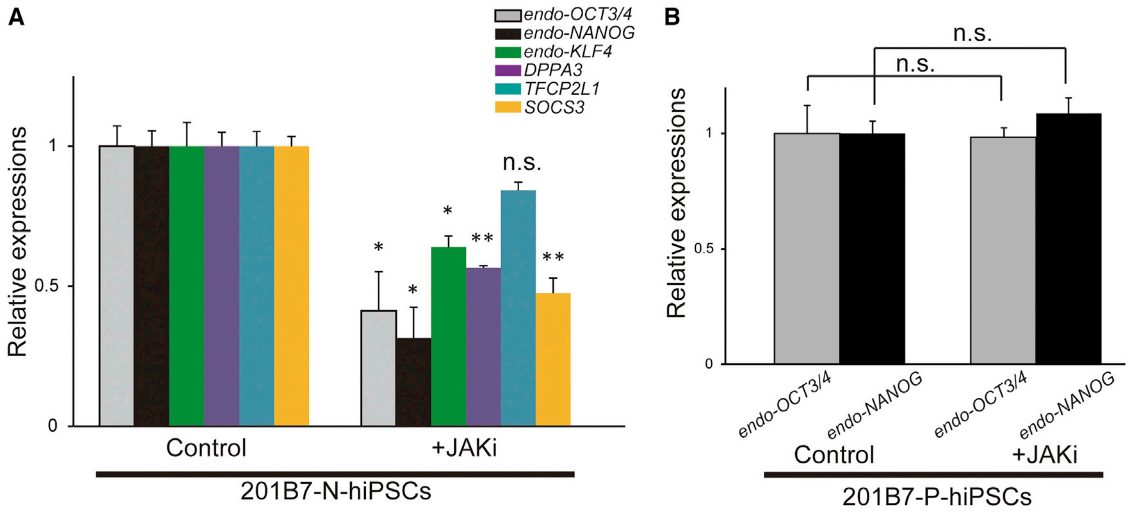

Figure 4. Jak/Stat3 Signal Responsivity

qPCR analysis of the 201B7 N-hiPSCs (A) and P-hiPSCs (B) with and without a Jak inhibitor (Jaki) for the endogenous expressions of Jak/Stat3 signaling-related genes and pluripotency-related genes (n = 3, mean ± SEM; independent experiments; $^{*}p < 0.05$; $^{**}p < 0.01$; n.s., not significant; t test).

Finally, the whole-transcriptome profiles of these cells were compared with those of human blastocyst ICMs, reset cells, and human naive ESCs derived directly from ICMs (HNES) using published RNA-seq datasets (Yan et al., 2013; Takashima et al., 2014; Blakeley et al., 2015; Guo et al., 2016; Petropoulos et al., 2016). Our N-hiPSCs exhibited a transcriptome that was similar to those of the human blastocyst ICMs, reset cells, and HNES (Figures 5D and 5E). The expression pattern of the N-hiPSCs was quite similar to that of the human blastocyst ICMs with respect to the known core transcription factors in ground-state self-renewal (Figures 5A and 5B). Clustering by principal component analysis using the differently expressed genes with higher-fold changes (a $\log_2$ fold change of FPKM [fragments per kilobase of transcript per million mapped reads] > 3) revealed that the principal component 1 clearly discriminated the primed cells and naive cells (Figure 5F). Interestingly, focusing on each HNES sample separately revealed that some of the HNES exhibited moderate expression of *ESRRB* (Figure 5E), which supports the existence of a mESC-like naive pluripotent state in human in terms of *ESRRB* expression.

Rapid and Efficient Neural Differentiation of Naive-like hiPSCs

To investigate the potential practical advantages of the naive-like hiPSCs in terms of differentiation, we compared the capacities for neural differentiation of the naive and primed hiPSCs using two differentiation methods. First, we applied the stromal cell-derived inducing activity (SDIA) method (Kawasaki et al., 2000) (Figure 6A). Because the SDIA method is a simple protocol whereby PSCs are simply co-cultured with PA6 stromal cells, it is well suited for assessing differentiation potential. We co-cultured P-hiPSCs and N-hiPSCs with PA6 cells, subjected the cells to immunocytochemical analysis for the neuronal marker MAP2 (Figure 6B) after 10 days in culture, and quantified the percentage of colonies that contained differentiated neurons. Surprisingly, the N-hiPSCs efficiently differentiated into neurons, whereas the P-hiPSCs rarely differentiated into neurons at this time point (Figure 6C). The P-hiPSCs required further cultivation for 10 days to give rise to neurons under the same conditions (Figure S5).

Next, we used a neurosphere-based method (Okada et al., 2008; Imaizumi et al., 2012) (Figure 6D) whereby primed and naive hiPSCs were differentiated into neural stem/progenitor cells as neurospheres through embryoid body formation. Secondary neurospheres were plated and allowed to differentiate for 10 days. The differentiated neurospheres were subjected to immunocytochemical analysis for βIII-tubulin (neurons) and glial fibrillary acidic protein (GFAP) (astrocytes) (Figure 6E). Unexpectedly, while the P-hiPSCs differentiated into βIII-tubulin-positive neurons, the N-hiPSCs predominantly differentiated into GFAP-positive astrocytes. These GFAP-positive astrocytes express other astrocyte markers (Figures S6A and S6B), and their activity was confirmed by calcium imaging (Figure S6C and Movie S1). As illustrated in Figure 6F, the percentages of GFAP-positive astrocytes were 18.5% and 20.5% in the cultures from the 201B7 N-hiPSCs and the WD39 N-hiPSCs, respectively, whereas no GFAP-positive

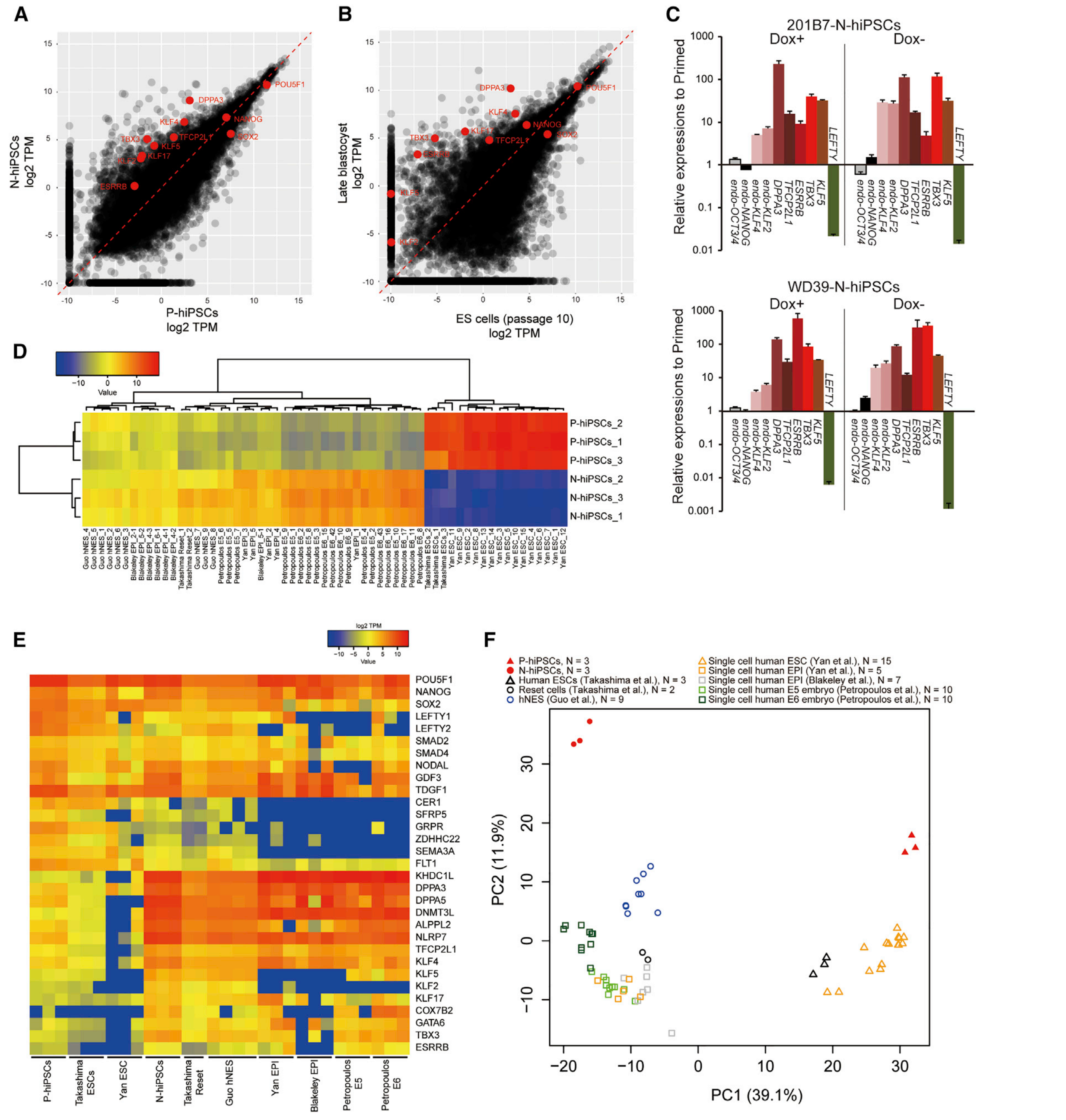

Figure 5. Gene Expression Analysis

(A) Scatterplot of the P-hiPSCs versus the N-hiPSCs for the TPM values of each gene from RNA-seq data from this study.

(B) Scatterplot of the hESCs versus the late blastocysts for the TPM values of each gene from the single-cell RNA-seq data from the study of Yan et al. (2013).

(C) qPCR analyses of the gene expressions in the N-hiPSCs cultured in 2iLFA + Go (N2B27) before and after the withdrawal of Dox and in the primed counterparts. The reddish bars represent naive marker genes and the green bar represents a primed marker (n = 3; mean ± SEM; independent experiments).

(D) Correlation matrix of the TPM values from the RNA-seq data from this study and the studies of Yan et al. (2013), Takashima et al. (2014), Blakeley et al. (2015), Petropoulos et al. (2016), and Guo et al. (2016).

(E) Heatmap of the TPM values for selected genes from the RNA-seq data from this study with each dataset described above.

(F) Principal component (PC) analysis of RNA-seq data from this study with each dataset described above.

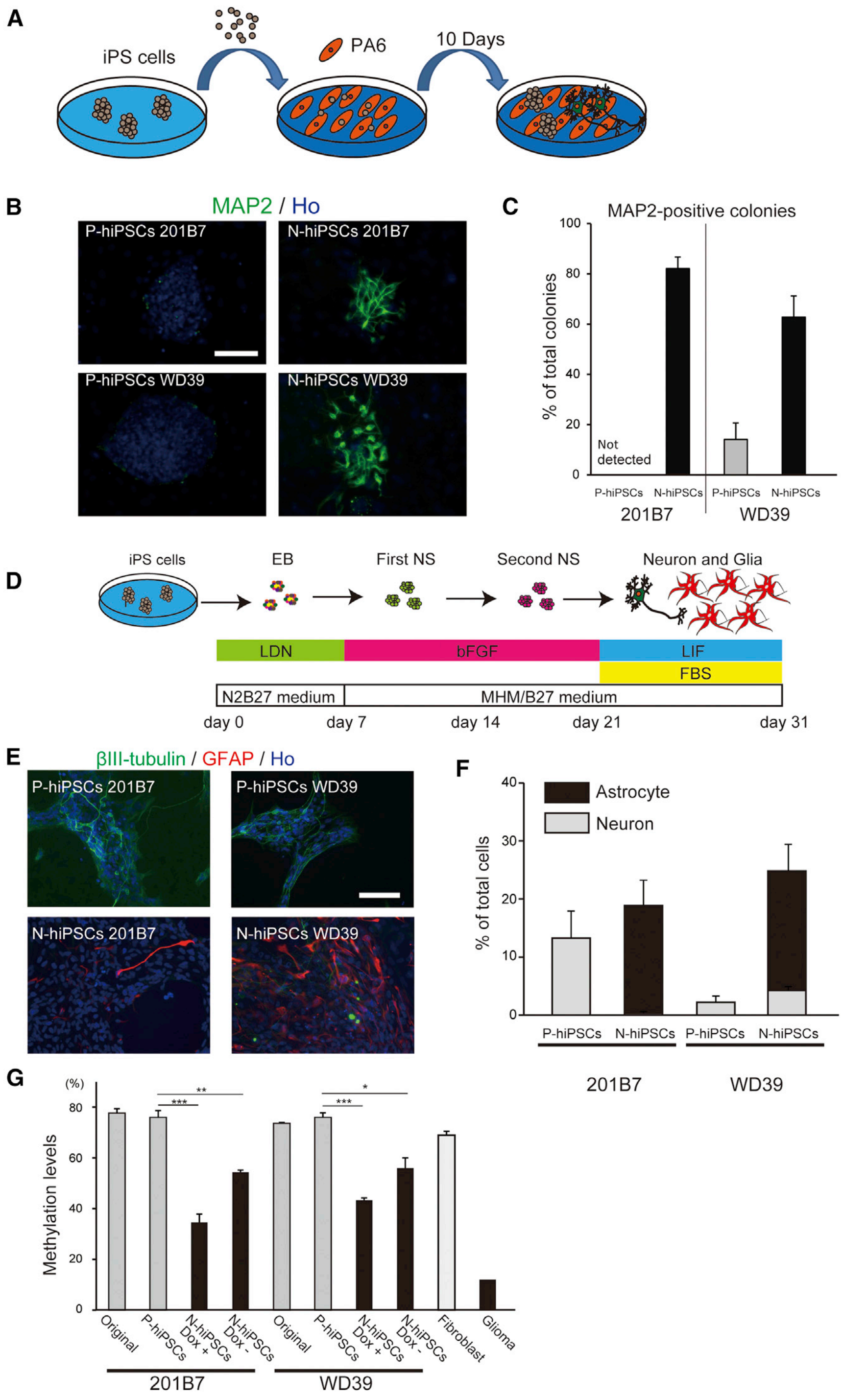

Figure 6. Neural Differentiation Capacities

(A) Schematic presentation of the SDIA method.

(B) Representative images of the immunocytochemistries of the differentiated iPSC colonies (neurons: MAP2). Scale bar, 100 μm.

(C) Quantitative analysis of the percentages of colonies containing neurons (n = 3–5; mean ± SEM; independent experiments).

(D) Schematic presentation of the protocols for the differentiation of astrocytes from hiPSCs.

(E) Representative images of the immunocytochemistries of two groups of neural lineage cells (neurons: β-III tubulin; astrocytes: GFAP). Scale bar, 50 μm.

(F) Quantitative analysis of the percentages of neurons and astrocytes in the differentiated neurospheres from the P-hiPSCs and N-hiPSCs (n = 3; mean ± SEM; independent experiments).

(G) Frequency of STAT3 binding site methylation in the GFAP promoter as analyzed by bisulfite pyrosequencing (n = 3; mean ± SEM; independent experiments; *$p < 0.05$; **$p < 0.01$; ***$p < 0.001$; t test).

See also Figures S5 and S6.

astrocytes were differentiated from their primed counterparts at this stage (Figure S6A).

To explore the reason for this greater gliogenic competency of the N-hiPSCs, we checked the methylation status of the CpG dinucleotide at the STAT3 binding site in the GFAP promoter because the epigenetic gene regulation of GFAP at this site is important in the switch from neurogenesis to gliogenesis (Takizawa et al., 2001; Fan et al., 2005; Andoh-Noda et al., 2015). Both pyrosequencing and bisulfite sequencing analysis revealed a state of lower methylation in the N-hiPSCs than in the P-hiPSCs (Figures 6G and S6D).

In conclusion, the naive-like hiPSCs exhibit high neural differentiation potential compared with the conventional primed hiPSCs.

DISCUSSION

The existence of bona fide human naive pluripotent stem cells remains controversial, although various methods have been reported for maintaining hPSCs in states similar to those of mESCs (Hanna et al., 2010; Gafni et al., 2013; Chan et al., 2013; Takashima et al., 2014; Theunissen et al., 2014; Valamehr et al., 2014; Wang et al., 2014; Ware et al., 2014; Chen et al., 2015; Duggal et al., 2015; Hayashi et al., 2015; Qin et al., 2016). Naive iPSCs generated directly from somatic cells have also been reported (Yang et al., 2016).

The results of the present study indicate that the ectopic expression of a set of six transcription factors (i.e., OCT3/4, SOX2, KLF4, C-MYC, KLF2, and NANOG) strongly reprograms hiPSCs to a naive-like state that more resembles mESCs than naive-like cells that are converted using only KLF2 and NANOG. This finding indicates that the four Yamanaka factors synergistically act with KLF2 and NANOG to enable conversion from a primed to a naive state.

The reprogrammed cells exhibited characteristics that were highly similar to those of mESCs with respect to morphology, growth factor requirements, and global gene expression. In contrast to most previous reports, our naive-like hiPSCs highly expressed all of the known components of the core transcription factor network of naive mESCs, including *ESRRB*, and differentiated into both neurons and astrocytes more efficiently than their primed counterparts.

Previously reported naive-state hPSCs can be propagated in an LIF-dependent state in a manner akin to that of mESCs using a cocktail of small molecules (Gafni et al., 2013; Chan et al., 2013; Theunissen et al., 2014; Valamehr et al., 2014; Ware et al., 2014; Duggal et al., 2015). However, similar to mEpiSCs and conventional hiPSCs/hESCs, these cells still depend on TGF-β, FGF-2, or both. These findings are suggestive of insufficient reprogramming, resulting in converted cells that are in an intermediate state between naive and primed. In contrast, hPSCs that are reprogrammed with C6F can be maintained without TGF-β or FGF-2. Our results are in agreement with the report by Takashima's group in which the short-term expression of NANOG and KLF2 transgenes (T2F) was used to reprogram human cells to be independent of TGF-β and FGF-2 (Takashima et al., 2014). These results led us to conjecture that the expression of an appropriate set of transcription factors would be able to more robustly convert human cells to a naive state than a change in medium conditions. Meanwhile, when applying this method in the field of regenerative medicine, the genomic integration of the transgenes will be problematic. Developing a method that is integration-free or not having to rely on transgenes will be essential in the future.

Culture conditions can also be important in the conversion and maintenance of altered pluripotent states. In this study, we used 2iL medium plus a PKC inhibitor, forskolin, and a TGF-β inhibitor for conversion and maintenance because some reports have suggested that a PKC inhibitor and forskolin facilitate conversion from the primed to the naive state (Hanna et al., 2010; Dutta et al., 2011; Takashima et al., 2014; Duggal et al., 2015; Qin et al., 2016). Additionally, TGF-β inhibitors may facilitate the selection of naive-like cells because primed cells cannot be propagated in medium containing a TGF-β inhibitor. Whether the TGF-β/Activin signaling is necessary for naive hPSCs is still a matter of discussion. In experiments using human blastocysts, inhibiting the TGF-β/Activin signal has led to contradictory results. In one report, the signal inhibition promoted epiblast formation (Van der Jeught et al., 2014) while in another, epiblast formation was inhibited (Blakeley et al., 2015). Furthermore, in experiments using nonhuman primate embryos, epiblast formation was neither promoted nor inhibited when TGF-β/Activin signaling was inhibited (Boroviak et al., 2015). The present results from our experiments suggest that the signal inhibition is not essential once the cells are converted into a naive-like state.

The transcription factor circuitries that govern pluripotency differ between naive and primed cells. For example, *Esrrb*, *Nanog*, *Klf2*, *Klf4*, and *Tfcp2l1* are highly expressed and play important roles in mESCs, whereas these genes are expressed at low levels in mEpiSCs and hPSCs. Although some of these genes can be elevated in human cells by naive-like conversion as described in previous reports, in most cases the expression of *ESRRB* is unaffected (Hanna et al., 2010; Gafni et al., 2013; Chan et al., 2013; Theunissen et al., 2014; Valamehr et al., 2014; Ware et al., 2014). In contrast, the naive-like hiPSCs in the present study maintained ESRRB expression at high levels even after transgene expression was suppressed. However, it remains controversial whether ESRRB is highly expressed in the bona fide human naive pluripotent cells. Blakeley et al. (2015) recently reported that some naive markers upregulated in rodents, including *KLF2* and *ESRRB*, are rarely expressed in the human epiblast. However, in our transcriptome analysis including datasets from previous reports, the epiblast datasets from Petropoulos et al. (2016) showed *ESRRB* expression. Since the blastocyst development is a dynamic process, differences in the blastocyst stage used in the analysis may have caused this discrepancy. On the other hand there is no *KLF2* expression in

any epiblast dataset, whereas its expression is observed in naive-type hPSCs expanded *in vitro*, including reset cells and NHES. Our results suggest that *ESRRB* and *KLF2* expression may not be critical for epiblast development but may play a role in the self-renewal of PSCs *in vitro*. Indeed, the *KLF2* knockout mouse survived until embryonic day 11.5–13.5 without any early developmental abnormality (Wani et al., 1998). Meanwhile, the *ESRRB* knockout mouse could not survive after embryonic day 10.5, but its death was caused by abnormalities in the placenta, which is not derived from the epiblast (Luo et al., 1997). Yet in *in vitro* culture, both genes play an important role for the self-renewal of naive-state mESCs (Festuccia et al., 2012; Martello et al., 2012; Yeo et al., 2014; Qiu et al., 2015). Analysis of our naive-like cells suggests that these genes may have some specific function in the *in vitro* expansion of human naive-state PSCs as well. Further studies are needed to explore the role of these genes in the self-renewal of naive human PSCs.

Little information is available regarding the differentiation potential of human naive cells. We obtained results suggesting that N-hiPSCs have a higher differentiation potential into the three germ layers through embryoid body formation. Furthermore, we performed neural differentiation assays and found that human naive-like cells differentiate more rapidly into neurons and astrocytes than do their primed counterparts. These results support the perspective that naive conversion is conducive to neural differentiation (Honda et al., 2013).

In addition, the human naive-like cells were also able to give rise rapidly to astrocytes. The length of developmental period of the animal species, from which the PSCs were derived, may be one factor that can affect the differentiation speed of the PSCs. This could possibly be the reason why mPSCs can differentiate into glial cells faster than human PSCs. Our data suggest that the starting pluripotent state can also affect the speed of glial differentiation *in vitro*. Although the detailed mechanism of the rapid glial differentiation of naive-like hiPSCs remains unclear, we found that the GFAP promoter region, i.e., the important locus for glial differentiation, is hypomethylated in naive-like hiPSCs compared with their primed counterparts.

Some reports have demonstrated that human naive cells exhibit genome-wide DNA hypomethylation (Gafni et al., 2013; Takashima et al., 2014), which is a feature of mESCs in 2iL medium and early embryonic epiblasts (Leitch et al., 2013; Habibi et al., 2013; Ficz et al., 2013; Guo et al., 2014). This hypomethylated genomic state may cause an increase in the sensitivity to differentiation signals. Additionally, a hypomethylation status may be beneficial to the maturation of hiPSC-derived differentiated cells because the maturation capacity of hiPSC-derived hematopoietic precursors has been reported to be related to the amount and pattern of DNA methylation acquired during reprogramming in hiPSCs (Nishizawa et al., 2016). Further investigations are needed to clarify the relations of methylation status and differentiation propensities.

Generally, primed pluripotency has been described as a priming phase for differentiation into various cell lineages. Notably, the propensity for differentiation among clones is diverse (Osafune et al., 2008; Liang and Zhang, 2013). In contrast, because the naive pluripotent state has no biased differentiation propensity (Nichols and Smith, 2009), naive-like hiPSCs are thought to be more responsive to extrinsic differentiation cues. This may be one factor underlying the rapid neural differentiation exhibited by naive-like hiPSCs. Findings from studies of mouse primed cells support this possibility because different mEpiSC lines exhibit distinct gene expression patterns, and some mEpiSC lines exhibit resistance to differentiation into neural lineages (Bernemann et al., 2011). Consistent with these findings, Song et al. (2016) report that, even in the cell line, mEpiSCs exhibit heterogeneity and biased differentiation potential. Moreover, Jang et al. (2014) reported that the global gene expression pattern of brain-derived NSCs is more similar to that of mESC-derived NSCs than that of mEpiSC-derived NSCs. Naive-like hiPSCs may thus be advantageous for maximizing the quality of hiPSC-derived differentiated cells.

In conclusion, this study provides a modified method for reprogramming human primed cells to a naive state in which all known naive pluripotency-related transcription factors, including *ESRRB*, are expressed. These naive-like hiPSCs exhibit efficient neural differentiation and rapid glial differentiation compared with conventional primed hiPSCs. These features of naive-like hiPSCs may facilitate disease modeling and regenerative medicine research using hPSCs because some types of cells that are difficult and/or time-consuming to obtain from conventional hiPSCs can be easily differentiated from naive-like hiPSCs.

EXPERIMENTAL PROCEDURES

See also Supplemental Experimental Procedures.

Cell Culture

The hiPSC lines 201B7 and WD39 (Imaizumi et al., 2012) were used in this study. For the conventional primed condition culture, the hiPSCs were grown on mitomycin-C-treated SNL murine fibroblast feeder cells or irradiated mouse embryonic fibroblasts in 0.1% gelatin-coated tissue culture dishes. The hiPSCs were maintained in standard hESC medium (DMEM/F12 [Wako] containing 20% KSR [Life Technologies], 2 mM L-glutamine [Nacalai Tesque], 1% non-essential amino acids [Sigma], 0.1 mM 2-mercaptoethanol [Sigma], and 4 ng/mL FGF-2 [PeproTech]) at 37°C in atmospheric

oxygen (20% O_2, 3% CO_2). The hiPSCs were subcultured with a dissociation solution (0.25% trypsin, 100 μg/mL collagenase IV [Invitrogen], 1 mM $CaCl_2$, and 20% KSR) every 5–7 days.

SUPPLEMENTAL INFORMATION

Supplemental Information includes Supplemental Experimental Procedures, six figures, one table, and one movie and can be found with this article online at https://doi.org/10.1016/j.stemcr.2017.10.008.

AUTHOR CONTRIBUTIONS

F.K., S. Shiozawa, and H.O. conceived and designed the experiments. F.K., K.O., S.Y., I.K., K.K., and S. Suzuki performed the experiments and analyzed data. F.K., S. Shiozawa, K.O., M.N., and H.O. wrote and edited the manuscript. All authors read and approved the final manuscript.

ACKNOWLEDGMENTS

We thank Drs. T. Sanosaka, D. Sipp, F. Renault-Mihara, and S. Morimoto for helpful discussions and technical assistance and all members of the H.O. laboratory for generous support. We also thank Prof. Shinya Yamanaka (Kyoto University) for the human iPSC (201B7). The piggyBac transposase expression vector was kindly provided by Dr. Kosuke Yusa (Wellcome Trust Sanger Institute). Portions of this study were the result of the "Construction of System for Spread of Primate Model Animals," which was performed under the Strategic Research Program for Brain Sciences of the MEXT and the AMED (to H.O and S.S.), and Scientific Research in Innovative Areas, which is the MEXT Grant-in-Aid Project FY2014-2018: "Brain Protein Aging and Dementia Control" (to H.O.). H.O. is a compensated scientific consultant of San Bio and K-Pharma. F.K. and K.O. are paid by the Ono Pharmaceutical.

Received: January 18, 2017
Revised: October 9, 2017
Accepted: October 10, 2017
Published: November 9, 2017

REFERENCES

Andoh-Noda, T., Akamatsu, W., Miyake, K., Matsumoto, T., Yamaguchi, R., Sanosaka, T., Okada, Y., Kobayashi, T., Ohyama, M., Nakashima, K., et al. (2015). Differentiation of multipotent neural stem cells derived from Rett syndrome patients is biased toward the astrocytic lineage. Mol. Brain *8*, 31.

Bernemann, C., Greber, B., Ko, K., Sterneckert, J., Han, D.W., Araúzo-Bravo, M.J., and Schöler, H.R. (2011). Distinct developmental ground states of epiblast stem cell lines determine different pluripotency features. Stem Cells *29*, 1496–1503.

Betschinger, J., Nichols, J., Dietmann, S., Corrin, P.D., Paddison, P.J., and Smith, A. (2013). Exit from pluripotency is gated by intracellular redistribution of the bHLH transcription factor Tfe3. Cell *153*, 335–347.

Blakeley, P., Fogarty, N.M.E., Valle, I., Wamaitha, S.E., Hu, T.X., Elder, K., Snell, P., Christie, L., Robson, P., and Niakan, K.K. (2015). Defining the three cell lineages of the human blastocyst by single-cell RNA-seq. Development *142*, 3151–3165.

Boroviak, T., Loos, R., Lombard, P., Okahara, J., Behr, R., Sasaki, E., Nichols, J., Smith, A., and Bertone, P. (2015). Lineage-specific profiling delineates the emergence and progression of naive pluripotency in mammalian embryogenesis. Dev. Cell *35*, 366–382.

Brons, I.G., Smithers, L.E., Trotter, M.W., Rugg-Gunn, P., Sun, B., Chuva de Sousa Lopes, S.M., Howlett, S.K., Clarkson, A., Ahrlund-Richter, L., Pedersen, R.A., et al. (2007). Derivation of pluripotent epiblast stem cells from mammalian embryos. Nature *448*, 191–195.

Chan, Y.S., Göke, J., Ng, J.H., Lu, X., Gonzales, K.A.U., Tan, C.P., Tng, W.Q., Hong, Z.Z., Lim, Y.S., and Ng, H.H. (2013). Induction of a human pluripotent state with distinct regulatory circuitry that resembles preimplantation epiblast. Cell Stem Cell *13*, 663–675.

Chen, H., Aksoy, I., Gonnot, F., Osteil, P., Aubry, M., Hamela, C., Rognard, C., Hochard, A., Voisin, S., Fontaine, E., et al. (2015). Reinforcement of STAT3 activity reprogrammes human embryonic stem cells to naive-like pluripotency. Nat. Commun. *6*, 7095.

Duggal, G., Warrier, S., Ghimire, S., Broekaert, D., Van der Jeught, M., Lierman, S., Deroo, T., Peelman, L., Van Soom, A., Cornelissen, R., et al. (2015). Alternative routes to induce naïve pluripotency in human embryonic stem cells. Stem Cells *33*, 2686–2698.

Dutta, D., Ray, S., Home, P., Larson, M., Wolfe, M.W., and Paul, S. (2011). Self renewal vs. lineage commitment of embryonic stem cells: protein kinase C signaling shifts the balance. Stem Cells *29*, 618–628.

Evans, M.J., and Kaufman, M.H. (1981). Establishment in culture of pluripotential cells from mouse embryos. Nature *292*, 154–156.

Fan, G., Martinowich, K., Chin, M.H., He, F., Fouse, S.D., Hutnick, L., Hattori, D., Ge, W., Shen, Y., Wu, H., et al. (2005). DNA methylation controls the timing of astrogliogenesis through regulation of JAK-STAT signaling. Development *132*, 3345–3356.

Festuccia, N., Osorno, R., Halbritter, F., Karwacki-Neisius, V., Navarro, P., Colby, D., Wong, F., Yates, A., Tomlinson, S.R., and Chambers, I. (2012). Esrrb is a direct nanog target gene that can substitute for nanog function in pluripotent cells. Cell Stem Cell *11*, 477–490.

Ficz, G., Hore, T.A., Santos, F., Lee, H.J., Dean, W., Arand, J., Krueger, F., Oxley, D., Paul, Y.-L., Walter, J., et al. (2013). FGF signaling inhibition in ESCs drives rapid genome-wide demethylation to the epigenetic ground state of pluripotency. Cell Stem Cell *13*, 351–359.

Gafni, O., Weinberger, L., Mansour, A.A., Manor, Y.S., Chomsky, E., Ben-Yosef, D., Kalma, Y., Viukov, S., Maza, I., Zviran, A., et al. (2013). Derivation of novel human ground state naive pluripotent stem cells. Nature *504*, 282–286.

Guo, G., Von Meyenn, F., Santos, F., Chen, Y., Reik, W., Bertone, P., Smith, A., and Nichols, J. (2016). Naive pluripotent stem cells derived directly from isolated cells of the human Inner cell mass. Stem Cell Reports *6*, 437–446.

Guo, H., Zhu, P., Yan, L., Li, R., Hu, B., Lian, Y., Yan, J., Ren, X., Lin, S., Li, J., et al. (2014). The DNA methylation landscape of human early embryos. Nature *511*, 606–610.

Habibi, E., Brinkman, A.B., Arand, J., Kroeze, L.I., Kerstens, H.H.D., Matarese, F., Lepikhov, K., Gut, M., Brun-Heath, I., Hubner, N.C., et al. (2013). Whole-genome bisulfite sequencing of two distinct interconvertible DNA methylomes of mouse embryonic stem cells. Cell Stem Cell *13*, 360–369.

Hall, J., Guo, G., Wray, J., Eyres, I., Nichols, J., Grotewold, L., Morfopoulou, S., Humphreys, P., Mansfield, W., Walker, R., et al. (2009). Oct4 and LIF/Stat3 additively induce Krüppel factors to sustain embryonic stem cell self-renewal. Cell Stem Cell *5*, 597–609.

Hanna, J., Cheng, A.W., Saha, K., Kim, J., Lengner, C.J., Soldner, F., Cassady, J.P., Muffat, J., Carey, B.W., and Jaenisch, R. (2010). Human embryonic stem cells with biological and epigenetic characteristics similar to those of mESCs. Proc. Natl. Acad. Sci. USA *107*, 9222–9227.

Hayashi, Y., Caboni, L., Das, D., Yumoto, F., Clayton, T., Deller, M.C., Nguyen, P., Farr, C.L., Chiu, H.J., Miller, M.D., et al. (2015). Structure-based discovery of NANOG variant with enhanced properties to promote self-renewal and reprogramming of pluripotent stem cells. Proc. Natl. Acad. Sci. USA *112*, 4666–4671.

Honda, A., Hatori, M., Hirose, M., Honda, C., Izu, H., Inoue, K., Hirasawa, R., Matoba, S., Togayachi, S., Miyoshi, H., et al. (2013). Naive-like conversion overcomes the limited differentiation capacity of induced pluripotent stem cells. J. Biol. Chem. *288*, 26157–26166.

Imaizumi, Y., Okada, Y., Akamatsu, W., Koike, M., Kuzumaki, N., Hayakawa, H., Nihira, T., Kobayashi, T., Ohyama, M., Sato, S., et al. (2012). Mitochondrial dysfunction associated with increased oxidative stress and α-synuclein accumulation in PARK2 iPSC-derived neurons and postmortem brain tissue. Mol. Brain *5*, 35.

Jang, H.J., Kim, J.S., Choi, H.W., Jeon, I., Choi, S., Kim, M.J., Song, J., and Do, J.T. (2014). Neural stem cells derived from epiblast stem cells display distinctive properties. Stem Cell Res. *12*, 506–516.

Kawasaki, H., Mizuseki, K., Nishikawa, S., Kaneko, S., Kuwana, Y., Nakanishi, S., Nishikawa, S.I., and Sasai, Y. (2000). Induction of midbrain dopaminergic neurons from ES cells by stromal cell-derived inducing activity. Neuron *28*, 31–40.

Leitch, H.G., McEwen, K.R., Turp, A., Encheva, V., Carroll, T., Grabole, N., Mansfield, W., Nashun, B., Knezovich, J.G., Smith, A., et al. (2013). Naive pluripotency is associated with global DNA hypomethylation. Nat. Struct. Mol. Biol. *20*, 311–316.

Liang, G., and Zhang, Y. (2013). Genetic and epigenetic variations in iPSCs: potential causes and implications for application. Cell Stem Cell *13*, 149–159.

Luo, J., Sladek, R., Bader, J.A., Matthyssen, A., Rossant, J., and Giguère, V. (1997). Placental abnormalities in mouse embryos lacking the orphan nuclear receptor ERR-beta. Nature *388*, 778–782.

Marks, H., Kalkan, T., Menafra, R., Denissov, S., Jones, K., Hofemeister, H., Nichols, J., Kranz, A., Francis Stewart, A., Smith, A., et al. (2012). The transcriptional and epigenomic foundations of ground state pluripotency. Cell *149*, 590–604.

Martello, G., Sugimoto, T., Diamanti, E., Joshi, A., Hannah, R., Ohtsuka, S., Göttgens, B., Niwa, H., and Smith, A. (2012). Esrrb is a pivotal target of the Gsk3/Tcf3 axis regulating embryonic stem cell self-renewal. Cell Stem Cell *11*, 491–504.

Martello, G., Bertone, P., and Smith, A. (2013). Identification of the missing pluripotency mediator downstream of leukaemia inhibitory factor. EMBO J. *32*, 2561–2574.

Martin, G.R. (1981). Isolation of a pluripotent cell line from early mouse embryos cultured in medium conditioned by teratocarcinoma stem cells. Proc. Natl. Acad. Sci. USA *78*, 7634–7638.

Nichols, J., and Smith, A. (2009). Naive and primed pluripotent states. Cell Stem Cell *4*, 487–492.

Nishizawa, M., Chonabayashi, K., Nomura, M., Tanaka, A., Nakamura, M., Inagaki, A., Nishikawa, M., Takei, I., Oishi, A., Tanabe, K., et al. (2016). Epigenetic variation between human induced pluripotent stem cell lines is an indicator of differentiation capacity. Cell Stem Cell *19*, 341–354.

Niwa, H., Ogawa, K., Shimosato, D., and Adachi, K. (2009). A parallel circuit of LIF signalling pathways maintains pluripotency of mouse ES cells. Nature *460*, 118–122.

Okada, Y., Matsumoto, A., Shimazaki, T., Enoki, R., Koizumi, A., Ishii, S., Itoyama, Y., Sobue, G., and Okano, H. (2008). Spatiotemporal recapitulation of central nervous system development by murine embryonic stem cell-derived neural stem/progenitor cells. Stem Cells *26*, 3086–3098.

Osafune, K., Caron, L., Borowiak, M., Martinez, R.J., Fitz-Gerald, C.S., Sato, Y., Cowan, C.A., Chien, K.R., and Melton, D.A. (2008). Marked differences in differentiation propensity among human embryonic stem cell lines. Nat. Biotechnol. *26*, 313–315.

Petropoulos, S., Edsgärd, D., Reinius, B., Deng, Q., Panula, S.P., Codeluppi, S., Reyes, A.P., Linnarsson, S., Sandberg, R., and Lanner, F. (2016). Single-cell RNA-seq reveals lineage and X chromosome dynamics in human preimplantation embryos. Cell *165*, 1012–1026.

Qin, H., Hejna, M., Liu, Y., Percharde, M., Wossidlo, M., Blouin, L., Durruthy-Durruthy, J., Wong, P., Qi, Z., Yu, Z., et al. (2016). YAP induces human naive pluripotency. Cell Rep. *14*, 2301–2312.

Qiu, D., Ye, S., Ruiz, B., Zhou, X., Liu, D., Zhang, Q., and Ying, Q.L. (2015). Klf2 and Tfcp2l1, two Wnt/β-catenin targets, act synergistically to induce and maintain naive pluripotency. Stem Cell Reports *5*, 314–322.

Silva, J., Nichols, J., Theunissen, T.W., Guo, G., van Oosten, A.L., Barrandon, O., Wray, J., Yamanaka, S., Chambers, I., and Smith, A. (2009). Nanog is the gateway to the pluripotent ground state. Cell *138*, 722–737.

Smith, A.G., Heath, J.K., Donaldson, D.D., Wong, G.G., Moreau, J., Stahl, M., and Rogers, D. (1988). Inhibition of pluripotential embryonic stem cell differentiation by purified polypeptides. Nature *336*, 688–690.

Song, L., Chen, J., Peng, G., Tang, K., and Jing, N. (2016). Dynamic heterogeneity of brachyury in mouse epiblast stem cells mediates distinct respond to extrinsic BMP signaling. J. Biol. Chem. *291*, 15212–15225.

Takahashi, K., Tanabe, K., Ohnuki, M., Narita, M., Ichisaka, T., Tomoda, K., and Yamanaka, S. (2007). Induction of pluripotent stem cells from adult human fibroblasts by defined factors. Cell *131*, 861–872.

Takashima, Y., Guo, G., Loos, R., Nichols, J., Ficz, G., Krueger, F., Oxley, D., Santos, F., Clarke, J., Mansfield, W., et al. (2014). Resetting transcription factor control circuitry toward ground-state pluripotency in human. Cell *158*, 1254–1269.

Takizawa, T., Nakashima, K., Namihira, M., Ochiai, W., Uemura, A., Yanagisawa, M., Fujita, N., Nakao, M., and Taga, T. (2001). DNA methylation is a critical cell-intrinsic determinant of astrocyte differentiation in the fetal brain. Dev. Cell *1*, 749–758.

Tesar, P.J., Chenoweth, J.G., Brook, F.A., Davies, T.J., Evans, E.P., Mack, D.L., Gardner, R.L., and McKay, R.D. (2007). New cell lines from mouse epiblast share defining features with human embryonic stem cells. Nature *448*, 196–199.

Theunissen, T.W., Powell, B.E., Wang, H., Mitalipova, M., Faddah, D.A., Reddy, J., Fan, Z.P., Maetzel, D., Ganz, K., Shi, L., et al. (2014). Systematic identification of culture conditions for induction and maintenance of naive human pluripotency. Cell Stem Cell *15*, 471–487.

Thomson, J.A., Itskovitz-Eldor, J., Shapiro, S.S., Waknitz, M.A., Swiergiel, J.J., Marshall, V.S., and Jones, J.M. (1998). Embryonic stem cell lines derived from human blastocysts. Science *282*, 1145–1147.

Tsankov, A.M., Akopian, V., Pop, R., Chetty, S., Gifford, C.A., Daheron, L., Tsankova, N.M., and Meissner, A. (2015). A qPCR ScoreCard quantifies the differentiation potential of human pluripotent stem cells. Nat. Biotechnol. *33*, 1182–1192.

Valamehr, B., Robinson, M., Abujarour, R., Rezner, B., Vranceanu, F., Le, T., Medcalf, A., Lee, T.T., Fitch, M., Robbins, D., et al. (2014). Platform for induction and maintenance of transgene-free hiPSCs resembling ground state pluripotent stem cells. Stem Cell Reports *2*, 366–381.

Vallier, L., Alexander, M., and Pedersen, R.A. (2005). Activin/Nodal and FGF pathways cooperate to maintain pluripotency of human embryonic stem cells. J. Cell Sci. *118*, 4495–4509.

Van der Jeught, M., Heindryckx, B., O'Leary, T., Duggal, G., Ghimire, S., Lierman, S., Van Roy, N., Chuva de Sousa Lopes, S.M., Deroo, T., Deforce, D., and De Sutter, P. (2014). Treatment of human embryos with the TGFβ inhibitor SB431542 increases epiblast proliferation and permits successful human embryonic stem cell derivation. Hum. Reprod. *29*, 41–48.

Wang, J., Xie, G., Singh, M., Ghanbarian, A.T., Raskó, T., Szvetnik, A., Cai, H., Besser, D., Prigione, A., Fuchs, N.V., et al. (2014). Primate-specific endogenous retrovirus-driven transcription defines naive-like stem cells. Nature *516*, 405–409.

Wani, M.A., Means, R.T., Jr., and Lingrel, J.B. (1998). Loss of LKLF function results in embryonic lethality in mice. Transgenic Res. *7*, 229–238.

Ware, C.B., Nelson, A.M., Mecham, B., Hesson, J., Zhou, W., Jonlin, E.C., Jimenez-Caliani, A.J., Deng, X., Cavanaugh, C., Cook, S., et al. (2014). Derivation of naive human embryonic stem cells. Proc. Natl. Acad. Sci. USA *111*, 4484–4489.

Watanabe, K., Ueno, M., Kamiya, D., Nishiyama, A., Matsumura, M., Wataya, T., Takahashi, J.B., Nishikawa, S., Nishikawa, S., Muguruma, K., et al. (2007). A ROCK inhibitor permits survival of dissociated human embryonic stem cells. Nat. Biotechnol. *25*, 681–686.

Williams, R.L., Hilton, D.J., Pease, S., Willson, T.A., Stewart, C.L., Gearing, D.P., Wagner, E.F., Metcalf, D., Nicola, N.A., and Gough, N.M. (1988). Myeloid leukaemia inhibitory factor maintains the developmental potential of embryonic stem cells. Nature *336*, 684–687.

Yan, L., Yang, M., Guo, H., Yang, L., Wu, J., Li, R., Liu, P., Lian, Y., Zheng, X., Yan, J., et al. (2013). Single-cell RNA-seq profiling of human preimplantation embryos and embryonic stem cells. Nat. Struct. Mol. Biol. *20*, 1131–1139.

Yang, Y., Znang, X., Yi, L., Hou, Z., Chen, J., Kou, X., Zhao, Y., Wang, H., Sun, X.F., Jiang, C., et al. (2016). Naïve induced pluripotent stem cells generated from β-thalassemia fibroblasts allow efficient gene correction with CRISPR/Cas9. Stem Cells Transl. Med. *5*, 8–19.

Ye, S., Li, P., Tong, C., and Ying, Q.L. (2013). Embryonic stem cell self-renewal pathways converge on the transcription factor Tfcp2l1. EMBO J. *32*, 2548–2560.

Yeo, J.C., Jiang, J., Tan, Z.Y., Yim, G.R., Ng, J.H., Göke, J., Kraus, P., Liang, H., Gonzales, K.A.U., Chong, H.C., et al. (2014). Klf2 is an essential factor that sustains ground state pluripotency. Cell Stem Cell *14*, 864–872.

Ying, Q.L., Wray, J., Nichols, J., Batlle-Morera, L., Doble, B., Woodgett, J., Cohen, P., and Smith, A. (2008). The ground state of embryonic stem cell self-renewal. Nature *453*, 519–523.

Yusa, K., Zhou, L., Li, M.A., Bradley, A., and Craig, N.L. (2011). A hyperactive piggyBac transposase for mammalian applications. Proc. Natl. Acad. Sci. USA *108*, 1531–1536.

Stem Cell Reports, Volume *9*

Supplemental Information

Naive-like ESRRB^{+} iPSCs with the Capacity for Rapid Neural Differentiation

Fumihiko Kisa, Seiji Shiozawa, Keisuke Oda, Sho Yoshimatsu, Mari Nakamura, Ikuko Koya, Kenji Kawai, Sadafumi Suzuki, and Hideyuki Okano

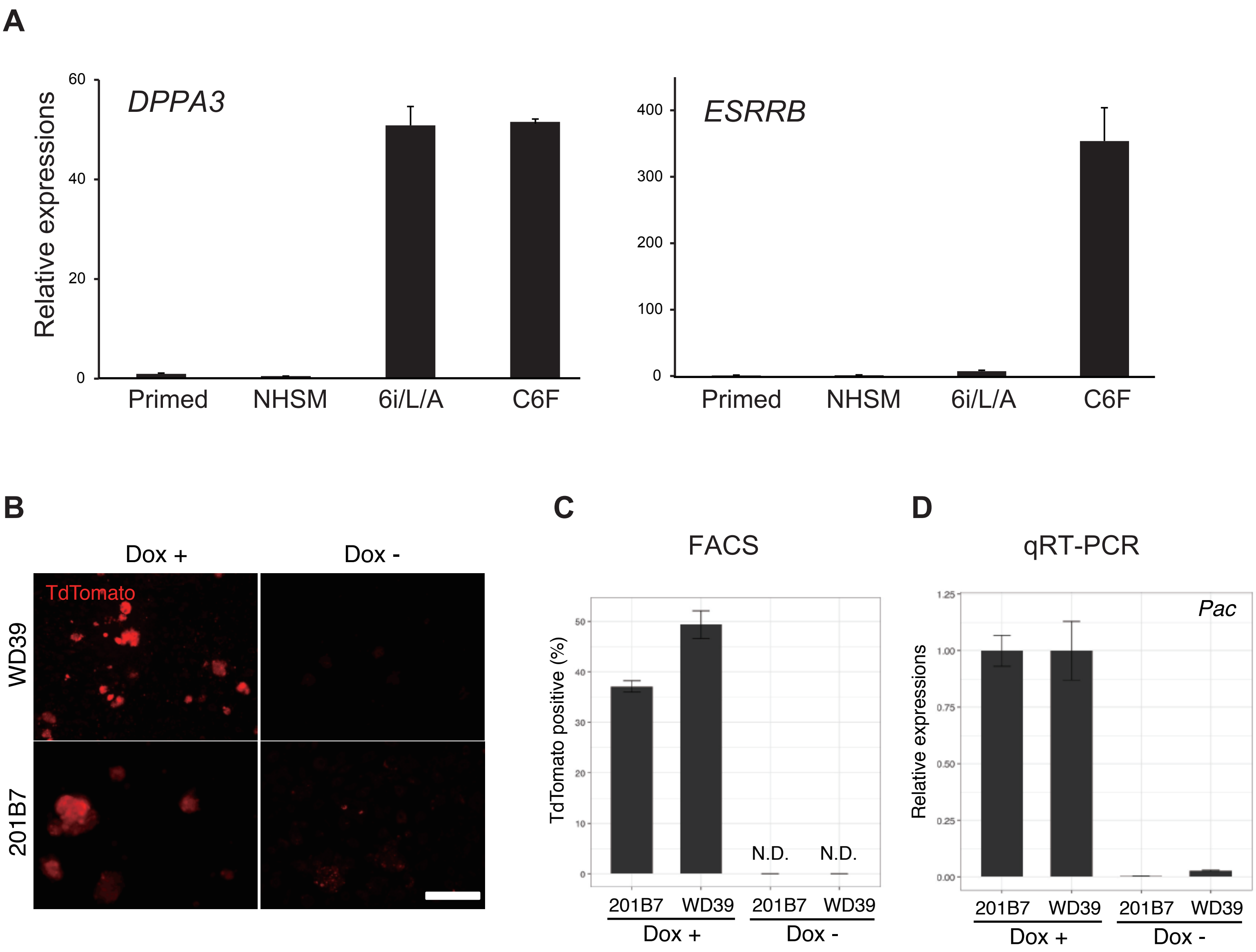

Figure S1. Comparison of C6F method with transgene-free naïve-conversion method and regulation of the C6F transgene expression by Doxycycline. (related to Figure 1 and Figure 3)

(A) qPCR analysis of the expressions of naïve marker genes (*DPPA3* and *ESRRB*) in WD39 cells expressing C6F or maintained in naïve-conversion medium as previously reported (n = 3; mean ± SEM; independent experiments). (B) Transgene expression indicator TdTomato disappeared after removal of doxycycline(Dox). The scale bar represents 100 µm. (C) Flowcytometric analysis of the TdTomato expression (n = 3; mean ± SEM; independent experiments). (D) Quantitative RT-PCR of *puromycin N-acetyl-transferase* (*Pac*) gene before and after Dox removal in N-hiPSCs (n = 3; mean ± SEM; independent experiments).

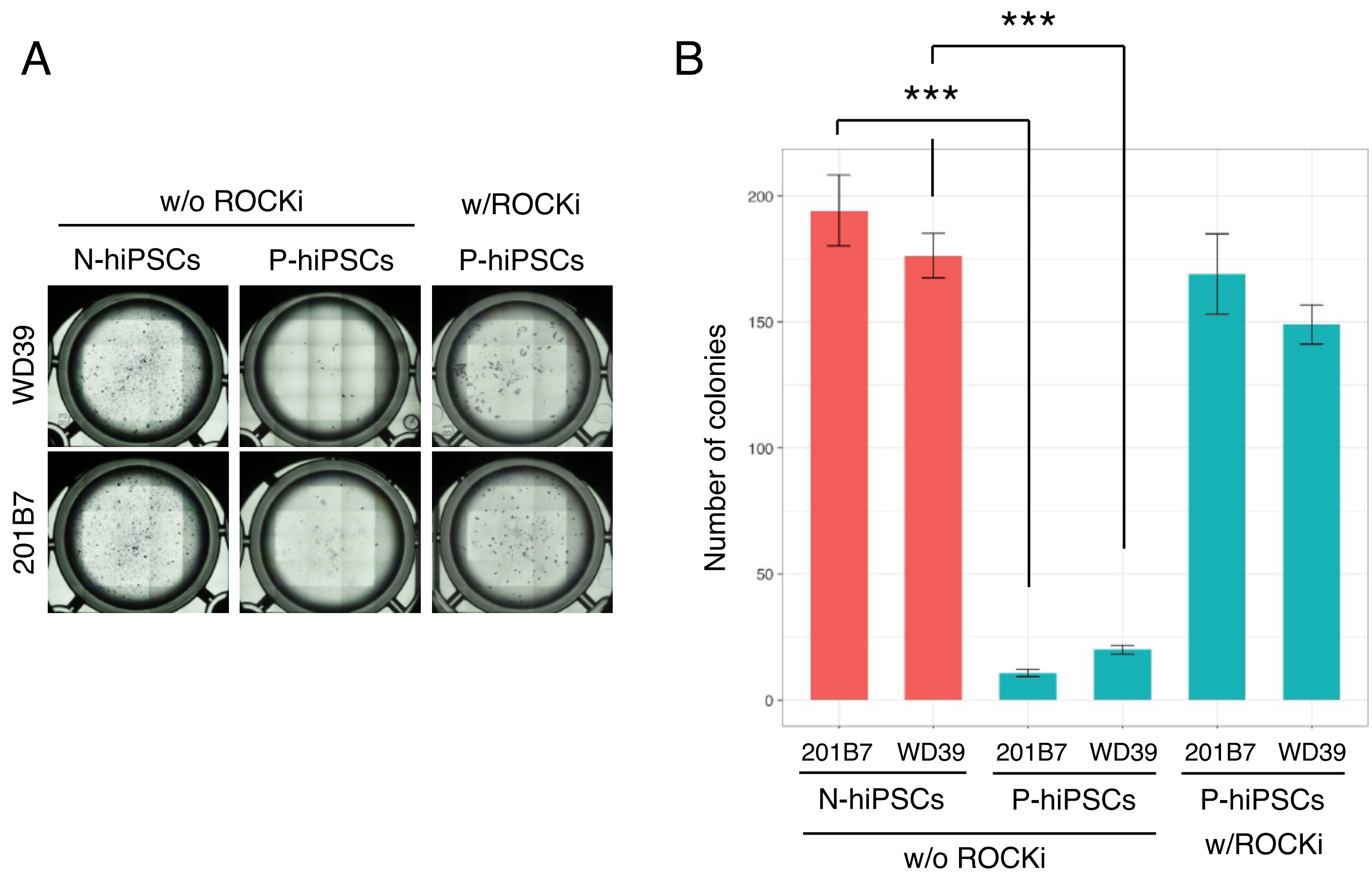

Figure S2. Colony formation assay after single cell dissociation (related to Figure 2)

(A) The number of alkaline phosphatase-positive colonies after single cell dissociation. N-hiPSCs can form colonies without ROCK inhibitor treatment. (B) Quantification of the number of alkaline phosphatase-positive colonies. ($n = 3$; mean ± SEM; independent experiments; ***$P < 0.001$; t test)

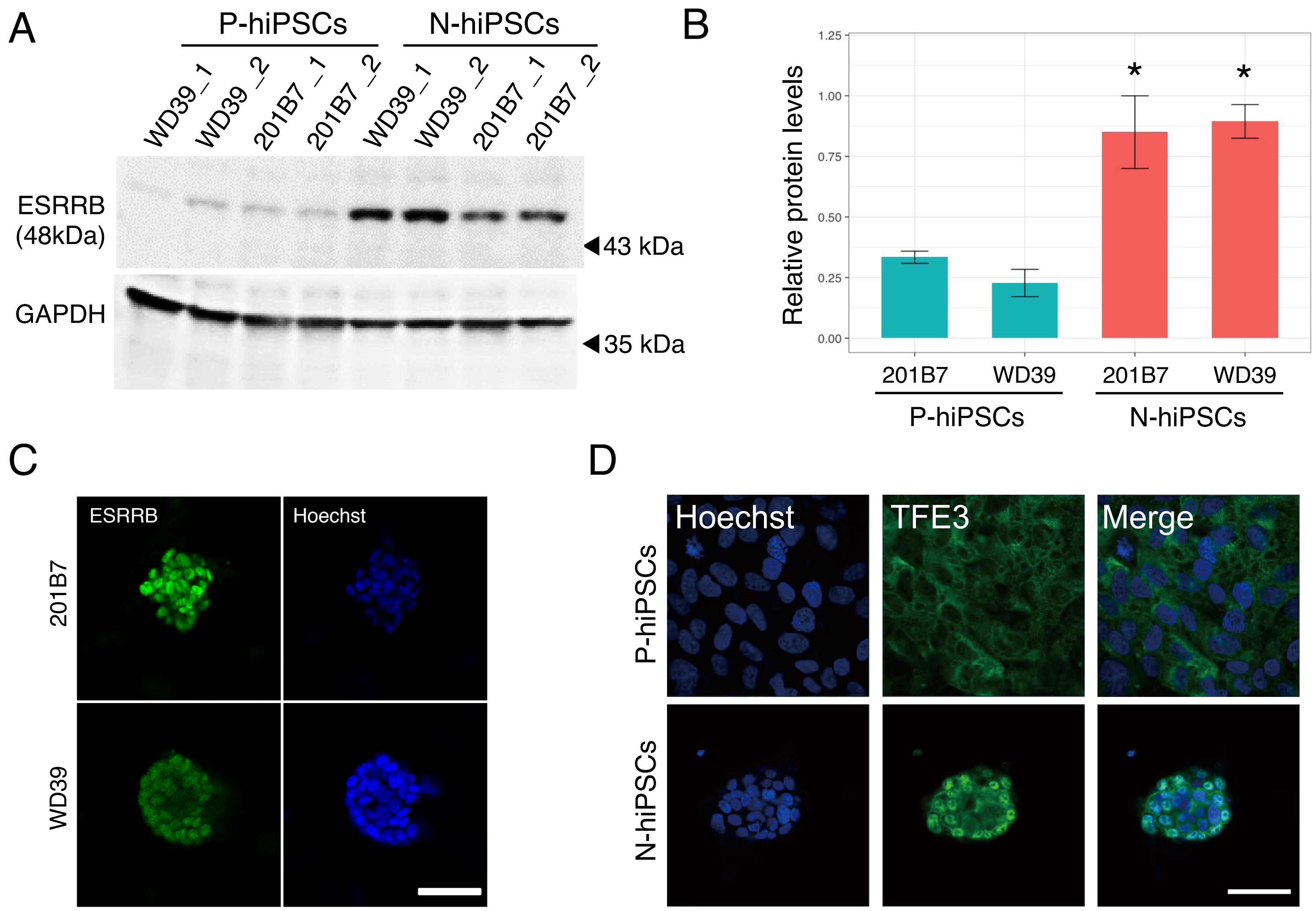

Figure S3. ESRRB protein expression, TFE3 subcellular localization in the N-hiPSCs (related to Figures 2 and 5)

(A) Western blot analysis of ESRRB in the P- and N-hiPSCs. The result of 2 independent samples from each line is presented. (B) Quantification of the GAPDH-normalized ESRRB protein levels from the western blots. Bars indicate the average of the 3 independent samples and error bars indicate SEM. *P < 0.05 (C) ESRRB immunocytochemistry in the N-hiPSCs. The scale bar represents 50 μm. (D) Immunocytochemical analyses of the 201B7-derived P-hiPSCs and N-hiPSCs for TFE3. The scale bar represents 50 μm.

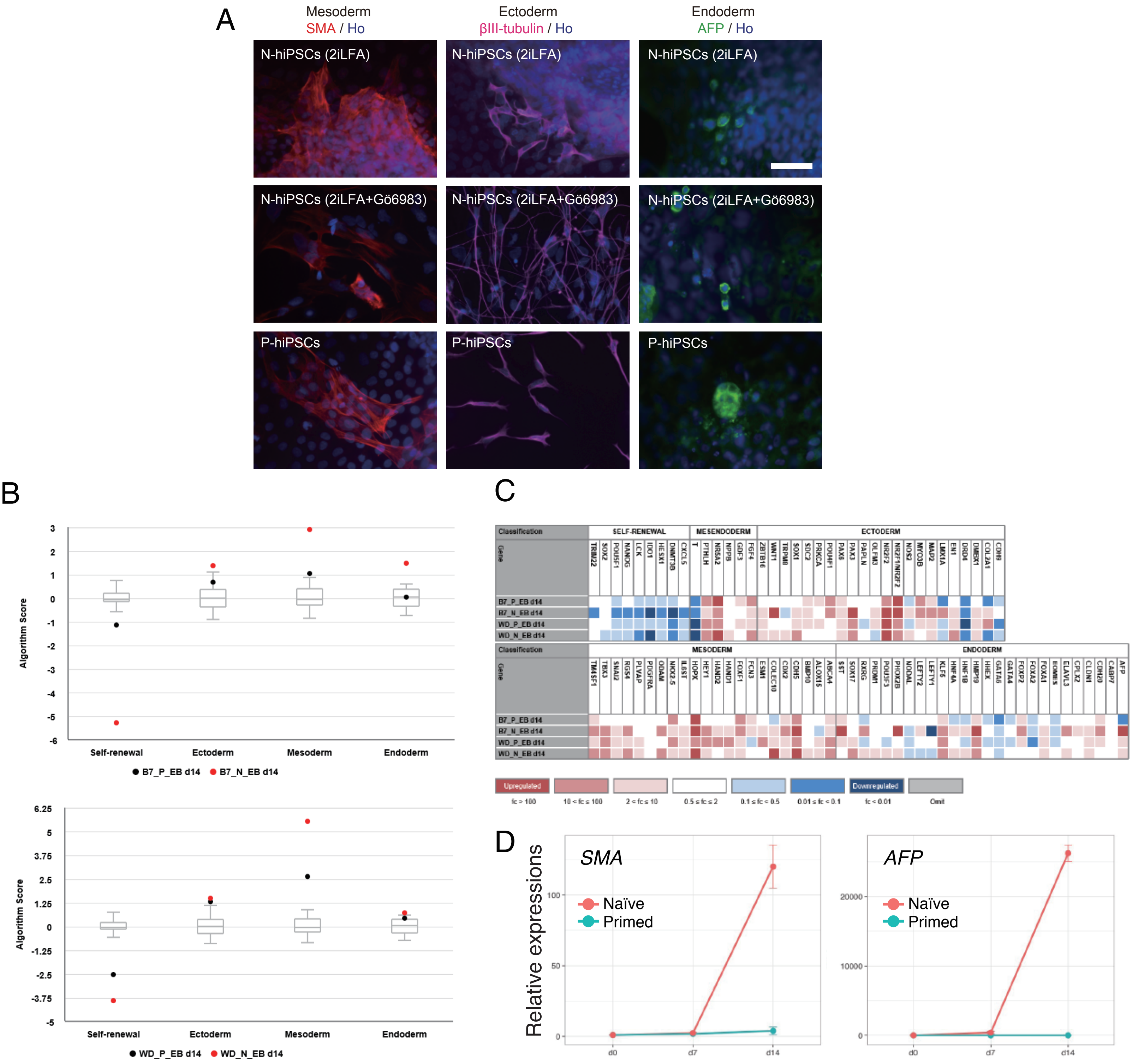

Figure S4. Three germ layer differentiation potential (related to Figures 2 and 3)

(A) Immunocytochemistries of differentiated EBs from 201B7 N-hiPSCs cultured in 2iLFA (upper) or 2iLFA+Go (N2B27) (middle), and their primed counterparts (lower) for smooth muscle actin (SMA), β-III-tubulin and α-fetoprotein (AFP). The scale bar represents 50 μm. (B, C) Quantitative analysis of the trilineage differentiation potential using the TaqMan hPSC scorecard kit. (B) Comparison of the differentiation potentials between N- (red) and P- (black) hiPSCs derived from 201B7 (top) and WD39 (bottom), respectively. The grey box plot indicates the reference data set provided by the manufacturer. The error bars represent the maximum and minimum values of the reference data set. (C) Heat map of each gene expression level relative to the reference. (D) qPCR analysis of gene expression time course in EBs derived from P- and N-hiPSCs (days 0, 7 and 14). (n = 3; mean ± SEM; independent experiments)

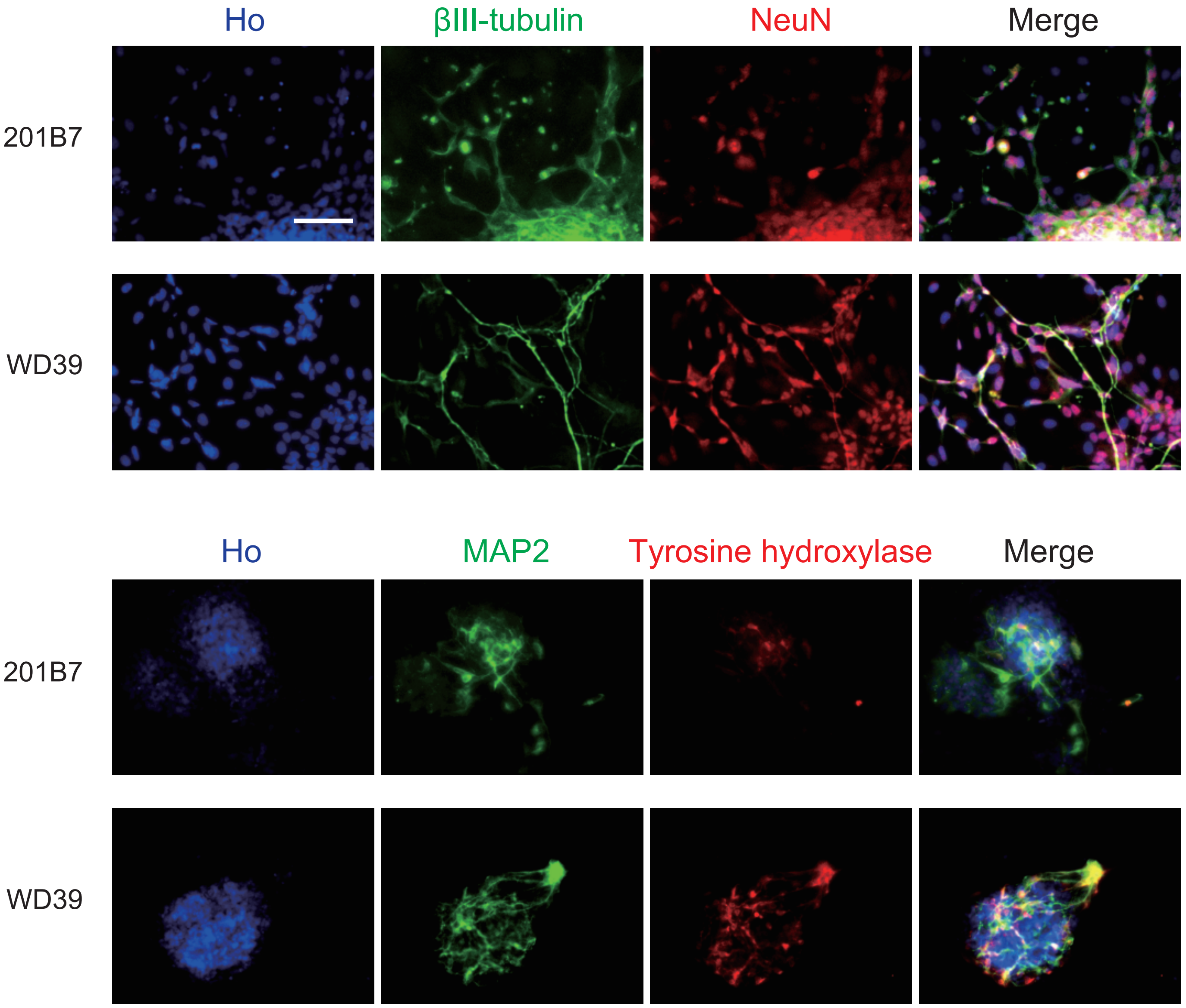

Figure S5. Neuronal differentiation of the primed hiPSCs by the SDIA method over 20 days (related to Figure 6)
Representative immunocytochemical images of SDIA-differentiated colonies using pan-neuronal markers (β-III-tubulin, MAP2, NeuN) and a marker for dopaminergic neurons (tyrosine hydroxylase). The scale bar represents 50 μm.

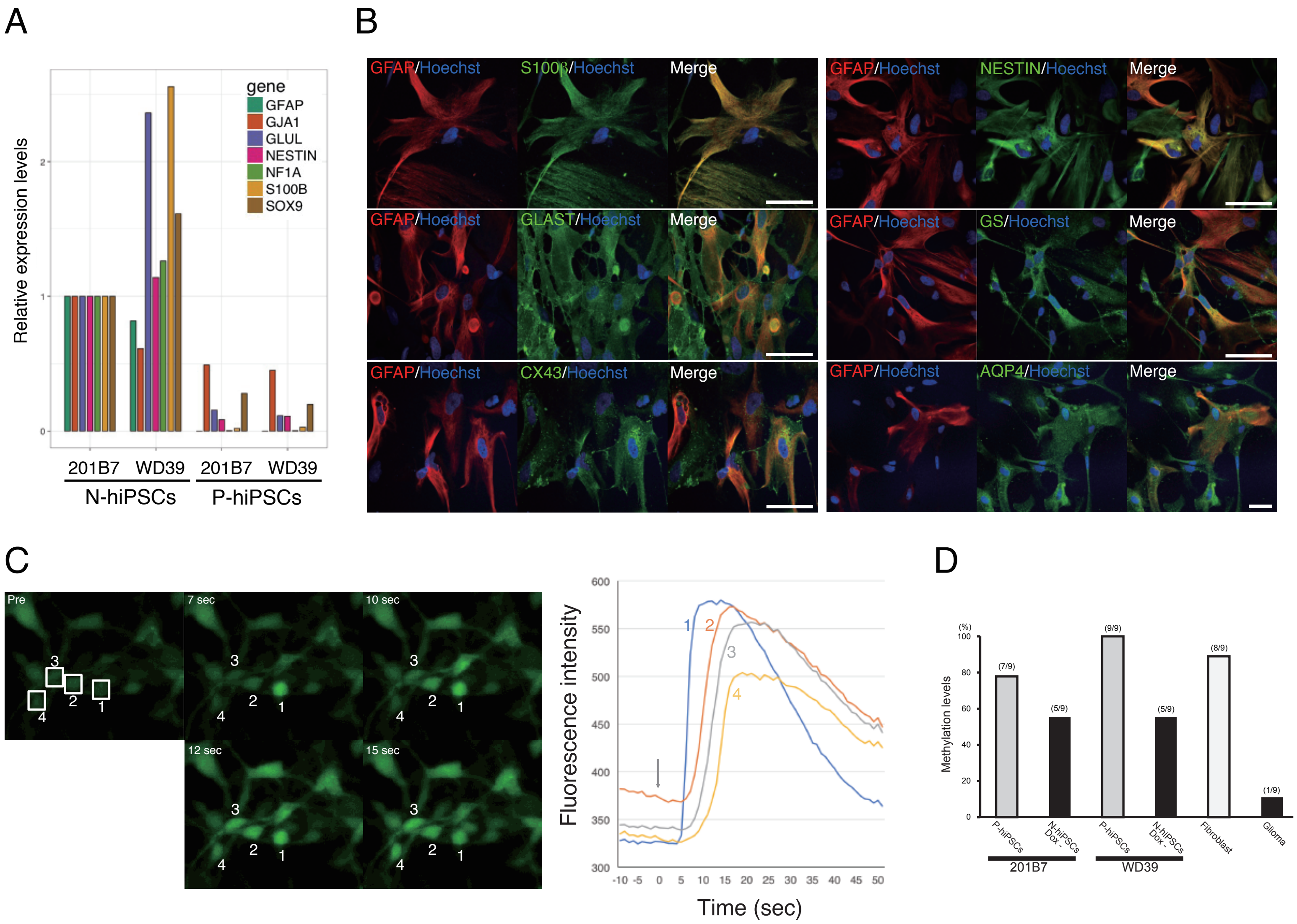

Figure S6. Glial differentiation (related to Figure 6)

(A) qPCR analysis of astrocytic marker expression in the differentiated cells derived from N- and P-hiPSCs using a neurosphere-based method. Bars indicate the average of the technical tripricate. (B) Immunocytochemistry of N-hiPSCs-derived astrocytes for astrocytic markers. The scale bar represents 50 μm. (C) Calcium imaging in the N-hiPSCs-derived astrocytes using a calcium indicator Fluo-4. The left panel shows representative time course images. The right graph indicates changes of fluorescence intensity after ATP administration. The white boxes in the picture from the left panel indicate the quantified cells, whose numbers correspond to the numbers in the right graph. See also Movie S1. Similar results were obtained from 3 independent experiments. (D) Hypomethylation of the STAT3 binding site in the GFAP promoter of the N-hiPSCs. Frequencies of STAT3 binding site methylation in the GFAP promoter as analyzed with cloning approaches.

Table S1. Primer sequences

Gene	Forward	Reverse
endo-NANOG	gacactggctgaatccttcctct	accctccatgagattgactggat
endo-OCT3/4	agtttgtgccagggtttttg	acttcaccttccctccaacc
endo-KLF4	gccagaaagcactacaatcatgg	ttggcattttgtaagtccaggaa
endo-KLF2	gtgggaaaagaccacgatcctcc	tctcacaaggcatcacaagcctc
LEFTY	agctgcacaccctggacctt	gtcattggtgcttcagggtca
DPPA3	HA216940 [Takara perfect real time]	HA216940 [Takara perfect real time]
ESRRB	HA095808 [Takara perfect real time]	HA095808 [Takara perfect real time]
TFCP2L1	HA193431 [Takara perfect real time]	HA193431 [Takara perfect real time]
KLF5	HA224420 [Takara perfect real time]	HA224420 [Takara perfect real time]
TBX3	HA142445 [Takara perfect real time]	HA142445 [Takara perfect real time]
SOCS3	gtgcgccatggtcacccaca	gcttgcgcactgcgttcacc
ACTB	tgaagtgtgacgtggacatc	ggaggagcaatgatcttgat
SMA	gacaatggctctgggctctgtaa	tgtgttcgtcacccacgta
AFP	gtagcgctgcaaacgatgaa	tccaacaggcctgagaaatc
Pac	gaccgagtacaagcccacgg	acgcgcgtgaggaagagttc
GFAP	acatcgagatcgccacctac	cggagcaactatcctgcttc
NESTIN	tggaggcaaagagggttcag	tcggagaactctgtcccca
NF1A	agctcatggagcggcaatag	attcatcctgggtgagacagag
GJA1	HA151076 [Takara perfect real time]	HA151076 [Takara perfect real time]
GLUL	HA279385 [Takara perfect real time]	HA279385 [Takara perfect real time]
S100B	HA168460 [Takara perfect real time]	HA168460 [Takara perfect real time]
SOX9	HA148317 [Takara perfect real time]	HA148317 [Takara perfect real time]

Supplemental Experimental Procedures

Introduction of transgenes

To evaluate the effects of transcription factors on naïve conversion, pPB-T2F/Venus (Dox-inducible transgene carrying NANOG, KLF2 and Venus), pPB-Y4F/Cerulean (Dox-inducible transgene carrying the four Yamanaka factors and Cerulean), pPB-C6F/TdTomato (Dox-inducible transgene carrying the four Yamanaka factors, NANOG, KLF2 and TdTomato) and pPB-Venus (control mock vector) were introduced into hiPSCs with reverse tetracycline transactivator (rtTA) and piggyBac transposase expression vectors using GeneJuice Transfection Reagent [Novagen]. The transfected cells were cultured in primed condition medium containing 1 μg/mL Dox; furthermore, 100 μg/mL G418 (pPB-T2F/Venus) or 1 μg/mL puromycin (pPB-Venus, pPB-Y4F/Cerulean and pPB-C6F/TdTomato) were added for selection. The selected cells were subjected to quantitative PCR (qPCR) analysis after seven days.

For cloning, the transfected cells were selected by adding 100 μg/mL hygromycin for five to seven days. The Dox-inducible expression systems in the cells were verified based on the Dox-inducible TdTomato expression after a short-term addition of Dox. Next, suitable colonies were picked up with a pipette and transferred to a new culture. The cells were expanded in the primed condition.

Naïve conversion

For conversion to the naïve pluripotent state, the C6F transgenic hiPSC colonies were detached from the feeder layers using the dissociation solution and were dissociated into single cells using TrypLE Select [Life Technologies]. The cells were seeded on irradiated mouse embryonic fibroblasts in two different mediums (N2B27 medium: 1:1 mixture of Neurobasal [Life Technologies] and DMEM/F-12, 1% N2 supplement [Life Technologies], 2% B27 supplement [Life Technologies], 1 mM L-glutamine, 1% NEAA, 0.1 mM 2-ME, and 50 μg/mL AlbuMax I [Life Technologies]; KSR medium: KnockOut DMEM [Life Technologies], 20% KSR, 2 mM L-glutamine, 1% NEAA, and 0.2 mM 2-ME) both of which contained 1 μg/mL Dox, 10 ng/mL human LIF [Nacalai tesque], 1 μM CHIR99021 [Axon Medchem], 1 μM PD0325901 [Wako], 10 μM Forskolin [Sigma], and 5 μM A83-01 [Santacruz]. For the transgene independent culture, 5 μM Gö6983 [R&D Systems] was included to enhance conversion toward the naïve pluripotency state and to maintain naïve pluripotency without Dox. Ten micromolars of Y27632 [Sigma] was supplemented for the first 24 hrs of the primed-to-naïve conversion. The converted cells were grown at 37°C under hypoxic conditions (5% O_2, 5% CO_2). The cells were

subcultured using trypsin/EDTA [Invitrogen] every five to six days without Y27632. The Dox could be removed 10 days after the start of the naïve conversion. Additionally, 0.6 μM JAK1 inhibitor [Calbiochem] was used to assess the LIF/STAT3 signal dependency.

Naïve conversion via the medium conditions

hiPSCs were cultured in NHSM (Knockout DMEM containing 1% N2 supplement, 1 mM L-glutamine, 1% NEAA, 0.1 mM 2-ME, 10 mg/mL AlbuMax I, 12.5 μg/mL recombinant human insulin [Wako], 20 ng/mL human LIF, 1 ng/mL TGF-β, 8 ng/mL FGF-2, 3 μM CHIR99021, 1 μM PD0325901, 5 μM Y27632, 10 μM SB203580 [InvivoGen], 10 μM SP600125 [Enzo Life Sciences] and 10 μM Gö6983 [Tocris]) (Gafni et al., 2013) or 6i/L/A (N2B27 medium containing 20 ng/mL human LIF, 1 μM PD0325901, 1 μM IM-12 [Enzo Life Sciences], 0.5 μM SB590885 [R&D Systems], 1 μM WH-4-023 [A Chemtek], 10 μM Y27632, 20 ng/mL Activin A [PeproTech], 0.5% KSR and 8 ng/mL FGF-2) (Theunissen et al., 2014).

Reverse transcription and quantitative PCR analysis

Total RNA was isolated using an RNeasy Mini Kit [QIAGEN] and reverse transcribed using ReverTra Ace [TOYOBO]. The qPCR analysis was performed with the SYBR Green Master Mix [Life Technologies] on a ViiA7 real-time PCR platform [Applied Biosystems] according to the manufacturer' s instruction. The data are presented as the relative mRNA expression levels normalized by *ACTB*. The RT-PCR primers used herein are provided in Table S1.

RNA-seq

Total RNA was extracted using an RNeasy Mini Kit. The qualities and quantities of the RNA preparations were assessed using a 2100 Bioanalyzer with an RNA 6000 Nano LabChip Kit [Agilent Technologies]. Poly(A)+ RNA was selected and converted to a library of cDNA fragments (200–250 bp) with adaptors attached to both ends for sequencing using a TruSeq Stranded mRNA LT Sample Prep Kit Set v2 [Illumina] as per the manufacturer's instructions. The libraries were quantified using a Bioanalyzer DNA High Sensitivity Kit [Agilent Technologies] and a Kapa Library Quantification Kit [Kapa Biosystems] using an Applied Biosystems StepOne Real-Time PCR System according to the manufacturer's instructions. The libraries were then loaded into a flow cell for cluster generation using the TruSeq Rapid SR Cluster Kit [Illumina] and sequenced using an Illumina HiSeq2500 to obtain 51-nucleotide sequences (single-end). Sequencing reads from published RNA-seq experiments were obtained from the GEO

(GSE36552) or the ArrayExpress (E-MTAB-2857, E-MTAB-3929 and E-MTAB-4461) databases. For comparison with our RNA-seq data, raw counts of the published RNA-seq experiments and our experiment were processed as follows. Poor-quality reads (scores < 20) and adapter sequences were trimmed using the Trimmomatic [Bolger et al. 2014]. The remaining reads were cut to 43 bases and shorter reads (length < 30) were removed. The resulting reads were mapped to the UCSC human genome 19 using sailfish version 0.7.6 and the reads mapped in rRNA were omitted. The quantities of the transcripts were measured in templates per million (TPM). ExAtlas (https://lgsun.irp.nia.nih.gov/exatlas/) was used to analyze global correlations between gene expression data. The principal components were computed with the princomp function of the R package. R packages were used to construct the gene expression plots, perform the principal component analyses and create the heatmaps. RNA-seq data has been registered in the GEO:GSE104583.

Bisulfite sequencing

Genomic DNAs from BJ human fibroblasts [ATCC], the U-87 human glioma cell line [ATCC], P-hiPSCs, N-hiPSCs and the original hiPSC clones (201B7 and WD39) were prepared using DNeasy Blood and Tissue Kits [QIAGEN]. One to two micrograms of genomic DNA was modified by treatment with sodium bisulfite using an EpiTect Bisulfite kit [QIAGEN]. A region in the GFAP promoter containing the STAT3 binding site was then amplified by PCR from the bisulfite-treated genomic DNA. The PCR products were analyzed with pyrosequencing and cloning approaches. For the pyrosequencing approach, PCR was performed with the PyroMark PCR kit [QIAGEN] using the following forward and reverse primers, which were designed using PyroMark Assay Design Software [QIAGEN]: hGFAP promoter-forward (5' -GGGTTTTTTTTTATGTTTAGTGAATGAT-3') and biotin-conjugated hGFAP promoter-reverse (5' -ATCCCAAAATACCAAACTATCAAAC-3'). Pyrosequencing was performed with hGFAP promoter-sequence primer (5' -CGTATTTTAGTTTTT-3') on the PyroMark Q24 platform [QIAGEN] according to the manufacturer' s instructions. For the cloning approach, the PCR products from hGFAP promoter-forward primer 2 (5' -TTGGGGAGGAGGTAGATAGTTAGGTTTT-3') and hGFAP promoter- reverse primer 2
(5' -CATCCCCTAATCCCCTTTCCTAAA-3') were cloned into a pCR™-Blunt II-TOPO® vector [Invitrogen], and 9 clones from each sample that were randomly selected were sequenced.

Immunocytochemical analysis

The cells were fixed with 4% paraformaldehyde (PFA) for 15–30 min at room temperature. After incubation with blocking buffer (PBS containing 5% fetal bovine serum (FBS) and 0.3% Triton X-100) for 30–60 min at room temperature (RT), the cells were incubated with primary antibodies at 4°C overnight. For the ESRRB and TFE3 staining, we used commercially available blocking buffer [Thermo Scientific]. For the TFCP2L1 staining, we used PBS-based blocking buffer containing 10% goat serum and 0.05% Tween 20. After the incubation with the primary antibodies, the cells were washed with PBS (-) three times and were then incubated with Alexa 488-, Alexa 555-, or Alexa 647-conjugated secondary antibodies [Life Technologies] for 1 hr at RT. Additionally, the nuclei were stained with 10 mg/mL Hoechst 33258 [Sigma]. After three washes with PBS(-), the cells were mounted on slides and examined with a universal fluorescence microscope [Axiophoto; Carl Zeiss] or a confocal laser scanning microscope [LSM700; Carl Zeiss]. The primary antibodies used in these analyses were as follows: β-III-tubulin (1:1,000; T8660, Sigma), GFAP (1:500; 13-0300, Thermo Fisher Scientific), MAP2 (1:1,000; M4403,Sigma), S100B (1:500; S2532, Sigma), Nestin (1:500; described previously (Kanemura et al., 2002; Nakamura et al., 2003)), AQP4 (1:200; sc-20812, Santacruz), Glutamine synthase (1:500; 610518, BD Biosciences), Cx43 (1:500; ab11370, abcam), GLAST (1:10; 130-095-814, Miltenyi Biotec), ESRRB (1:2000; PP-H6705, Perseus Proteomics Inc.), TFE3 (1:100; HPA023881, Sigma), TFCP2L1 (1:400; ab123354, Abcam), α-fetoprotein (AFP; 1:250; MAB1368, R&D Systems), smooth muscle actin (SMA; 1:150; A2547, Sigma), NeuN (1:100; MAB377, Millipore), and tyrosine hydroxylase (1:500; AB152, Millipore).

Western Blot Analysis

Protein was extracted from P- and N-hiPSCs using RIPA buffer, supplemented with a protease inhibitor cocktail [Roche]. 10μg of total protein were separated by a 7.5-15% SDS-polyacrylamide (PAGE) gel and transferred to a PVDF membrane. After blocking, the membrane was incubated with an anti-ESRRB antibody. Immunoreactive bands were hybridized with an anti-mouse horseradish and visualized with the ECL™ Prime Western Blotting Detection Reagent. Images were acquired with LAS-4000.

Colony formation assays

P-iPSCs and N-iPSCs from the 201B7 and WD39 lines were dissociated into single cells with trypsin-EDTA. $1x10^5$ cells were seeded on triplicate wells with MEF feeder cells, and cultured either with or without ROCK inhibitor treatment. About 7 days after

plating, the cells were fixed and stained for Alkaline Phosphatase using the sigmaFAST BCIP/NBT kit [Sigma]. The number of colonies positive for the staining was counted manually.

Embryonic body (EB) formation

N-hiPSC and P-hiPSC colonies were detached from the feeder layers en bloc using the dissociation solution and by pipetting, respectively. For EB formation, the clusters of undifferentiated iPSCs were incubated for two weeks in DMEM containing 15% FBS, 2 mM L-glutamine, 1% NEAA, and 0.1 mM 2-ME by suspension culture. For further differentiation, the EBs were plated on poly-L-ornithine/fibronectin-coated glass coverslips for an additional week of culture in the same medium. The EBs were fixed with 4% PFA and were subjected to immunocytochemistry analyses with anti-α-fetoprotein, anti-ß-III-tubulin and anti-SMA antibodies. These experiments were performed under atmospheric oxygen conditions (20% O2, 5% CO2).
TaqMan hPSC Scorecard analysis [Thermo Fisher Scientific] was performed according to the manufacturer' s instruction. The data were analysed on hPSC Scorecard analysis software.

Flow cytometry analysis

For evaluating SSEA4 protein expressions, the hiPSCs were dissociated into single cells using TrypLE Select, pelleted and washed with PBS. The cells were then incubated with an antibody against SSEA4 [560796, BD Biosciences] on ice for 30 minutes. The cells that were positive for SSEA4 were isolated by fluorescence-activated cell sorting on a FACS Aria [Becton Dickinson] instrument. Unstained cells were used as a negative control. For evaluating TdTomato protein expressions, TdTomato fluorescence was directly analyzed by FACS Aria. P-hiPSCs were used as a negative control.

Neural differentiation assay

Transgene-independent cells were used for the neural differentiation assay of naïve hiPSCs by removing dox for at least five days before neural induction. The stromal cell-derived inducing activity (SDIA) methods were performed according to a modification of the method described in (Kawasaki et al., 2000). Briefly, the PA6 cells were maintained in α-MEM medium [Life Technologies] containing 10% FBS. PA6 cells plated on gelatin-coated plates were used as a feeder cell layer. To induce neural differentiation, primed and naïve hiPS cells that were pretreated with Y27632 were dissociated into single cells. The cells were grown at densities of 4×10^3–1.2×10^4

cells/well in 12-well plates in GMEM [Sigma] containing 10% KSR, 1 mM sodium pyruvate [Sigma], 1% NEAA and 0.1 mM 2-ME on a PA6 feeder layer. The medium was changed every two or three days. After 10 days, the cells were passaged using TrypLE Select or fixed with 4% PFA. The passaged cells were cultured under the same conditions. The fixed cells were subjected to immunocytochemistry analysis with an anti-MAP2 antibody. For the quantitative analysis, the numbers of colonies containing at least 10 MAP2-positive cells were counted, and the results are presented as the percentages of the total colonies. All (or up to 100) colonies were examined in each experiment. Moreover, the neurosphere-based neural differentiation assays were performed according to a modification of the method described in (Okada et al., 2008). Briefly, the primed and naïve hiPSC colonies were detached en bloc using the dissociation solution and by pipetting, respectively, (day 0) and cultured as suspensions in bacteriological dishes to form EBs. The EBs were cultured in basal medium for each hiPSC culture (hESC medium without FGF-2 for the primed iPSC colonies and N2B27 medium for the naïve iPSC colonies). One hundred nanomolar LDN193189 [StemRD] were added to enhance the neural induction (day 0-7). On day 7, the EBs were enzymatically dissociated into single cells using TrypLE Select, and the dissociated cells were cultured in suspension at a density of 2×10^5 cells/mL in media hormone mix (MHM) medium with 2% B27 supplement and 20 ng/ml FGF-2 to form the primary neurospheres. For the secondary neurospheres, the primary neurospheres were dissociated into single cells using TrypLE Select and cultured at 2×10^5 cells/mL in MHM with B27 supplement and FGF-2 (day 14). To assay the differentiation, the neurospheres were plated on poly-L-ornithine/fibronectin-coated cover glasses and allowed to differentiate in MHM medium containing 2% FBS and 20 ng/mL human LIF for 10 days (days 21-31). The differentiated neurospheres were fixed with 4% PFA and subjected to immunocytochemical analyses with anti-ß-III-tubulin and anti-GFAP antibodies. For the quantitative analysis, the numbers of β-III-tubulin-positive cells and GFAP-positive cells were counted at the peripheral parts of the attached neurospheres. Consequently, 473 cells from the 201B7-P-hiPSCs, 538 cells from the 201B7-N-hiPSCs, 1522 cells from the WD39-P-hiPSCs and 1080 cells from the WD39-N-hiPSCs were assessed.

Calcium imaging

Calcium imaging was performed in N-hiPSCs-derived astrocytes using a calcium indicator Fluo4 [Dojindo], according to the manufacturer' s instruction. Briefly, neural cells were differentiated from N-hiPSCs using the neurosphere-based method as

described above. The culture was trypsinized and replated onto laminin-coated 96well-plates. Subsequently, the cells were treated with Ara-C to ablate neurons. After stimulating the cells with 1mM ATP, time lapse imaging of the fluo-4 fluorescence was performed. Changes in the fluo-4 fluorescence intensity were quantified with the Metamorph software [Moleculardevices].

Supplemental References

Kanemura, Y., Mori, H., Kobayashi, S., Islam, O., Kodama, E., Yamamoto, A., Nakanishi, Y., Arita, N., Yamasaki, M., Okano, H., Hara, M., and Miyake, J. (2002). Evaluation of in vitro proliferative activity of human fetal neural stem/progenitor cells using indirect measurements of viable cells based on cellular metabolic activity. J. Neurosci. Res. 69, 869-879.

Nakamura, Y., Yamamoto, M., Oda, E., Yamamoto, A., Kanemura, Y., Hara, M., Suzuki, A., Yamasaki, M., and Okano, H. (2003). Expression of tubulin beta II in neural stem/progenitor cells and radial fibers during human fetal brain development. Lab. Invest. 83, 479-489.
